# Copper-Promoted Intramolecular Oxidative Dehydrogenation for Synthesizing Dihydroisocoumarins and Isocoumarins

**DOI:** 10.3390/molecules28176319

**Published:** 2023-08-29

**Authors:** Qiang Zhang, Lin-Yan Zhang, Xian-Ying Shi

**Affiliations:** 1 Shaanxi Key Laboratory of Catalysis, School of Chemistry and Environmental Science, Shaanxi University of Technology, Hanzhong 723001, China; zhangqiang22@snut.edu.cn; 2 Key Laboratory of Syngas Conversion of Shaanxi Province, Key Laboratory for Macromolecular Science of Shaanxi Province, School of Chemistry & Chemical Engineering, Shaanxi Normal University, Xi’an 710062, China; azhanglinyan@hotmail.com

**Keywords:** dihydroisocoumarin, isocoumarin, carboxyl-directed, C–O bond coupling

## Abstract

Isocoumarins and dihydroisocoumarins are important skeletons with a wide range of biological activities, such as anti-bacterial, anti-allergy, anti-fungal, anti-tumor, and anti-HIV properties. Herein, we demonstrated divergent syntheses of isocoumarins and 3,4-dihydroisocoumarins by intramolecular dehydrogenative cyclization of 2-(3-oxobutyl) benzoic acids. This transformation undergoes C*sp*^3^–H bonds and O–H bonds coupling in air using copper salt. The reactions may undergo free radical process.

## 1. Introduction

In recent years, oxygen-containing heterocyclic compounds have emerged as the mainstream in drug research and development because of their unique structural characteristics and physiological activities. Compounds with isocoumarins and dihydroisocoumarins, which are important skeletons of many natural products, bioactive substances, and agricultural chemicals, have a wide range of biological activities, such as anti-bacterial and anti-allergy, anti-fungal, anti-tumor, and anti-HIV, and can be used to make herbicides [1,2,3,4,5].

**Figure 1:**
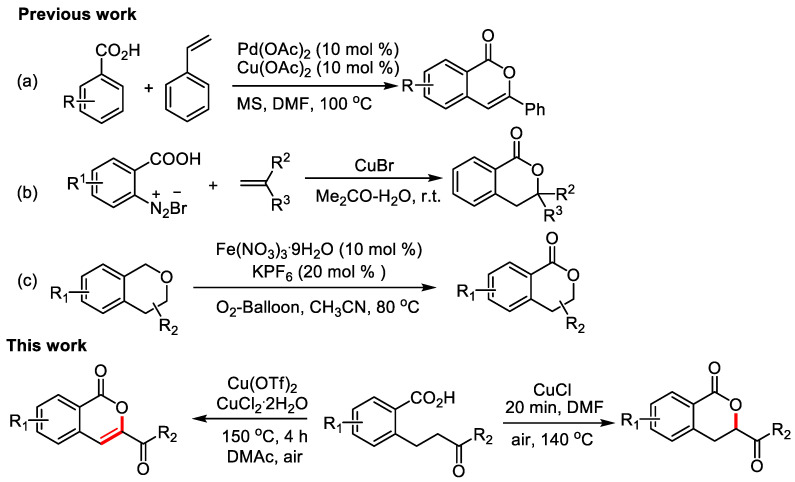
The works for the synthesis of isocoumarins and dihydroisocoumarins. (**a**) Miura’s, work; (**b**) Obushak’s works; (**c**) Shen’s work.

The synthesizing isocoumarins mainly depended on the coupling of carboxyl groups and unsaturated compounds, which involved metal-catalyzed reactions as well as various intramolecular and intermolecular cyclization reactions [6,7,8]. In 1998, Miura’s group [9] firstly employed palladium as a catalyst to synthesize isocoumarins (Figure 1a). In 2009 and 2014, Obushak’s group [10,11] reported the one-pot synthesis of dihydroisocoumarin derivatives and 3-substituted methyl 3,4-dihydroisocoumarin-6-carboxylates under Meerwein’s arylation conditions (Figure 1b). In 2017, Shen’s group [12] discolsed a Fe(NO_3_)_3_-catalyzed synthesis of dihydroisocoumarin derivatives (Figure 1c). In 2018, Oh’s group [13] demonstrated A rhodium-catalyzed decarbonylative aerobic oxidation of cyclic α-diketones for the formations of isocoumarins. Among these reactions, transition metal complexes, such as palladium [14,15,16,17,18,19,20], rhodium [21,22,23,24,25,26,27,28,29,30,31], ruthenium [32,33,34], iridium [35], nickel [36], and silver [37], are commonly used catalysts. Moreover, these protocols have the disadvantages of requiring expensive catalysts and halogenated raw materials, and low atomic utilization, etc.

The synthesis of dihydroisocoumarins is rarely reported. The methods reported in the literature are mainly constructed via the oxidation of methylene groups. Oxygen [12], iridium [35,38], iron [12,39,40], ruthenium [32,33,34,41], manganese [42] can oxidize methylene in isochroman to synthesize dihydroisocoumarins. However, this kind of reaction is not suitable for isocoumarin substrates. Metal-catalyzed intramolecular and intermolecular cyclization reactions, coupling of carbon monoxide with alcohols, and palladium-catalyzed carbonyl insertion have also been used for the synthesis of dihydroisocoumarins. 

Our previous study [43] displayed that 2-(3-oxobutyl) benzoic acid can be generated from aromatic carboxylic acids and 1-penten-3-one in one pot via rhodium-catalyzed carboxyl-directed conjugate addition of C–H bonds to α,β-unsaturated ketones in air and water. We envision that whether 2-(3-oxobutyl) benzoic acid can undergo intramolecular cyclization reaction and oxidative dehydrogenation to yield dihydroisocoumarins and isocoumarins in one pot. Herein, we illuminated copper-promoted intramolecular oxidative dehydrogenation of 2-(3-oxoalkyl) benzoic acid for synthesizing dihydroisocoumarins and isocoumarins in a step in air. 

## 2. Results

### 2.1. General Method for the Synthesis of Dihydroisocoumarins

CuCl (9.9 mg, 0.1 mmol), 0.6 mL of *N*,*N*-dimethylformamide, substituted benzoic acid (0.1 mmol) were added in sequence to a microwave reactor. The reaction tube was directly sealed and reacted at 140 °C (oil bath temperature) for 20 min. Then, the mixture was cooled to room temperature and diluted with ethyl acetate, and the salt was removed through a short silica gel column. The crude product was purified using preparative thin-layer chromatography to give the corresponding product. 

### 2.2. General Method for the Synthesis of Isocoumarins

Cu(OTf)_2_ (217.0 mg, 0.6 mmol), CuCl_2_·2H_2_O (17 mg, 0.1 mmol), 0.6 mL of *N*,*N*-dimethylacetamide, and substituted benzoic acid (0.2 mmol) were added in the microwave reactor. The mixture reacted at 150 °C (oil bath temperature) for 4 h. After cooling to room temperature, the mixture was diluted with ethyl acetate, and the salt was removed through a short silica gel column. The crude product was purified using preparative thin-layer chromatography to give the corresponding product.

## 3. Materials and Methods

### Experimental Reagents and Instruction

^1^H NMR and ^13^C NMR spectra were measured on a Bruker spectrometer, using CDCl_3_ as the solvent with tetramethylsilane (TMS) as an internal standard at room temperature. High-resolution mass spectrometry was determined using a compass-maxis high-resolution mass spectrometer from Bruker Company, Germany. All solvents used in the experiment were dried using activated molecular sieves, and the other reagents used in the experiment were all analytically pure without any other treatment. Chemical shifts are given in δ relative to TMS, and the coupling constants J are given in Hz. Characterization data of compounds, the conversions of acids and NMR spectra of compounds, See Supplementary Materials.

## 4. Discussion

2-methyl-6-(3-oxopentyl)benzoic acid was selected as substrate to screen the reaction conditions (Table 1). To our delight, 4% yield of the dihydroisocoumarin product **2a** was observed at 150 °C for 24 h with CuI. The **2a** were not observed in the atmosphere of nitrogen and oxygen. Using CuCl or CuBr instead of CuI, the yields were increased to 28% and 18% (Table 1, entries 4, 5), only 1% yield was detected using CuF_2_. Other bivalent coppers, such as CuO, Cu(OAc)_2_, and CuBr_2_, failed to generate cyclization product (Table 1, entries 7–9). Lower yields were detected in DMAc, DMSO, THF or *tert*-pentanol (Table 1, entries 10–13). No **2a** were observed in toluene and 1,4-dioxane (Table 1, entries 14, 15). The yield increased to 35% when the amount of CuCl was doubled. It was found that increasing reaction temperature to 140 °C and shortening reaction time to 20 min, the yield of **2a** was enhanced to 61% (Table 1, entry 17). Then, the effects of reaction time on the yield were investigated. A reaction time of 20 min was the best among 10 min, 20 min, and 30 min (Table 1, entries 18–20).

When CuO was added, an isocoumarin product **3a** in 25% yield was observed (Table 2, entry 1). 16% and 12% yields of **3a** were observed in nitrogen and oxygen atmospheres, respectively (Table 2, entries 2, 3). When the solvents were screened (Table 2, entries 4–8), only DMAc gave a slight increase yield (32%, Table 2, entry 4). The yield was 33% when CuCl was replaced by CuCl_2_·2H_2_O (Table 2, entry 9). Other copper salts, such as Cu(OH)_2_, CuCl_2,_ and CuBr, also can deliver **3a** (Table 2, entries 10–12). The yields was found to be 50%, 46% and 26% using Cu(OTf)_2_, AgOTf and AgOAc, respectively (Table 2, entries 13–15). The yield was enhanced to 59% with 3.0 eq. Cu(OTf)_2_ and 0.5 eq. CuCl_2_·2H_2_O at 150 °C for 2.5 h (Table 2, entry 16). It is pleased to find that 65% yield of **3a** was observed when the amount of **1a** was increased to 0.2 mmol (Table 2, entry 17).

**Figure 2:**
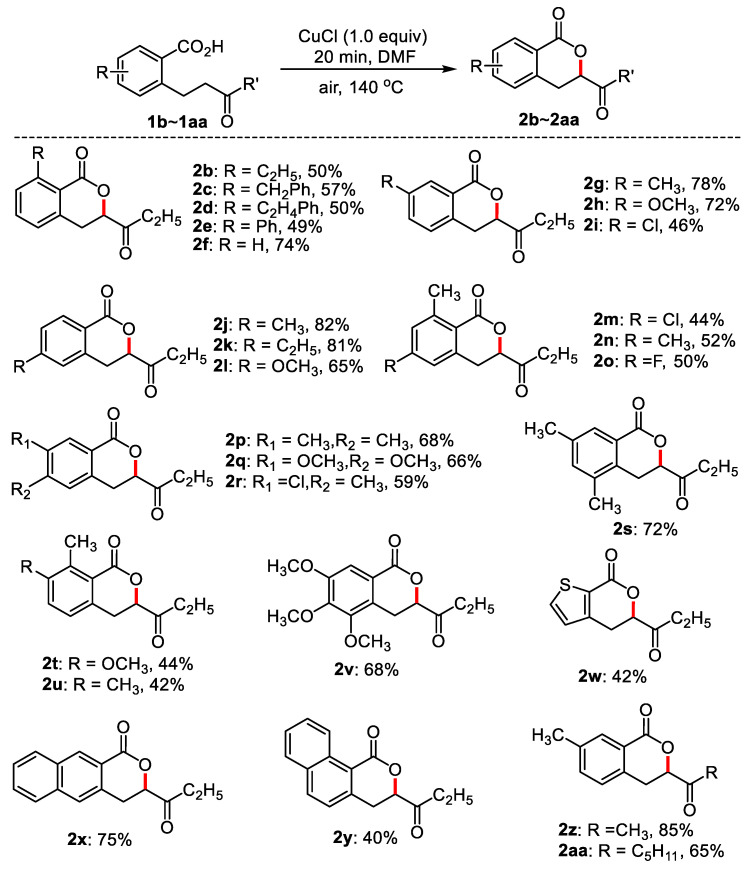
The synthesis of dihydroisocoumarins. Reaction conditions: substituted benzoic acid (0.1 mmol), CuCl (0.1 mmol), DMF (0.6 mL), 140 °C, 20 min, air, isolated yield.

**Figure 3:**
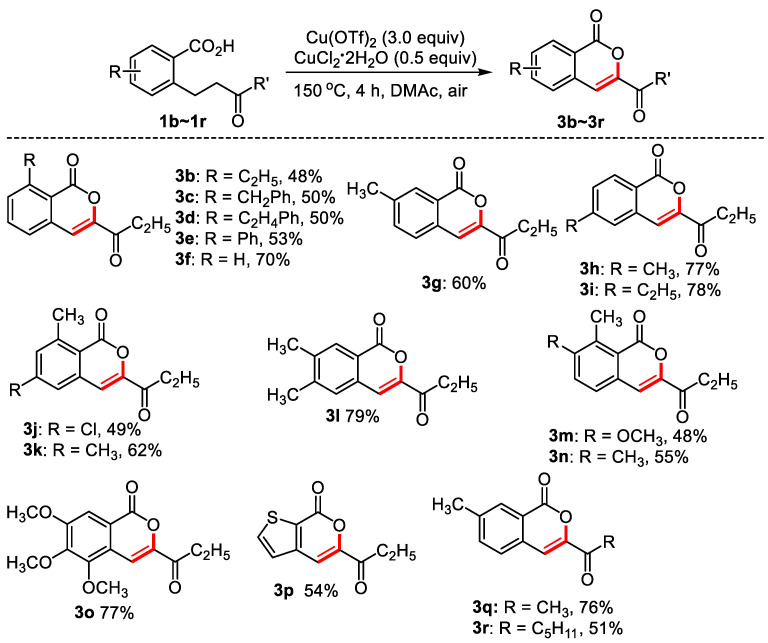
The synthesis of isocoumarins. Reaction conditions: substituted benzoic acid (0.2 mmol), Cu(OTf)_2_ (0.6 mmol), CuCl_2_·2H_2_O (0.1 mmol), DMAc (0.6 mL), 150 °C, 4 h, air, isolated yield.

With the optimum reaction conditions in hand, the application of this method was investigated with a series of substituted 2-(3-oxobutyl) benzoic acids. The results are listed in Figure 2 and Figure 3. As can be seen from Figure 2, substituted 2-(3-oxo-amyl) benzoic acids bearing electron-donating groups at *ortho*-position of carboxyl such as ethyl, phenyl, benzyl, and ethylphenyl delivered moderate yields (**2b**–**2e**, 49–57%), and the yield was 74% when there was no substituted group in the benzene ring. *Meta*- and *para*-substituted 2-(3-oxamyl) benzoic acids such as 3-methyl, 3-methoxy, 4-methyl, and 4-ethyl, afforded good to excellent yields of cyclization products (**2g**–**2l**). 3-Cl-substituted 2-(3-oxamyl) benzoic acid also produced the targeted product (**2i**) in a 46% yield. Disubstituted 2-(3-oxopentyl) benzoic acids were also compatible, giving rise to moderate to good yields (**2m**–**2u**, 44–68%). 3,4,5-Trimethoxy-2-(3-oxamyl) benzoic acid gave 68% yield of **2v**. When the benzene ring was substituted by thiophene, the yield was 42% (**2w**). Employing 3-(3-oxo-amyl)-2-naphthylformic acid and 2-(3-oxo-amyl)-2-naphthylformic acid as substrates, the cyclization products were 72% and 40%, respectively (**2x** and **2y**). 5-Methyl-2-(3-oxobutyl) benzoic acid and 5-methyl-2-(3-oxooctyl) benzoic acid generated 85% and 65% desired products, respectively (**2z** and **2aa**).

As can be seen from Figure 3, the oxidative dehydrogenation of 2-(3-oxopentyl) benzoic acids bearing different substituents produced isocoumarins in moderate to good yields. Similar to the formation of dihydroisocoumarins, donating groups at the *ortho*, *meta*, and *para*-position of carboxyl, 2,3-disubstituted 2-(3-oxo-amyl) benzoic acid, 2,4-disubstituted 2-(3-oxo-amyl) benzoic acid, 3,4-disubstituted 2-(3-oxo-amyl) benzoic acid worked well, and moderate to good yields were obtained (**3b**–**3n**). 3,4,5-Trimethoxy-2-(3-oxamyl) benzoic acid delivered 77% yield of targeted product. For heterocyclic 3-(3-oxopentyl) thiophene-2-carboxylic acid, 54% of product was observed. 76% and 51% of the isocoumarins were obtained from 3-methyl-2-(3oxobutyl) benzoic acid and 3-methyl-2-(3oxo-octyl) benzoic acid, respectively.

**Figure 4:**
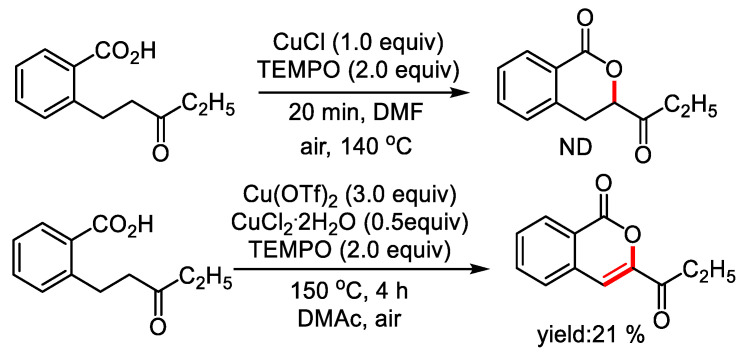
Exploration of possible free radical reactions.

To make an insight on the mechanism, free radical scavenger TEMPO was added to the reaction mixture. The addition of TEMPO depressed the formation of dihydroisocoumarin, and 21% of isocoumarin was detected, which suggesting that the reaction may undergo a radical process (Figure 4). 

**Figure 5:**
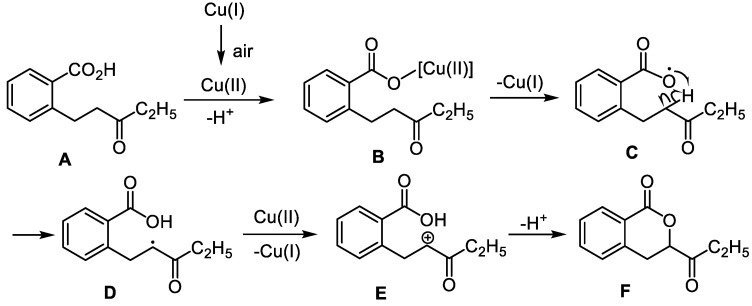
A plausible mechanism.

Based on the above results and radical studies [44,45,46], a plausible mechanism for forming dihydroisocoumarin was given in Figure 5. Firstly, Cu(I) is oxidized by air to afford Cu(II), which reacts with 2-(3-oxopentyl) benzoic acid to form the intermediate (**B**). Then the free radical intermediate (**C**) is formed via homolysis of the O–Cu bond. The carbon radical intermediate (**D**) is obtained via hydrogen transfer from α-H of carbonyl to oxygen free radical. The final product is formed via the copper-catalyzed single-electron intermediate and intramolecular cyclization (**F**).

## 5. Conclusions

In summary, the divergent syntheses of isocoumarins and 3,4-dihydroisocoumarins were achieved by intramolecular dehydrogenative cyclization of 2-(3-oxobutyl) benzoic acids via C*sp*^3^–H bonds and O–H bonds coupling in air using copper salts. The advantages of this protocol include simple operation, air atmosphere, short reaction time, broad substrate scope, and cheap copper salts. The reactions may undergo free radical process.

## Figures and Tables

**Table 1:** Selected results for optimizing reaction conditions ^a^.

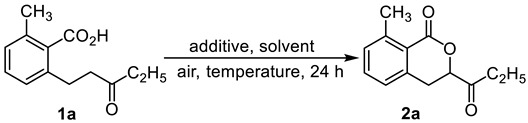				
Entry	Additive	Solvent	Conversion (%)	Yield (%) ^b^
1	CuI	DMF	80	4
2 ^c^	CuI	DMF	--	ND
3 ^d^	CuI	DMF	--	ND
4	CuCl	DMF	91	28
5	CuBr	DMF	71	18
6	CuF_2_	DMF	84	1
7	CuO	DMF	--	ND
8	Cu(OAc)_2_	DMF	--	ND
9	CuBr_2_	DMF	--	ND
10	CuCl	DMAc	93	14
11	CuCl	DMSO	82	18
12	CuCl	THF	78	4
13	CuCl	tert-pentanol	40	5
14	CuCl	toluene	--	ND
15	CuCl	1,4-dioxane	--	ND
16 ^e^	CuCl	DMF	83	35
17 ^f^	CuCl	DMF	85	61
18 ^g^	CuCl	DMF	98	49
19 ^h^	CuCl	DMF	100	53
20 ^i^	CuCl	DMF	100	50

^a^ Reaction conditions: **1a** (0.1 mmol), additive (0.5 eq.) solvent (0.6 mL), 150 °C, 24 h, air. ^b^ 1,3,5-Trimethoxybenzene was used as internal standard, and the yield was calculated by ^1^H NMR characterization of the crude product. ND means not detected. ^c^ N_2_; ^d^ O_2_; ^e^ CuCl (1.0 eq.); ^f^ CuCl (1.0 eq.), 140 °C, 20 min; ^g^ CuCl (1.0 eq.), 150 °C,10 min; ^h^ CuCl (1.0 eq.), 150 °C, 20 min; ^i^ CuCl (1.0 eq.), 150 °C, 30 min.

**Table 2:** Selected results for optimizing reaction conditions ^a^.

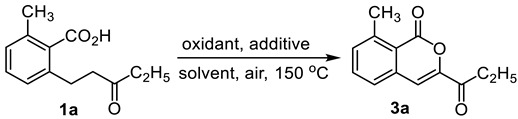					
Entry	Oxidant	Additive	Solvent	Conversion (%)	Yield (%) ^b^
1	CuO	CuCl	DMF	86	25
2 ^c^	CuO	CuCl	DMF	84	16
3 ^d^	CuO	CuCl	DMF	91	12
4	CuO	CuCl	DMAc	--	32
5	CuO	CuCl	DCE	93	10
6	CuO	CuCl	CH_3_CN		21
7	CuO	CuCl	toluene	--	ND
8	CuO	CuCl	1,4-dioxane	--	ND
9	CuO	CuCl_2_·2H_2_O	DMAc	89	33
10	CuO	Cu(OH)_2_	DMAc	100	24
11	CuO	CuCl_2_	DMAc	94	25
12	CuO	CuBr	DMAc	73	25
13	Cu(OTf)_2_	CuCl_2_·2H_2_O	DMAc	82	50
14	AgOTf	CuCl_2_·2H_2_O	DMAc	90	46
15	AgOAc	CuCl_2_·2H_2_O	DMAc	63	26
16 ^e^	Cu(OTf)_2_	CuCl_2_·2H_2_O	DMAc	87	59
17 ^f^	Cu(OTf)_2_	CuCl_2_·2H_2_O	DMAc	95	65

^a^ Reaction conditions: **1a** (0.1 mmol), oxidant (2.0 eq.), additive (1.0 eq.), solvent (0.6 mL), 150 °C, 12 h, air. ^b^ 1,3,5-Trimethoxybenzene (0.1 mmol) was used as the internal standard, and the yield was calculated by ^1^H NMR characterization of the crude product. ND means not detected. ^c^ N_2_; ^d^ O_2_; ^e^ Cu(OTf)_2_ (3.0 eq.), CuCl_2_·2H_2_O (0.5 eq.), 150 °C, 2.5 h; ^f^ **1a** (0.2 mmol), Cu(OTf)_2_ (3.0 eq.), CuCl_2_·2H_2_O (0.5 eq.), DMAc (0.6 mL), 150 °C, 4 h, air.

## Data Availability

The data presented in this study are available on request from the authors.

## Supplementary Materials

The following supporting information can be downloaded at: https://www.mdpi.com/article/10.3390/molecules28176319/s1, characterization data of compounds, the conversions of acids and NMR spectra of compounds.
