# Supplementary material for: Copper-Promoted Intramolecular Oxidative Dehydrogenation for Synthesizing Dihydroisocoumarins and Isocoumarins
Source: Molecules. 2023 Aug 29;28(17):6319. doi: 10.3390/molecules28176319 (PMC10488872; doi:10.3390/molecules28176319)
Supplement: Supplementary file 1 [file molecules-28-06319-s001.zip › molecules-2505038-supplementary.docx]

Supporting Information

Copper-Promoted Intramolecular Oxidative Dehydrogenation for

Synthesizing Dihydroisocoumarins and Isocoumarins

Qiang Zhang ^1,†^, Lin-Yan Zhang ^2,†^ and Xian-Ying Shi ^2,^*

1 Shaanxi Key Laboratory of Catalysis, School of Chemistry and Environmental Science,
Shaanxi University of Technology, Hanzhong 723001, China; zhangqiang22@snut.edu.cn

2 Key Laboratory of Syngas Conversion of Shaanxi Province, Key Laboratory for Macromolecular Science
of Shaanxi Province, School of Chemistry & Chemical Engineering, Shaanxi Normal University,
Xi’an 710062, China; azhanglinyan@hotmail.com

***** Correspondence: shixy@snnu.edu.cn

^†^ These authors contributed equally to this work.

1. Characterization data of compounds

**8-methyl-3-propionylisochroman-1-one (2a)**

10.0 mg, 46% (isolated yield). white solid, mp 95.2-96.2 ^o^C, R_f_ = 0.13 (Petroleum ether/ Ether = 8/1). ^1^H NMR (400 MHz, CDCl_3_): *δ* [ppm] = 7.39 (t, *J* = 7.6 Hz, 1H), 7.21 (d, *J* = 7.6 Hz, 1H), 7.10 (d, *J* = 7.5 Hz, 1H), 4.83 (dd, *J* = 9.7, 4.1 Hz, 1H), 3.23 (dd, *J* = 16.2, 4.0 Hz, 1H), 3.13 (dd, *J* = 16.2, 9.7 Hz, 1H), 2.80 – 2.71 (m, 2H)， 2.67 (s, 3H), 1.08 (t, *J* = 7.2 Hz, 3H). ^13^C NMR (101 MHz, CDCl_3_): *δ* [ppm] = 207.9, 163.1, 143.2, 138.4, 133.2, 131.4, 125.5, 123.3, 80.7, 32.1, 30.6, 22.1, 7.0. HRMS (ESI) m/z: calculated for C_13_H_14_O_3_, [M+Na]^+^ 241.0835; found: 241.0833.

**8-ethyl-3-propionylisochroman-1-one (2b)**

11.6 mg, 50% (isolated yield), white solid, mp 72.9-73.5 ^o^C, R_f_ = 0.27 (Petroleum ether/ Ether = 5/1). ^1^H NMR (600 MHz, CDCl_3_): *δ* [ppm] = 7.41 (t, *J* = 7.6 Hz, 1H), 7.24 (d, *J* = 7.7 Hz, 1H), 7.09 (d, *J* = 7.5 Hz, 1H), 4.82 (dd, *J* = 9.6, 4.1 Hz, 1H), 3.23 (dd, *J* = 16.2, 4.0 Hz, 1H), 3.18 – 3.04 (m, 3H), 2.80 – 2.68 (m, 2H), 1.24 (t, *J* = 7.5 Hz, 3H), 1.06 (t, *J* = 7.2 Hz, 3H). ^13^C NMR (151 MHz, CDCl_3_): *δ* [ppm] = 207.9, 162.7, 149.2, 138.4, 133.3, 130.0, 125.4, 122.7, 80.6, 32.1, 30.8, 27.7, 15.4, 6.9. HRMS (ESI) m/z: calculated for C_14_H_16_O_3_, [M+Na]^+^ 255.0992;found: 255.0993.

**8-benzyl-3-propionylisochroman-1-one (2c)**

16.8 mg, 57% (isolated yield), yellow liquid, R_f_ = 0.20 (Petroleum ether/ Ether = 5/1). ^1^H NMR (400 MHz, CDCl_3_): *δ* [ppm] = 7.41 (t, *J* = 7.6 Hz, 1H), 7.25 (t, *J* = 7.5 Hz, 2H), 7.19 – 7.12 (m, 5H), 4.80 (dd, *J* = 8.5, 4.5 Hz, 1H), 4.57 (d, *J* = 15.3 Hz, 1H), 4.44 (d, *J* = 15.3 Hz, 1H), 3.25 (dd, *J* = 16.2, 4.5 Hz, 1H), 3.17 (dd, *J* = 16.2, 8.6 Hz, 1H), 2.70 – 2.62 (m, 1H), 2.51 – 2.41 (m, 1H), 0.99 (t, *J* = 7.2 Hz, 3H). ^13^C NMR (101 MHz, CDCl_3_): *δ* [ppm] = 208.1, 162.8, 145.6, 140.4, 138.8, 133.3, 131.3, 129.2, 128.3, 126.1, 126.0, 123.1, 80.7, 39.6, 32.2, 30.7, 6.9. HRMS (ESI) m/z: calculated for C_19_H_18_O_3_, [M+Na]^+^ 317.1148;found:317.1146.

**8-phenethyl-3-propionylisochroman-1-one (2d)**

15.4 mg, 50% (isolated yield), yellow liquid, R_f_ = 0.16 (Petroleum ether/ Ether = 5/1). ^1^H NMR (600 MHz, CDCl_3_): *δ* [ppm] = 7.39 (t, *J* = 7.6 Hz, 1H), 7.28 – 7.24 (m, 4H), 7.18 (t, *J* = 8.3 Hz, 2H), 7.12 (d, *J* = 7.5 Hz, 1H), 4.79 (dd, *J* = 9.8, 4.1 Hz, 1H), 3.40 – 3.32 (m, 2H), 3.25 (dd, *J* = 16.2, 4.0 Hz, 1H), 3.15 (dd, *J* = 16.2, 9.9 Hz, 1H), 3.00 (ddd, *J* = 13.3, 9.8, 5.9 Hz, 1H), 2.87 – 2.70 (m, 3H), 1.09 (t, *J* = 7.2 Hz, 3H). ^13^C NMR (151 MHz, CDCl_3_): *δ* [ppm] = 207.8, 162.8, 146.6, 141.7, 138.6, 133.2, 131.1, 128.7, 128.2, 125.8, 123.0, 80.7, 37.7, 37.0, 32.1, 30.8, 7.0. HRMS (ESI) m/z: calculated for C_20_H_20_O_3_, [M+Na]^+^ 331.1305;found:331.1306.

**8-phenyl-3-propionylisochroman-1-one (2e)**

13.7 mg, 49% (isolated yield), white solid, mp 124.5-125.1 ^o^C, R_f_ = 0.10 (Petroleum ether/ Ether = 3/1). ^1^H NMR (600 MHz, CDCl_3_): *δ* [ppm] = 7.52 (t, *J* = 7.6 Hz, 1H), 7.41 (t, *J* = 7.2 Hz, 2H), 7.37 (t, *J* = 7.2 Hz, 1H), 7.32 (d, *J* = 6.9 Hz, 2H), 7.30 (d, *J* = 7.6 Hz, 1H), 7.25 (d, *J* = 7.5 Hz, 1H), 4.97 (dd, *J* = 8.8, 4.2 Hz, 1H), 3.36 (dd, *J* = 16.5, 4.1 Hz, 1H), 3.21 (dd, *J* = 16.5, 8.9 Hz, 1H), 2.76 (q, *J* = 7.2 Hz, 2H), 1.09 (t, *J* = 7.2 Hz, 3H). ^13^C NMR (151 MHz, CDCl_3_): *δ* [ppm] = 207.5, 162.2, 145.8, 140.8, 138.4, 132.8, 131.2, 128.3, 128.0, 127.4, 126.6, 123.2, 80.6, 32.1, 30.8, 7.0. HRMS (ESI) m/z: calculated for C_18_H_16_O_3_, [M+Na]^+^ 303.0992;found:303.0993.

**3-propionylisochroman-1-one (2f)**

15.1 mg, 74% (isolated yield), yellow liquid, R_f_ = 0.12 (Petroleum ether/ Ether = 5/1). ^1^H NMR (600 MHz, CDCl_3_): *δ* [ppm] = 8.08 (d, *J* = 7.8 Hz, 1H), 7.55 (t, *J* = 7.5 Hz, 1H), 7.40 (t, *J* = 7.6 Hz, 1H), 7.27 (d, *J* = 7.6 Hz, 1H), 4.93 (dd, *J* = 9.5, 4.5 Hz, 1H), 3.28 (dd, *J* = 16.5, 4.5 Hz, 1H), 3.18 (dd, *J* = 16.5, 9.5 Hz, 1H), 2.83 – 2.69 (m, 2H), 1.07 (t, *J* = 7.2 Hz, 3H). ^13^C NMR (151 MHz, CDCl_3_): *δ* [ppm] = 207.6, 163.8, 137.3, 134.2, 130.4, 128.0, 127.6, 124.7, 81.2, 32.2, 29.3, 6.9. HRMS (ESI) m/z: calculated for C_12_H_12_O_3_, [M+Na]^+^ 227.0679;found: 227.0679.

**7-methyl-3-propionylisochroman-1-one (2g)**

17.1 mg, 78% (isolated yield), white solid, mp 83.4-84.2 ^o^C, R_f_ = 0.13 (Petroleum ether/ Ether = 5/1). ^1^H NMR (400 MHz, CDCl_3_): *δ* [ppm] = 7.89 (s, 1H), 7.35 (d, *J* = 7.7 Hz, 1H), 7.15 (d, *J* = 7.7 Hz, 1H), 4.91 (dd, *J* = 9.2, 4.6 Hz, 1H), 3.24 (dd, *J* = 16.5, 4.5 Hz, 1H), 3.13 (dd, *J* = 16.5, 9.3 Hz, 1H)., 2.82 – 2.66 (m, 2H), 2.37 (s, 3H), 1.06 (t, *J* = 7.2 Hz, 3H). ^13^C NMR (101 MHz, CDCl_3_): *δ* [ppm] =207.8, 164.1, 138.0, 135.1, 134.3, 130.5, 127.5, 124.4, 81.3, 32.2, 29.0, 20.9, 6.9. HRMS (ESI) m/z: calculated for C_13_H_14_O_3_, [M+Na]^+^ 241.0835; found: 241.0833.

**7-methoxy-3-propionylisochroman-1-one (2h)**

16.9 mg, 72% (isolated yield), white solid, mp 89.3-90.1 ^o^C, R_f_ = (Petroleum ether/ Ether = 5/1). ^1^H NMR (600 MHz, CDCl_3_): *δ* [ppm] = 7.70 (d, *J* = 7.8 Hz, 1H), 7.35 (t, *J* = 8.0 Hz, 1H), 7.08 (d, *J* = 8.1 Hz, 1H), 4.91 (dd, *J* = 9.5, 4.7 Hz, 1H), 3.87 (s, 3H), 3.37 (dd, *J* = 17.2, 4.7 Hz, 1H), 3.03 (dd, *J* = 17.2, 9.5 Hz, 1H), 2.82 – 2.68 (m, 2H), 1.09 (t, *J* = 7.2 Hz, 3H). ^13^C NMR (151 MHz, CDCl_3_): *δ* [ppm] = 207.6, 163.9, 155.8, 128.3, 126.2, 125.6, 121.8, 115.2, 81.1, 55.8, 32.1, 23.3, 7.0. HRMS (ESI) m/z: calculated for C_13_H_14_O_4_, [M+Na]^+^ 257.0784; found: 257.0784.

**7-chloro-3-propionylisochroman-1-one (2i)**

10.9 mg, 46% (isolated yield), yellow liquid, R_f_ = 0.27 (Petroleum ether/ Ether = 5/1). ^1^H NMR (600 MHz, CDCl_3_): *δ* [ppm] = 8.04 (d, *J* = 7.8 Hz, 1H), 7.62 (d, *J* = 8.0 Hz, 1H), 7.36 (t, *J* = 7.9 Hz, 1H), 4.97 (dd, *J* = 9.3, 4.7 Hz, 1H), 3.48 (dd, *J* = 17.3, 4.6 Hz, 1H), 3.19 (dd, *J* = 17.3, 9.4 Hz, 1H), 2.84 – 2.68 (m, 2H), 1.10 (t, *J* = 7.2 Hz, 3H). ^13^C NMR (151 MHz, CDCl_3_): *δ* [ppm] = 206.8, 162.9, 135.3, 134.6, 133.1, 128.9, 128.7, 126.6, 80.5, 32.1, 26.9, 7.0. HRMS (ESI) m/z: calculated for C_12_H_11_ClO_3_, [M+Na]^+^ 261.0289;found: 261.0289.

**6-methyl-3-propionylisochroman-1-one (2j)**

17.9 mg, 82% (isolated yield), white solid, mp 62.7-63.3 ^o^C, R_f_ = 0.13 (Petroleum ether/ Ether = 5/1). ^1^H NMR (400 MHz, CDCl_3_): *δ* [ppm] = 7.94 (d, *J* = 8.0 Hz, 1H), 7.18 (d, *J* = 7.9 Hz, 1H), 7.05 (s, 1H), 4.89 (dd, *J* = 9.1, 4.7 Hz, 1H), 3.22 (dd, *J* = 16.5, 4.7 Hz, 1H), 3.13 (dd, *J* = 16.5, 9.2 Hz, 1H), 2.83 – 2.64 (m, 2H), 2.38 (s, 3H), 1.05 (t, *J* = 7.2 Hz, 3H). ^13^C NMR (101 MHz, CDCl_3_): *δ* [ppm] = 207.8, 163.9, 145.3, 137.3, 130.3, 128.9, 128.1, 121.9, 81.2, 32.2, 29.3, 21.7, 6.9. HRMS (ESI) m/z: calculated for C_13_H_14_O_3_, [M+Na]^+^ 241.0835;found: 241.0836.

**6-ethyl-3-propionylisochroman-1-one (2k)**

18.8 mg, 81% (isolated yield), white solid, mp 62.4-62.7 ^o^C, R_f_ = 0.14 (Petroleum ether/ Ether = 5/1). ^1^H NMR (600 MHz, CDCl_3_): *δ* [ppm] = 7.98 (d, *J* = 8.0 Hz, 1H), 7.22 (d, *J* = 7.9 Hz, 1H), 7.08 (s, 1H), 4.90 (dd, *J* = 9.4, 4.5 Hz, 1H), 3.24 (dd, J = 16.5, 4.5 Hz, 1H), 3.15 (dd, J = 16.4, 9.5 Hz, 1H), 2.83 – 2.76 (m, 1H), 2.75 – 2.65 (m, 3H), 1.24 (t, *J* = 7.6 Hz, 3H), 1.07 (t, *J* = 7.2 Hz, 3H). ^13^C NMR (101 MHz, CDCl_3_): *δ* [ppm] = 207.9, 163.9, 151.5, 137.5, 130.5, 127.8, 126.9, 122.1, 81.2, 32.2, 29.4, 29.0, 14.9, 6.9. HRMS (ESI) m/z: calculated for C_14_H_16_O_3_, [M+Na]^+^ 255.0992; found: 255.0995.

**6-methoxy-3-propionylisochroman-1-one (2l)**

15.2 mg, 65% (isolated yield), white solid, mp 75.5-76.1 ^o^C, R_f_ = 0.12 (Petroleum ether/ Ether = 3/1). ^1^H NMR (600 MHz, CDCl_3_): *δ* [ppm] = 8.01 (d, *J* = 8.7 Hz, 1H), 6.88 (dd, *J* = 8.7, 2.1 Hz, 1H), 6.71 (s, 1H), 4.89 (dd, *J* = 9.2, 4.6 Hz, 1H), 3.85 (s, 3H), 3.22 (dd, J = 16.5, 4.6 Hz, 1H), 3.15 (dd, J = 16.5, 9.2 Hz, 1H), 2.83 – 2.75 (m, 1H), 2.74 – 2.67 (m, 1H), 1.06 (t, *J* = 7.2 Hz, 3H). ^13^C NMR (151 MHz, CDCl_3_): *δ* [ppm] = 208.0, 164.2, 163.7, 139.8, 132.7, 117.1, 114.1, 112.2, 81.1, 55.6, 32.2, 29.6, 6.9. HRMS (ESI) m/z: calculated for C_14_H_14_O_3_, [M+Na]^+^ 255.0992;found: 255.0993.

**6-chloro-8-methyl-3-propionylisochroman-1-one (2m)**

11.1 mg, 44% (isolated yield), yellow solid, mp 81.1-81.6 ^o^C, R_f_ = 0.18 (Petroleum ether/ Ether = 5/1). ^1^H NMR (600 MHz, CDCl_3_): *δ* [ppm] = 7.20 (s, 1H), 7.10 (s, 1H), 4.82 (dd, *J* = 9.5, 4.1 Hz, 1H), 3.22 (dd, *J* = 16.3, 4.0 Hz, 1H), 3.12 (dd, *J* = 16.3, 9.6 Hz, 1H), 2.80 – 2.68 (m, 2H), 2.64 (s, 3H), 1.07 (t, *J* = 7.2 Hz, 3H). ^13^C NMR (151 MHz, CDCl_3_): *δ* [ppm] = 207.4, 162.4, 145.3, 140.1, 139.2, 131.5, 125.6, 121.8, 80.4, 32.2, 30.4, 22.1, 7.0. HRMS (ESI) m/z: calculated for C_13_H_13_ClO_3_, [M+Na]^+^ 275.0445;found:275.0443.

**6,8-dimethyl-3-propionylisochroman-1-one (2n)**

12.1 mg, 52% (isolated yield), white solid, mp 84.4-84.9 ^o^C, R_f_ = 0.18 (Petroleum ether/ Ether = 5/1). ^1^H NMR (600 MHz, CDCl_3_): *δ* [ppm] = 7.01 (s, 1H), 6.89 (s, 1H), 4.80 (dd, *J* = 9.6, 4.2 Hz, 1H), 3.17 (dd, *J* = 16.2, 4.2 Hz, 1H), 3.09 (dd, *J* = 16.2, 9.6 Hz, 1H), 2.82 – 2.66 (m, 2H), 2.62 (s, 3H), 2.33 (s, 3H), 1.06 (t, *J* = 7.2 Hz, 3H). ^13^C NMR (151 MHz, CDCl_3_): *δ* [ppm] = 208.1, 163.2, 144.1, 143.2, 138.5, 132.3, 126.1, 120.5, 80.7, 32.1, 30.5, 22.0, 21.4, 6.9. HRMS (ESI) m/z: calculated for C_14_H_16_O_3_, [M+Na]^+^ 255.0992;found: 255.0993.

**6-fluoro-8-**methyl**-3-propionylisochroman-1-one (2o)**

11.8 mg, 50% (isolated yield), white solid, mp 89.0-89.3 ^o^C, R_f_ = 0.15 (Petroleum ether/ Ether = 5/1). ^1^H NMR (400 MHz, CDCl_3_): *δ* [ppm] = 6.90 (d, *J* = 9.5 Hz, 1H), 6.80 (d, *J* = 8.1 Hz, 1H), 4.82 (dd, *J* = 9.5, 4.3 Hz, 1H), 3.23 (dd, *J* = 16.4, 4.2 Hz, 1H), 3.13 (dd, *J* = 16.4, 9.5 Hz, 1H), 2.81 – 2.69 (m, 2H), 2.66 (s, 3H), 1.07 (t, *J* = 7.2 Hz, 3H).^13^C NMR (101 MHz, CDCl_3_): *δ* [ppm] = 207.5, 164.6 (d, *J* = 256.7 Hz), 162.3, 147.2 (d, *J* = 9.7 Hz), 141.5 (d, *J* = 9.7 Hz), 119.7 (d, *J* = 2.9 Hz), 118.4 (d, *J* = 21.3 Hz), 112.5 (d, *J* = 21.9 Hz), 80.3, 32.2, 30.6, 22.4, 6.9. HRMS (ESI) m/z: calculated for C_13_H_13_FO_3_, [M+Na]^+^ 259.0741;found: 259.0738.

**6,7-dimethyl-3-propionylisochroman-1-one (2p)**

15.8 mg, 68% (isolated yield), yellow solid, mp 91.1-91.5 ^o^C, R_f_ = 0.13 (Petroleum ether/ Ether = 5/1). ^1^H NMR (600 MHz, CDCl_3_): *δ* [ppm] = 7.83 (s, 1H), 7.01 (s, 1H), 4.89 (dd, *J* = 9.2, 4.6 Hz, 1H), 3.19 (dd, *J* = 16.4, 4.6 Hz, 1H), 3.11 (dd, *J* = 16.4, 9.2 Hz, 1H), 2.81 – 2.75 (m, 1H), 2.72 – 2.67 (m, 1H), 2.29 (s, 3H), 2.27 (s, 3H), 1.06 (t, *J* = 7.2 Hz, 3H). ^13^C NMR (151 MHz, CDCl_3_): *δ* [ppm] = 208.1, 164.2, 144.3, 136.8, 134.9, 131.0, 128.7, 122.1, 81.5, 32.3, 28.9, 20.1, 19.4, 7.0. HRMS (ESI) m/z: calculated for C_14_H_16_O_3_, [M+Na]^+^ 255.0992; found: 255.0993 .

**6,7-dimethoxy-3-propionylisochroman-1-one (2q)**

17.4 mg, 66% (isolated yield), white solid, mp 160.1-160.7 ^o^C, R_f_ = 0.03 (Petroleum ether/ Ether = 3/1). ^1^H NMR (600 MHz, CDCl_3_): *δ* [ppm] = 7.51 (s, 1H), 6.67 (s, 1H), 4.90 (dd, *J* = 9.0, 4.8 Hz, 1H), 3.93 (s, 3H), 3.90 (s, 3H), 3.20 (dd, *J* = 16.4, 4.8 Hz, 1H), 3.14 (dd, *J* = 16.4, 9.1 Hz, 1H), 2.84 – 2.77 (m, 1H), 2.74 – 2.67 (m, 1H), 1.07 (t, *J* = 7.2 Hz, 3H). ^13^C NMR (151 MHz, CDCl_3_): *δ* [ppm] = 208.2, 163.8, 154.1, 148.8, 131.9, 116.7, 111.7, 109.4, 81.4, 56.2, 56.1, 32.3, 28.9, 6.9. HRMS (ESI) m/z: calculated for C_14_H_16_O_5_, [M+Na]^+^ 287.0890;found: 287.0890.

**7-chloro-6-methyl-3-propionylisochroman-1-one (2r)**

14.9 mg, 59% (isolated yield), white solid, mp 99.9-100.4 ^o^C, R_f_ = 0.17 (Petroleum ether/ Ether = 3/1). ^1^H NMR (600 MHz, CDCl_3_): *δ* [ppm] = 8.05 (s, 1H), 7.15 (s, 1H), 4.92 (dd, *J* = 9.1, 4.6 Hz, 1H), 3.23 (dd, *J* = 16.6, 4.6 Hz, 1H), 3.13 (dd, *J* = 16.6, 9.1 Hz, 1H), 2.81 – 2.67 (m, 2H), 2.42 (s, 3H), 1.08 (t, *J* = 7.2 Hz, 3H). ^13^C NMR (151 MHz, CDCl_3_): *δ* [ppm] = 207.4, 162.8, 143.3, 135.5, 134.3, 130.5, 130.0, 123.7, 81.2, 32.2, 28.7, 20.4, 6.9. HRMS (ESI) m/z: calculated for C_13_H_13_ClO_3_, [M+Na]^+^ 275.0445;found: 275.0445.

**5,7-dimethyl-3-propionylisochroman-1-one (2s)**

16.7 mg, 72% (isolated yield), yellow solid, mp 115.1-115.5 ^o^C, R_f_ = 0.15 (Petroleum ether/ Ether = 5/1). ^1^H NMR (600 MHz, CDCl_3_): *δ* [ppm] = 7.76 (s, 1H), 7.23 (s, 1H), 4.87 (dd, *J* = 9.6, 4.5 Hz, 1H), 3.19 (dd, *J* = 16.7, 4.5 Hz, 1H), 3.01 (dd, *J* = 16.7, 9.7 Hz, 1H), 2.84 – 2.69 (m, 2H), 2.33 (s, 3H), 2.28 (s, 3H), 1.07 (t, *J* = 7.2 Hz, 3H). ^13^C NMR (151 MHz, CDCl_3_): *δ* [ppm] = 208.2, 164.4, 137.3, 136.6, 135.4, 133.0, 128.4, 124.4, 80.9, 32.2, 26.2, 20.8, 18.7, 7.0. HRMS (ESI) m/z: calculated for C_14_H_16_O_3_, [M+Na]^+^ 255.0992;found: 255.0993.

**7-methoxy-8-methyl-3-propionylisochroman-1-one (2t)**

10.9 mg, 44% (isolated yield), white solid, mp 84.3-85.0 ^o^C, R_f_ = 0.16 (Petroleum ether/ Ether = 5/1). ^1^H NMR (600 MHz, CDCl_3_): *δ* [ppm] = 7.05 (d, *J* = 8.3 Hz, 1H), 7.00 (d, *J* = 8.3 Hz, 1H), 4.79 (dd, *J* = 9.6, 4.0 Hz, 1H), 3.83 (s, 3H), 3.17 (dd, *J* = 16.0, 4.0 Hz, 1H), 3.06 (dd, *J* = 15.9, 9.6 Hz, 1H), 2.77 – 2.68 (m, 2H), 2.54 (s, 3H), 1.06 (t, *J* = 7.2 Hz, 3H). ^13^C NMR (151 MHz, CDCl_3_): *δ* [ppm] = 208.0, 163.23, 157.6, 131.9, 129.5, 125.3, 124.4, 115.1, 81.1, 55.9, 32.1, 30.3, 13.0, 6.9. HRMS (ESI) m/z: calculated for C_14_H_16_O_4_, [M+Na]^+^ 271.0941;found: 271.0938.

**7,8-dimethyl-3-propionylisochroman-1-one (2u)**

9.2 mg, 42% (isolated yield), white solid, mp 76.7-77.3 ^o^C, R_f_ = 0.19 (Petroleum ether/ Ether = 5/1). ^1^H NMR (600 MHz, CDCl_3_): *δ* [ppm] = 7.28 (d, *J* = 7.7 Hz, 1H), 6.98 (d, *J* = 7.6 Hz, 1H), 4.79 (dd, *J* = 9.7, 4.0 Hz, 1H), 3.18 (dd, *J* = 16.1, 4.0 Hz, 1H), 3.08 (dd, *J* = 16.1, 9.7 Hz, 1H), 2.80 – 2.68 (m, 2H), 2.57 (s, 3H), 2.31 (s, 3H), 1.06 (t, *J* = 7.2 Hz, 3H). ^13^C NMR (151 MHz, CDCl_3_): *δ* [ppm] = 207.9, 163.5, 141.3, 137.8, 135.8, 134.7, 124.6, 123.8, 80.8, 32.1, 30.7, 20.6, 17.1, 6.9. HRMS (ESI) m/z: calculated for C_14_H_16_O_3_, [M+Na]^+^ 255.0992;found:255.0991.

**5,6,7-trimethoxy-3-propionylisochroman-1-one (2v)**

20.0 mg, 68% (isolated yield), yellow solid, mp 87.5-88.3 ^o^C, R_f_ = 0.13 (Petroleum ether/ Ether = 3/1). ^1^H NMR (600 MHz, CDCl_3_): *δ* [ppm] = 7.39 (s, 1H), 4.89 (dd, *J* = 9.1, 4.7 Hz, 1H), 3.93 (s, 3H), 3.88 (s, 3H), 3.86 (s, 3H), 3.29 (dd, *J* = 16.9, 4.7 Hz, 1H), 3.03 (dd, *J* = 16.9, 9.1 Hz, 1H), 2.79 – 2.67 (m, 2H), 1.07 (t, *J* = 7.2 Hz, 3H). ^13^C NMR (151 MHz, CDCl_3_): *δ* [ppm] = 207.6, 163.6, 153.0, 149.6, 147.3, 124.6, 119.4, 108.5, 81.4, 61.0, 60.9, 56.2, 32.1, 23.4, 7.0. HRMS (ESI) m/z: calculated for C_15_H_18_O_6_, [M+Na]^+^ 317.0996;found: 317.0995.

**5-propionyl-4,5-dihydro-7H-thieno[2,3-c]pyran-7-one (2w)**

8.8 mg, 42% (isolated yield), white solid, mp 66.8-67.3 ^o^C, R_f_ = 0.18 (Petroleum ether/ Ether = 3/1). ^1^H NMR (600 MHz, CDCl_3_): *δ* [ppm] = 7.69 (d, *J* = 5.0 Hz, 1H), 7.00 (d, *J* = 5.0 Hz, 1H), 4.99 (dd, *J* = 9.3, 5.1 Hz, 1H), 3.28 (dd, *J* = 17.0, 5.1 Hz, 1H), 3.17 (dd, *J* = 17.0, 9.3 Hz, 1H), 2.93 – 2.64 (m, 2H), 1.09 (t, *J* = 7.2 Hz, 3H). ^13^C NMR (151 MHz, CDCl_3_): *δ* [ppm] = 207.7, 159.6, 145.9, 135.4, 126.8, 126.2, 82.5, 32.4, 26.6, 7.0. HRMS (ESI) m/z: calculated for C_10_H_10_O_3_S, [M+Na]^+^ 233.0243;found:233.0241.

**3-propionyl-3,4-dihydro-1H-benzo[g]isochromen-1-one (2x)**

19.0 mg, 75% (isolated yield), white solid, mp 140.2-140.7 ^o^C, R_f_ = 0.19 (Petroleum ether/ Ether = 3/1). ^1^H NMR (600 MHz, CDCl_3_): *δ* [ppm] = 8.71 (s, 1H), 7.96 (d, *J* = 8.2 Hz, 1H), 7.83 (d, *J* = 8.2 Hz, 1H), 7.70 (s, 1H), 7.62 (t, J = 7.5 Hz, 1H), 7.54 (t, J = 7.5 Hz, 1H), 5.02 (dd, *J* = 9.1, 4.3 Hz, 1H), 3.48 (dd, *J* = 16.2, 4.2 Hz, 1H), 3.35 (dd, *J* = 16.2, 9.1 Hz, 1H), 2.86 – 2.71 (m, 2H), 1.09 (t, *J* = 7.2 Hz, 3H). ^13^C NMR (151 MHz, CDCl_3_): *δ* [ppm] = 207.6, 164.2, 135.8, 132.6, 132.2, 131.7, 129.5, 129.3, 127.3, 126.8, 126.2, 122.3, 81.5, 32.2, 29.9, 7.0. HRMS (ESI) m/z: calculated for C_16_H_14_O_3_, [M+Na]^+^ 277.0835;found:277.0835.

**3-propionyl-3,4-dihydro-1H-benzo[h]isochromen-1-one (2y)**

10.2 mg, 40% (isolated yield), white solid, mp 96.0-96.4 ^o^C, R_f_ = 0.20 (Petroleum ether/ Ether = 3/1). ^1^H NMR (600 MHz, CDCl_3_): *δ* [ppm] = 9.19 (d, *J* = 8.7 Hz, 1H), 8.03 (d, *J* = 8.3 Hz, 1H), 7.87 (d, *J* = 8.1 Hz, 1H), 7.69 (t, *J* = 7.7 Hz, 1H), 7.56 (t, *J* = 7.5 Hz, 1H), 7.35 (d, *J* = 8.3 Hz, 1H), 4.93 (dd, *J* = 9.4, 4.7 Hz, 1H), 3.40 (dd, *J* = 16.7, 4.7 Hz, 1H), 3.35 (dd, *J* = 16.6, 9.4 Hz, 1H), 2.96 – 2.70 (m, 2H), 1.10 (t, *J* = 7.2 Hz, 3H). ^13^C NMR (151 MHz, CDCl_3_): *δ* [ppm] = 207.9, 162.9, 139.4, 135.3, 133.3, 131.7, 129.1, 128.7, 126.5, 126.0, 124.9, 119.7, 80.2, 32.3, 30.9, 6.9. HRMS (ESI) m/z: calculated for C_16_H_14_O_3_, [M+Na]^+^ 277.0835;found:277.0835.

**3-acetyl-7-methylisochroman-1-one (2z)**

17.3 mg, 85% (isolated yield), yellow solid, mp 93.1-93.6 ^o^C, R_f_ = 0.09 (Petroleum ether/ Ether = 5/1). ^1^H NMR (600 MHz, CDCl_3_): *δ* [ppm] = 7.88 (s, 1H), 7.35 (d, *J* = 7.5 Hz, 1H), 7.15 (d, *J* = 7.7 Hz, 1H), 4.89 (dd, *J* = 9.3, 4.6 Hz, 1H), 3.23 (dd, *J* = 16.4, 4.5 Hz, 1H), 3.14 (dd, *J* = 16.4, 9.3 Hz, 1H), 2.37 (s, 3H), 2.35 (s, 3H). ^13^C NMR (151 MHz, CDCl_3_): *δ* [ppm] = 205.1, 164.0, 138.0, 135.1, 134.1, 130.5, 127.5, 124.3, 81.5, 28.7, 26.5, 20.9. HRMS (ESI) m/z: calculated for C_12_H_12_O_3_, [M+Na]^+^ 227.0679;found:227.0680.

**3-hexanoyl-7-methylisochroman-1-one (2aa)**

17.2 mg, 65% (isolated yield), white solid, mp 48.5-49.1 ^o^C, R_f_ = 0.328 (Petroleum ether/ Ether = 3/1). ^1^H NMR (600 MHz, CDCl_3_): *δ* [ppm] = 7.89 (s, 1H), 7.35 (d, *J* = 7.8 Hz, 1H),, 7.15 (d, *J* = 7.7 Hz, 1H), 4.89 (dd, *J* = 9.4, 4.6 Hz, 1H), 3.23 (dd, *J* = 16.4, 4.5 Hz, 1H), 3.13 (dd, *J* = 16.4, 9.4 Hz, 1H), 2.82 – 2.62 (m, 2H), 2.37 (s, 3H), 1.60 – 1.55 (m, 2H), 1.32 – 1.21 (m, 4H), 0.86 (t, *J* = 7.1 Hz, 3H). ^13^C NMR (151 MHz, CDCl_3_): *δ* [ppm] = 207.3, 164.1, 138.0, 135.1, 134.3, 130.5, 127.5, 124.4, 81.4, 38.7, 31.1, 28.9, 22.5, 22.3, 20.9, 13.8. HRMS (ESI) m/z: calculated for C_16_H_20_O_3_, [M+Na]^+^ 283.1305;found:283.1306.

**8-methyl-3-propionyl-1H-isochromen-1-one (3a)**

21.4 mg, 50% (isolated yield), white solid, mp 119.1-120.7 ^o^C, R_f_ = 0.42 (Petroleum ether/ Ether = 5/1). ^1^H NMR (600 MHz, CDCl_3_): *δ* [ppm] = 7.61 (t, *J* = 7.6 Hz, 1H), 7.43 (t, *J* = 7.9 Hz, 2H), 7.29 (s, 1H), 2.96 (q, *J* = 7.2 Hz, 2H), 2.84 (s, 3H), 1.19 (t, *J* = 7.2 Hz, 3H). ^13^C NMR (151 MHz, CDCl_3_): *δ* [ppm] = 195.1, 160.2, 149.1, 144.1, 136.7, 134.3, 133.5, 126.5, 121.2, 109.0, 31.6, 23.1, 7.3. HRMS (ESI) m/z: calculated for C_13_H_12_O_3_, [M+Na]^+^ 239.0679;found:239.0670.

**8-ethyl-3-propionyl-1H-isochromen-1-one (3b)**

22.1 mg, 48% (isolated yield), white solid, mp 109.7-110.3 ^o^C, R_f_ = 0.39 (Petroleum ether/ Ether = 5/1). ^1^H NMR (400 MHz, CDCl_3_): *δ* [ppm] = 7.66 (t, *J* = 7.7 Hz, 1H), 7.46 (dd, *J* = 7.6, 2.3 Hz, 2H), 7.31 (s, 1H), 3.30 (q, *J* = 7.4 Hz, 2H), 2.98 (q, *J* = 7.2 Hz, 2H), 1.29 (t, *J* = 7.4 Hz, 3H), 1.20 (t, *J* = 7.2 Hz, 3H). ^13^C NMR (151 MHz, CDCl_3_): *δ* [ppm] = 195.0, 159.6, 150.1, 148.9, 136.9, 134.5, 132.2, 126.6, 120.4, 109.0, 31.6, 28.5, 15.3, 7.3. HRMS (ESI) m/z: calculated for C_14_H_14_O_3_, [M+Na]^+^ 253.0835;found:253.0825.

**8-benzyl-3-propionyl-1H-isochromen-1-one (3c)**

29.4 mg, 50% (isolated yield), yellow liquid, mp 115.3-115.9 ^o^C, R_f_ = 0.38 (Petroleum ether/ Ether = 5/1). ^1^H NMR (400 MHz, CDCl_3_): *δ* [ppm] = 7.66 (t, *J* = 7.7 Hz, 1H), 7.49 (d, *J* = 7.6 Hz, 1H), 7.38 (d, *J* = 7.6 Hz, 1H), 7.32 – 7.26(m, 3H), 7.22 – 7.20(m, 3H), 4.69 (s, 2H), 2.95 (q, *J* = 7.2 Hz, 2H), 1.19 (t, *J* = 7.2 Hz, 3H). ^13^C NMR (101 MHz, CDCl_3_): *δ* [ppm] = 195.0, 159.8, 149.03, 146.4, 139.9, 137.1, 134.4, 133.5, 129.2, 128.4, 127.1, 126.2, 120.7, 108.9, 40.3, 31.6, 7.3. HRMS (ESI) m/z: calculated for C_19_H_16_O_3_, [M+Na]^+^ 315.0992;found:315.0993.

**8-phenethyl-3-propionyl-1H-isochromen-1-one (3d)**

30.6 mg, 50% (isolated yield), yellow liquid, mp 116.3-116.8 ^o^C, R_f_ = 0.41 (Petroleum ether/ Ether = 5/1). ^1^H NMR (600 MHz, CDCl_3_): *δ* [ppm] = 7.63 (t, *J* = 7.6 Hz, 1H), 7.49 (d, *J* = 7.7 Hz, 1H), 7.37 – 7.34 (m, 2H), 7.30 – 7.29(m, 4H), 7.22 – 7.18 (m, 1H), 3.55 – 3.52 (m, 2H), 3.00 (q, *J* = 7.2 Hz, 2H), 2.95 – 2.92 (m, 2H), 1.22 (t, *J* = 7.2 Hz, 3H). ^13^C NMR (151 MHz, CDCl_3_): *δ* [ppm] = 195.0, 159.7, 149.0, 147.5, 141.6, 137.1, 134.4, 133.40 128.6, 128.3, 127.0, 125.9, 120.6, 109.0, 38.0, 37.6, 31.6, 7.3. HRMS (ESI) m/z: calculated for C_20_H_18_O_3_, [M+Na]^+^ 329.1148;found:329.1149.

**8-phenyl-3-propionyl-1H-isochromen-1-one (3e)**

29.3 mg, 53% (isolated yield), white solid, mp 166.8-167.4 ^o^C, R_f_ = 0.29 (Petroleum ether/ Ether = 5/1). ^1^H NMR (400 MHz, CDCl_3_): *δ* [ppm] = 7.77 (t, *J* = 7.6 Hz, 1H), 7.63 (d, *J* = 7.7 Hz, 1H), 7.49 (d, *J* = 7.5 Hz, 1H), 7.43 – 7.40 (m, 4H), 7.33 – 7.31 (m, 2H), 2.95 (q, *J* = 7.0 Hz, 2H), 1.19 (t, *J* = 7.1 Hz, 3H). ^13^C NMR (151 MHz, CDCl_3_): *δ* [ppm] = 195.2, 159.0, 149.4, 146.5, 140.7, 136.7, 134.0, 133.7, 128.3, 127.9, 127.8, 127.5, 119.9, 108.5, 31.6, 7.3. HRMS (ESI) m/z: calculated for C_18_H_14_O_3_, [M+Na]^+^ 301.0835;found:301.0820.

**3-propionyl-1H-isochromen-1-one (3f)**

28.4 mg, 70% (isolated yield), yellow liquid, mp 107.4-108.1 ^o^C, R_f_ = 0.28 (Petroleum ether/ Ether = 5/1). ^1^H NMR (400 MHz, CDCl_3_): *δ* [ppm] = 8.35 (d, *J* = 7.8 Hz, 1H), 7.80 (t, *J* = 7.5 Hz, 1H), 7.67 – 7.63 (m, 2H), 7.39 (s, 1H), 2.99 (q, *J* = 7.2 Hz, 2H), 1.21 (t, *J* = 7.2 Hz, 3H). ^13^C NMR (151 MHz, CDCl_3_): *δ* [ppm] = 195.0, 160.9, 149.4, 135.2, 130.6, 130.0, 128.20, 122.9, 108.4, 31.7, 7.3. HRMS (ESI) m/z: calculated for C_12_H_10_O_3_, [M+Na]^+^ 225.0522;found:225.0521.

**7-methyl-3-propionyl-1H-isochromen-1-one (3g)**

25.9 mg, 60% (isolated yield), white solid, mp 119.4-120.2 ^o^C, R_f_ = 0.31 (Petroleum ether/ Ether = 5/1). ^1^H NMR (600 MHz, CDCl_3_): *δ* [ppm] = 8.14 (s, 1H), 7.60 (d, *J* = 7.8 Hz, 1H), 7.52 (d, *J* = 7.9 Hz, 1H), 7.36 (s, 1H), 2.96 (q, *J* = 7.2 Hz, 2H), 2.50 (s, 3H), 1.19 (t, *J* = 7.2 Hz, 3H). ^13^C NMR (151 MHz, CDCl_3_): *δ* [ppm] = 195.0, 161.0, 148.8, 141.5, 136.4, 132.6, 129.8, 128.1, 122.76, 108.6, 31.6, 21.6, 7.3. HRMS (ESI) m/z: calculated for C_13_H_12_O_3_, [M+Na]^+^ 239.0679;found:239.0677.

**6-methyl-3-propionyl-1H-isochromen-1-one (3h)**

33.2 mg, 77% (isolated yield), white solid, mp 143.9-144.3 ^o^C, R_f_ = 0.37 (Petroleum ether/ Ether = 5/1). ^1^H NMR (400 MHz, CDCl_3_): *δ* [ppm] = 8.21 (d, *J* = 8.0 Hz, 1H), 7.45 (d, *J* = 8.0 Hz, 1H), 7.40 (s, 1H), 7.32 (s, 1H), 2.97 (dd, *J* = 13.2, 6.3 Hz, 2H), 2.50 (s, 3H), 1.19 (t, *J* = 6.7 Hz, 3H). ^13^C NMR (101 MHz, CDCl_3_): *δ* [ppm] = 195.1, 160.9, 149.5, 146.5, 135.2, 131.9, 129.9, 128.2, 120.4, 108.5, 31.7, 21.9, 7.3. HRMS (ESI) m/z: calculated for C_13_H_12_O_3_, [M+Na]^+^ 239.0679;found:239.0680.

**6-ethyl-3-propionyl-1H-isochromen-1-one (3i)**

35.7 mg, 78% (isolated yield), white solid, mp 97.6-98.1 ^o^C, R_f_ = 0.27 (Petroleum ether/ Ether = 5/ 1). ^1^H NMR (600 MHz, CDCl_3_): *δ* [ppm] = 8.24 (d, *J* = 8.1 Hz, 1H), 7.48 (d, *J* = 8.1 Hz, 1H), 7.42 (s, 1H), 7.34 (s, 1H), 2.98 (q, *J* = 7.2 Hz, 2H), 2.79 (q, *J* = 7.6 Hz, 2H), 1.30 (t, *J* = 7.6 Hz, 3H), 1.19 (t, *J* = 7.2 Hz, 3H). ^13^C NMR (101 MHz, CDCl_3_): *δ* [ppm] = 195.1, 161.0, 152.5, 149.5, 135.4, 130.9, 130.1, 127.0, 120.6, 108.6, 31.7, 29.1, 14.9, 7.3. HRMS (ESI) m/z: calculated for C_14_H_14_O_3_, [M+Na]^+^ 253.0835;found:253.0834.

**6-chloro-8-methyl-3-propionyl-1H-isochromen-1-one (3j)**

24.5 mg, 49% (isolated yield), yellow solid, mp 154.7-155.5 ^o^C, R_f_ = 0.18 (Petroleum ether/ Ether = 5/1). ^1^H NMR (600 MHz, CDCl_3_): *δ* [ppm] = 7.42 (s, 1H), 7.40 (s, 1H), 7.21 (s, 1H), 2.97 (q, *J* = 7.2 Hz, 2H), 2.82 (s, 3H), 1.20 (t, *J* = 7.2 Hz, 3H). ^13^C NMR (151 MHz, CDCl_3_): *δ* [ppm] = 194.9, 159.6, 149.9, 146.2, 140.7, 138.1, 133.3, 125.8, 119.5, 107.8, 31.7, 23.1, 7.2. HRMS (ESI) m/z: calculated for C_13_H_11_ClO_3_, [M+Na]^+^ 273.0279;found:273.0285.

**6,8-dimethyl-3-propionyl-1H-isochromen-1-one (3k)**

28.5 mg, 62% (isolated yield), white solid, mp 113.4-114.1 ^o^C, R_f_ = 0.44 (Petroleum ether/ Ether = 5/1). ^1^H NMR (600 MHz, CDCl_3_): *δ* [ppm] = 7.23 (s, 2H), 7.21 (s, 1H), 2.95 (q, *J* = 7.2 Hz, 2H), 2.79 (s, 3H), 2.43 (s, 3H), 1.18 (t, *J* = 7.2 Hz, 3H). ^13^C NMR (151 MHz, CDCl_3_): *δ* [ppm] = 195.2, 160.3, 149.2, 145.4, 143.9, 136.7, 134.8, 126.6, 118.8, 109.0, 31.6, 22.9, 21.6, 7.3. HRMS (ESI) m/z: calculated for C_14_H_14_O_3_, [M+Na]^+^ 253.0835; found:253.0836.

**6,7-dimethyl-3-propionyl-1H-isochromen-1-one (3l)**

36.6 mg, 79% (isolated yield), yellow solid, mp 118.6-119.1 ^o^C, R_f_ = 0.31 (Petroleum ether/ Ether = 5/1). ^1^H NMR (600 MHz, CDCl_3_): *δ* [ppm] = 8.10 (s, 1H), 7.38 (s, 1H), 7.33 (s, 1H), 2.98 (q, *J* = 7.2 Hz, 2H), 2.41 (s, 3H), 1.19 (t, *J* = 7.2 Hz, 3H). ^13^C NMR (151 MHz, CDCl_3_): *δ* [ppm] = 195.1, 161.1, 149.1, 145.6, 140.7, 133.2, 130.3, 128.8, 120.7, 108.5, 31.7, 20.4, 20.1, 7.4. HRMS (ESI) m/z: calculated for C_14_H_14_O_3_, [M+Na]^+^ 253.0835;found:253.0834 .

**7-methoxy-8-methyl-3-propionyl-1H-isochromen-1-one (3m)**

23.6 mg, 48% (isolated yield), white solid, mp 147.1-148.0 ^o^C, R_f_ = 0.16 (Petroleum ether/ Ether = 5/1). ^1^H NMR (600 MHz, CDCl_3_): *δ* [ppm] = 7.45 (d, *J* = 8.5 Hz, 1H), 7.29 (d, *J* = 8.6 Hz, 1H), 7.28 (s, 1H), 3.94 (s, 3H), 2.95 (q, *J* = 7.2 Hz, 2H), 2.75 (s, 3H), 1.19 (t, *J* = 7.2 Hz, 3H). ^13^C NMR (151 MHz, CDCl_3_): *δ* [ppm] = 195.0, 160.5, 159.3, 147.5, 132.3, 129.0, 127.3, 122.3, 117.0, 109.4, 56.2, 31.5, 13.3, 7.4. HRMS (ESI) m/z: calculated for C_14_H_14_O_4_, [M+Na]^+^ 269.0784;found: 269.0778.

**7,8-dimethyl-3-propionyl-1H-isochromen-1-one (3n)**

25.3 mg, 55% (isolated yield), white solid, mp 141.7-142.3 ^o^C, R_f_ = 0.39 (Petroleum ether/ Ether = 5/1). ^1^H NMR (600 MHz, CDCl_3_): *δ* [ppm] = 7.55 (d, *J* = 7.9 Hz, 1H), 7.35 (d, *J* = 7.9 Hz, 1H), 7.27 (s, 1H), 2.97 (q, *J* = 7.2 Hz, 2H), 2.80 (s, 3H), 2.44 (s, 3H), 1.20 (t, *J* = 7.2 Hz, 3H).. ^13^C NMR (151 MHz, CDCl_3_): *δ* [ppm] = 195.1, 160.6, 148.5, 142.3, 140.7, 136.4, 134.7, 125.8, 121.2, 109.3, 31.6, 21.4, 17.6, 7.4. HRMS (ESI) m/z: calculated for C_14_H_14_O_3_, [M+Na]^+^

**5,6,7-trimethoxy-3-propionyl-1H-isochromen-1-one (3o)**

44.9 mg, 77% (isolated yield), yellow solid, mp 106.2-107.0 ^o^C, R_f_ = 0.19 (Petroleum ether/ Ether = 3/1). ^1^H NMR (400 MHz, CDCl_3_): *δ* [ppm] = 7.66 (s, 1H), 7.58 (s, 1H), 4.00 (s, 9H), 2.97 (q, *J* = 7.2 Hz, 2H), 1.21 (t, *J* = 7.2 Hz, 3H). ^13^C NMR (151 MHz, CDCl_3_): *δ* [ppm] = 194.7, 160.6, 155.9, 149.6, 148.0, 147.6, 124.4, 118.6, 106.6, 103.9, 61.9, 61.1, 56.4, 31.5, 7.4. HRMS (ESI) m/z: calculated for C_15_H_16_O_6_, [M+Na]^+^ 315.0839;found:315.0836.

253.0835;found: 253.0823.

**5-propionyl-7H-thieno[2,3-c]pyran-7-one (3p)**

24.9 mg, 54% (isolated yield), white solid, mp 103.7-104.1 ^o^C, R_f_ = 0.28 (Petroleum ether/ Ether = 3/1). ^1^H NMR (600 MHz, CDCl_3_): *δ* [ppm] = 7.92 (d, *J* = 5.1 Hz, 1H), 7.53 (s, 1H), 7.36 (d, *J* = 5.1 Hz, 1H), 3.01 (q, *J* = 7.2 Hz, 2H), 1.21 (t, *J* = 7.2 Hz, 3H). ^13^C NMR (151 MHz, CDCl_3_): *δ* [ppm] = 194.7, 156.7, 151.8, 145.2, 137.3, 127.8, 126.0, 104.8, 31.9, 7.3. HRMS (ESI) m/z: calculated for C_10_H_8_O_3_S, [M+Na]^+^ 231.0086;found:231.0087.

**3-acetyl-7-methyl-1H-isochromen-1-one (3q)**

30.8 mg, 76% (isolated yield), yellow solid, mp 123.6-124.1 ^o^C, R_f_ = 0.25 (Petroleum ether/ Ether = 5/1). ^1^H NMR (600 MHz, CDCl_3_): *δ* [ppm] = 8.13 (s, 1H), 7.59 (d, *J* = 7.9 Hz, 1H), 7.51 (d, *J* = 7.9 Hz, 1H), 7.34 (s, 1H), 2.54 (s, 3H), 2.49 (s, 3H). ^13^C NMR (151 MHz, CDCl_3_): *δ* [ppm] = 192.0, 161.0, 148.8, 141.7, 136.4, 132.4, 129.8, 128.2, 122.7, 109.0, 26.1, 21.6. HRMS (ESI) m/z: calculated for C_12_H_10_O_3_, [M+Na]^+^ 225.0522;found:225.0522.

**3-hexanoyl-7-methyl-1H-isochromen-1-one (3r)**

26.3 mg, 51% (isolated yield), white solid, mp 115.5-116.3 ^o^C, R_f_ = 0.44 (Petroleum ether/ Ether = 5/1). ^1^H NMR (400 MHz, CDCl_3_): *δ* [ppm] = 8.16 (s, 1H), 7.60 (d, *J* = 7.9 Hz, 1H), 7.53 (d, *J* = 7.9 Hz, 1H), 7.36 (s, 1H), 2.94 (t, *J* = 7.3 Hz, 2H), 2.51 (s, 3H), 1.78 – 1.66 (m, 2H), 1.36 (d, *J* = 3.2 Hz, 4H), 0.91 (t, *J* = 6.2 Hz, 3H). ^13^C NMR (151 MHz, CDCl_3_): *δ* [ppm] = 194.6, 161.1, 149.0, 141.5, 136.4, 132.7, 129.9, 128.2, 122.8, 108.6, 38.2, 31.3, 23.1, 22.4, 21.6, 13.9. HRMS (ESI) m/z: calculated for C_16_H_18_O_3_, [M+Na]^+^ 281.1148;found:281.1146.

1. The conversions of acids

**Table S1** The conversions of starting materials

| **Entry** | **Reactant** | **Compound** | **Conversion** |
| --- | --- | --- | --- |
| 1 |  | **1a** | 85% |
| 2 |  | **1b** | **79%** |
| 3 |  | **1c** | 81% |
| 4 |  | **1d** | 91% |
| 5 |  | **1e** | --- |
| 6 |  | **1f** | 83% |
| 7 |  | **1g** | 100% |
| 8 |  | **1h** | 93% |
| 9 |  | **1i** | 100% |
| 10 |  | **1j** | 97% |
| 11 |  | **1k** | 94% |
| 12 |  | **1l** | 89% |
| 13 |  | **1m** | 87% |
| 14 |  | **1n** | --- |
| 15 |  | **1o** | 92% |
| 16 |  | **1p** | 99% |
| 17 |  | **1q** | 99% |
| 18 |  | **1r** | 100% |
| 19 |  | **1s** | 100% |
| 20 |  | **1t** | 99% |
| 21 |  | **1u** | 87% |
| 22 |  | **1v** | 97% |
| 23 |  | **1w** | 94% |
| 24 |  | **1x** | 88% |
| 25 |  | **1y** | --- |
| 26 |  | **1z** | 74% |
| 27 |  | **1aa** | --- |

1. The conversions of acids

**Table S2** The conversions of starting materials

| **Entry** | **Reactant** | **Compound** | **Conversion** |
| --- | --- | --- | --- |
| 1 |  | **1a** | 100% |
| 2 |  | **1b** | 99% |
| 3 |  | **1c** | 100% |
| 4 | **** | **1d** | 92% |
| 5 |  | **1e** | 100% |
| 6 |  | **1f** | 95% |
| 7 |  | **1g** | 100% |
| 8 |  | **1h** | 90% |
| 9 |  | **1i** | 97% |
| 10 |  | **1j** | 100% |
| 11 |  | **1k** | 94% |
| 12 |  | **1l** | 97% |
| 13 |  | **1m** | 93% |
| 14 |  | **1n** | 100% |
| 15 |  | **1o** | 90% |
| 16 |  | **1p** | 99% |
| 17 |  | **1q** | 96% |
| 18 |  | **1r** | 97% |

1. Copies of NMR spectra

**^
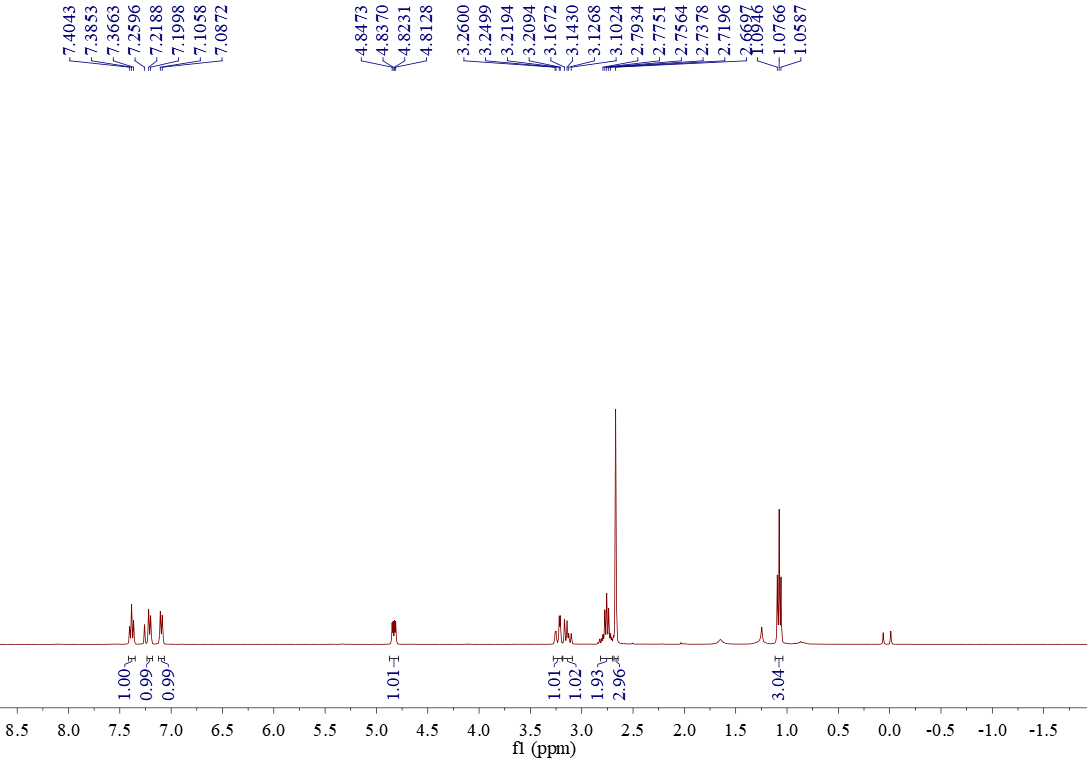
^**

**Attached Fig.1** ^1^H NMR spectra of compound **2a**


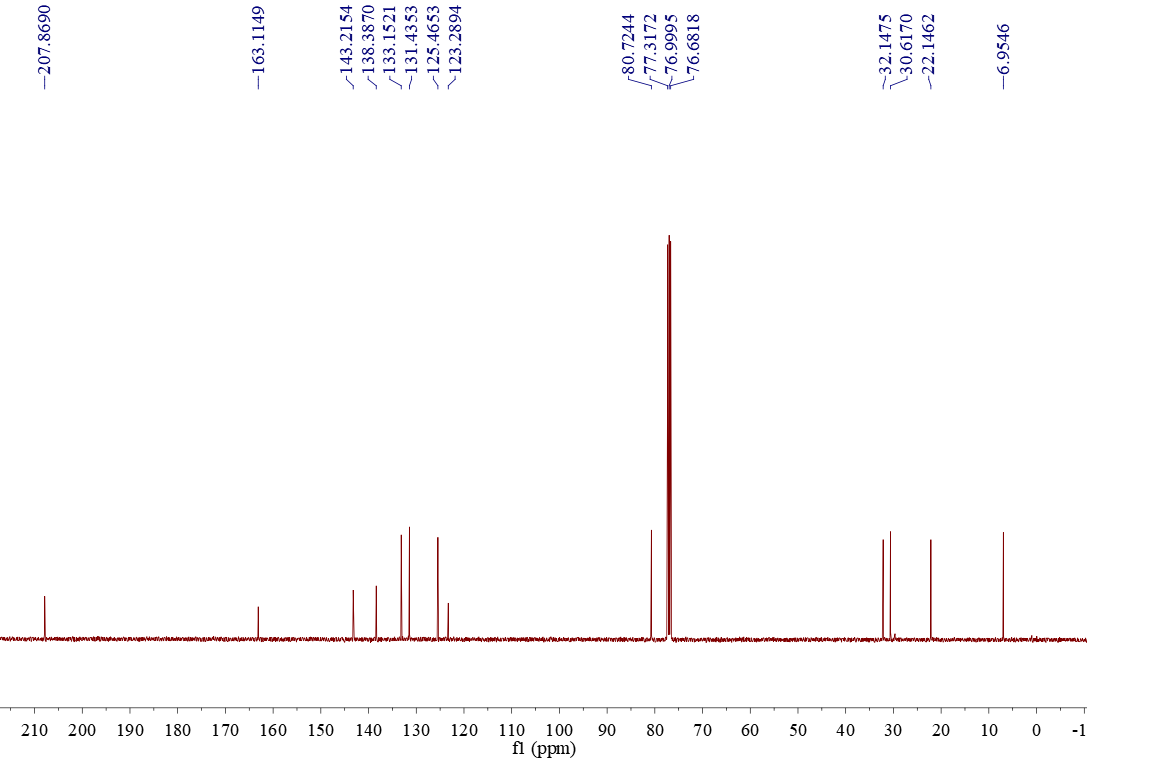


**Attached Fig.2 ^13^C NMR** spectra of compound **2a**

**^
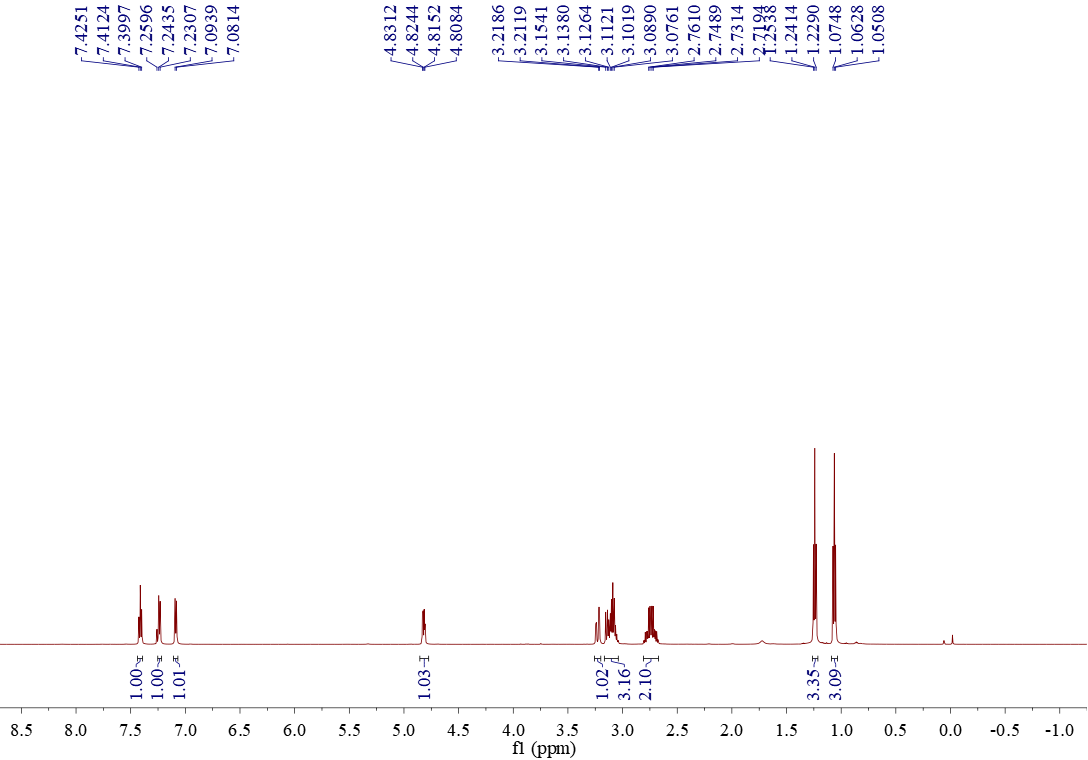
^**

**Attached Fig.3** ^1^H NMR spectra of compound **2b**

**
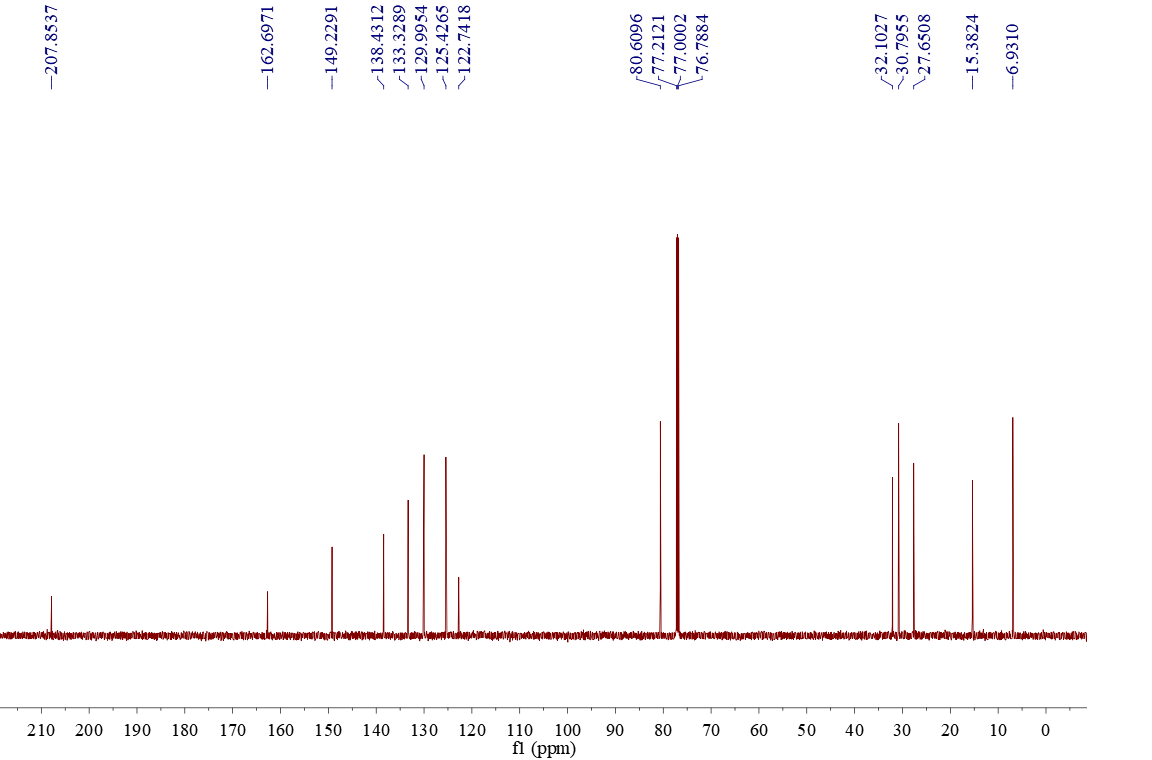
**

**Attached Fig.4** ^13^C NMR spectra of compound **2b**

**^
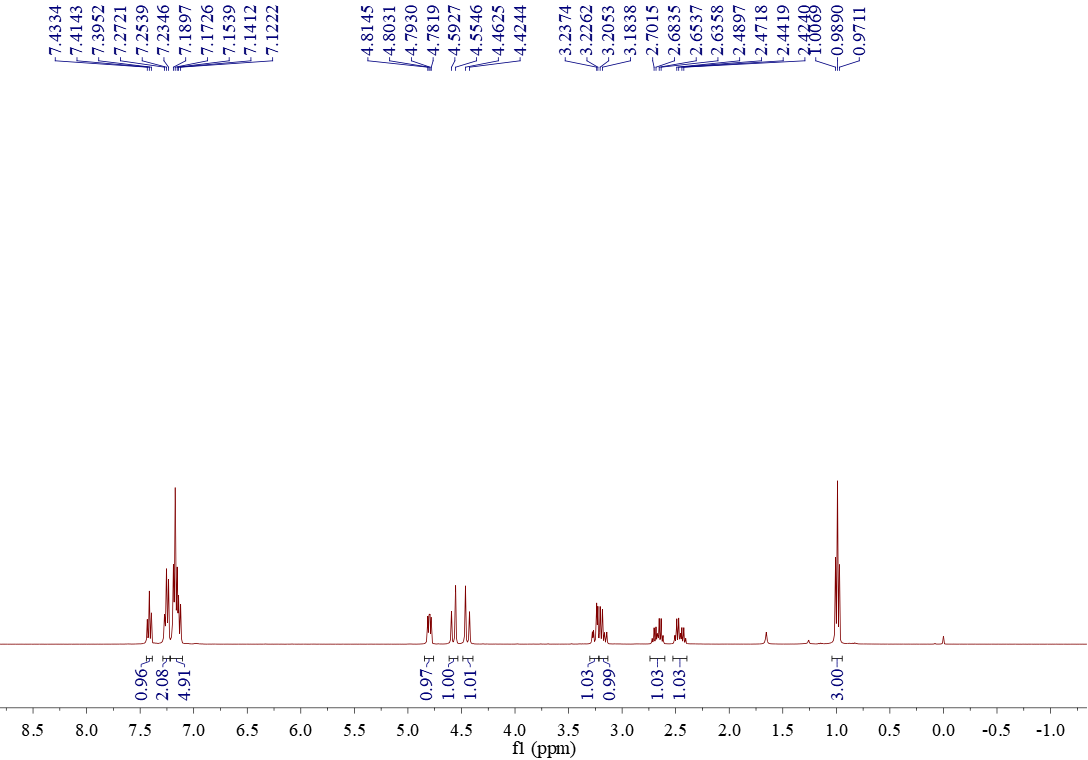
^**

**Attached Fig.5** ^1^H NMR spectra of compound **2c**

**^^
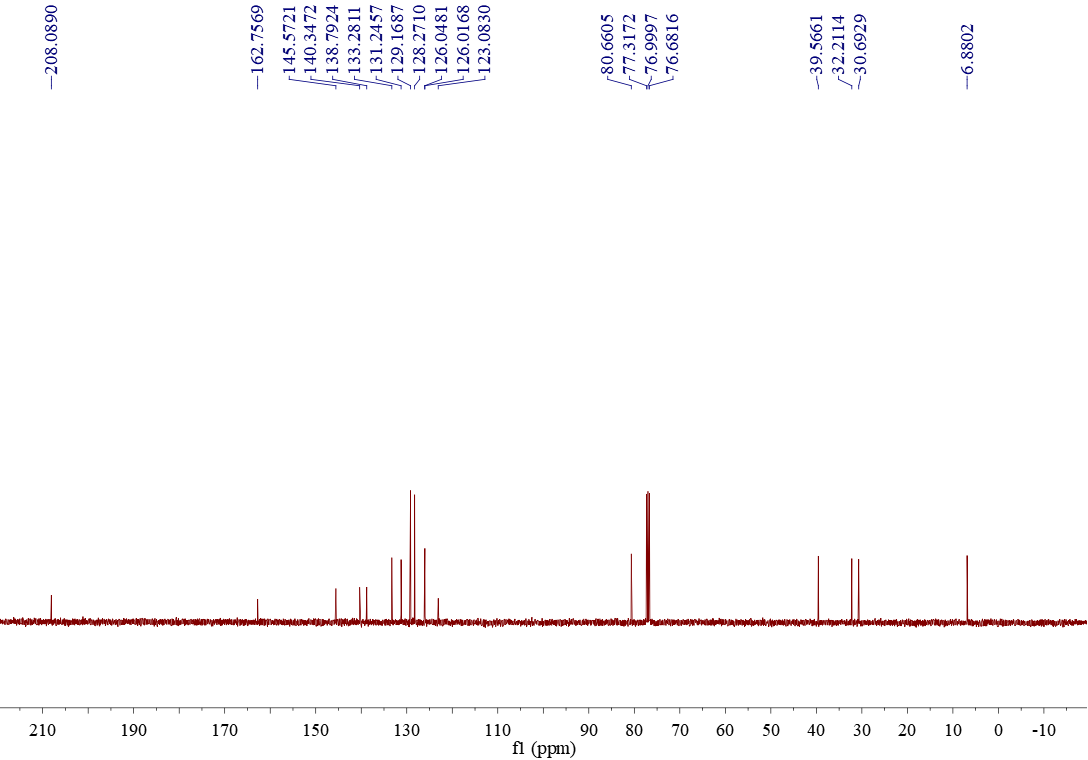
**

**Attached Fig.6** ^13^C NMR spectra of compound **2c**

**^
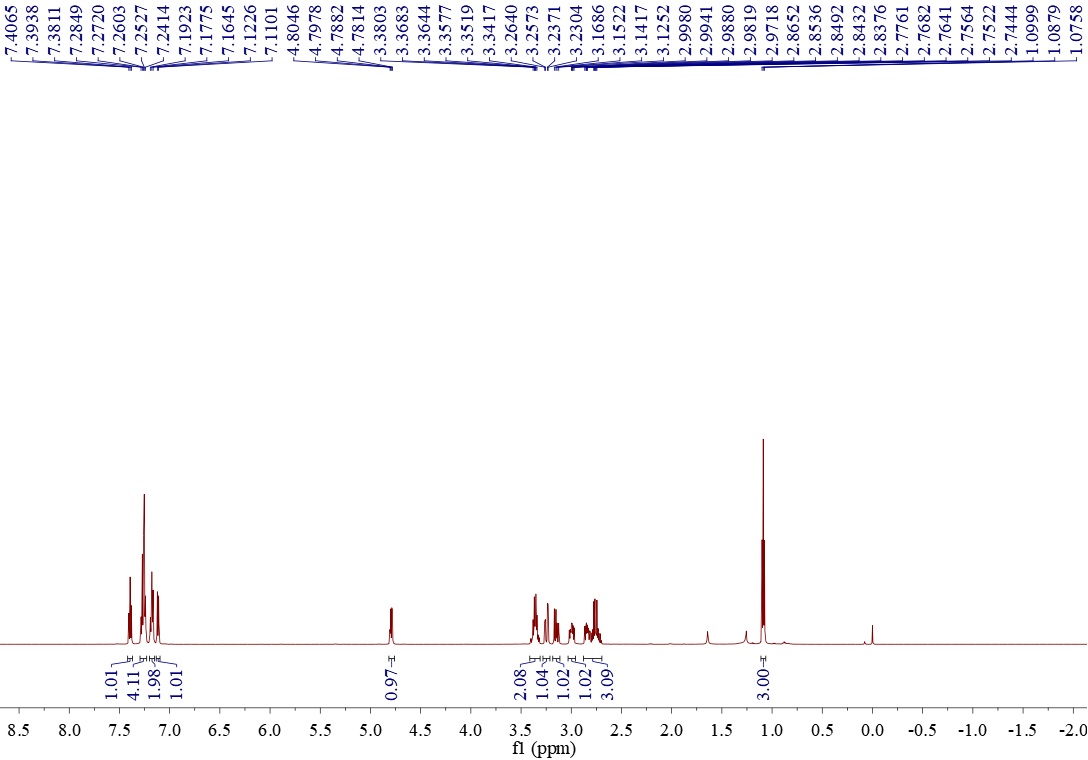
^**

**Attached Fig.7** ^1^H NMR spectra of compound **2d**


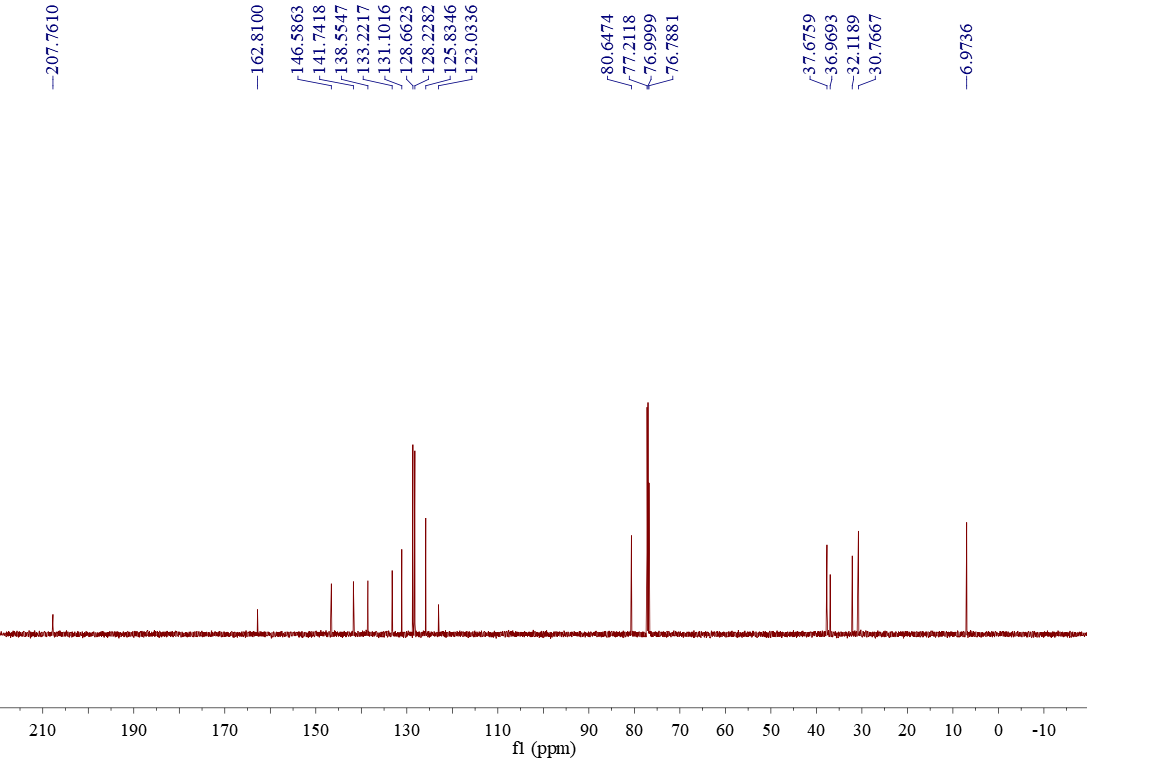


**Attached Fig.8** ^13^C NMR spectra of compound **2d**

**^
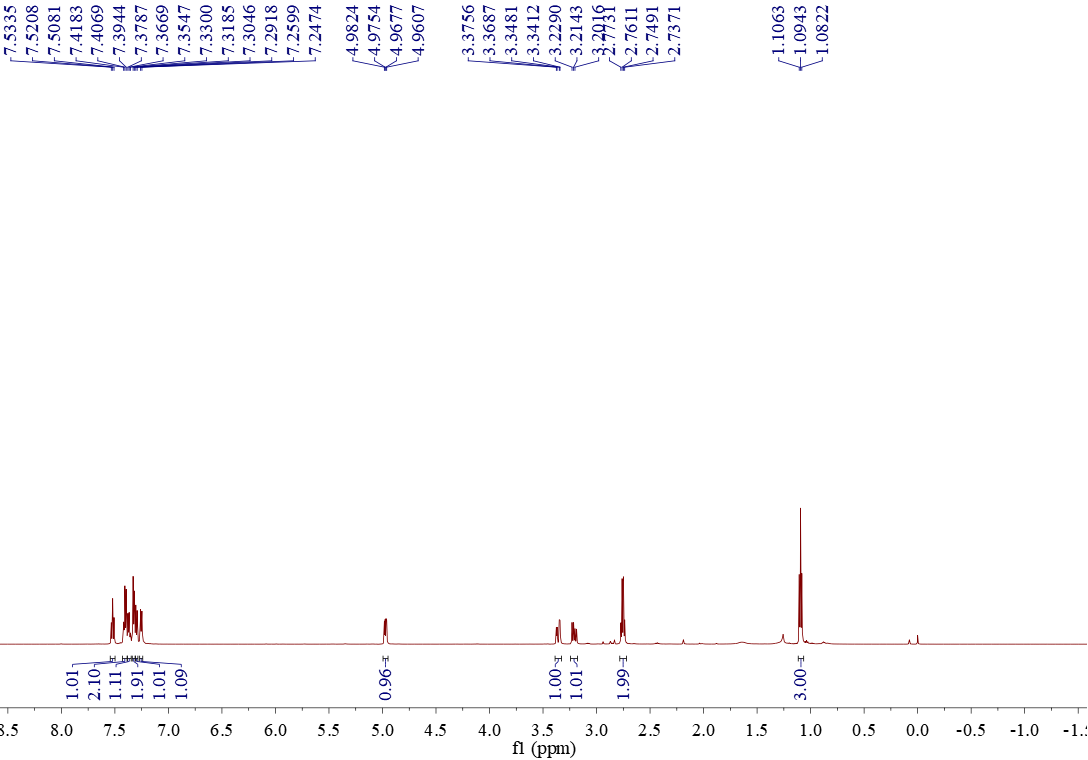
^**

**Attached Fig.9** ^1^H NMR spectra of compound **2e**

**
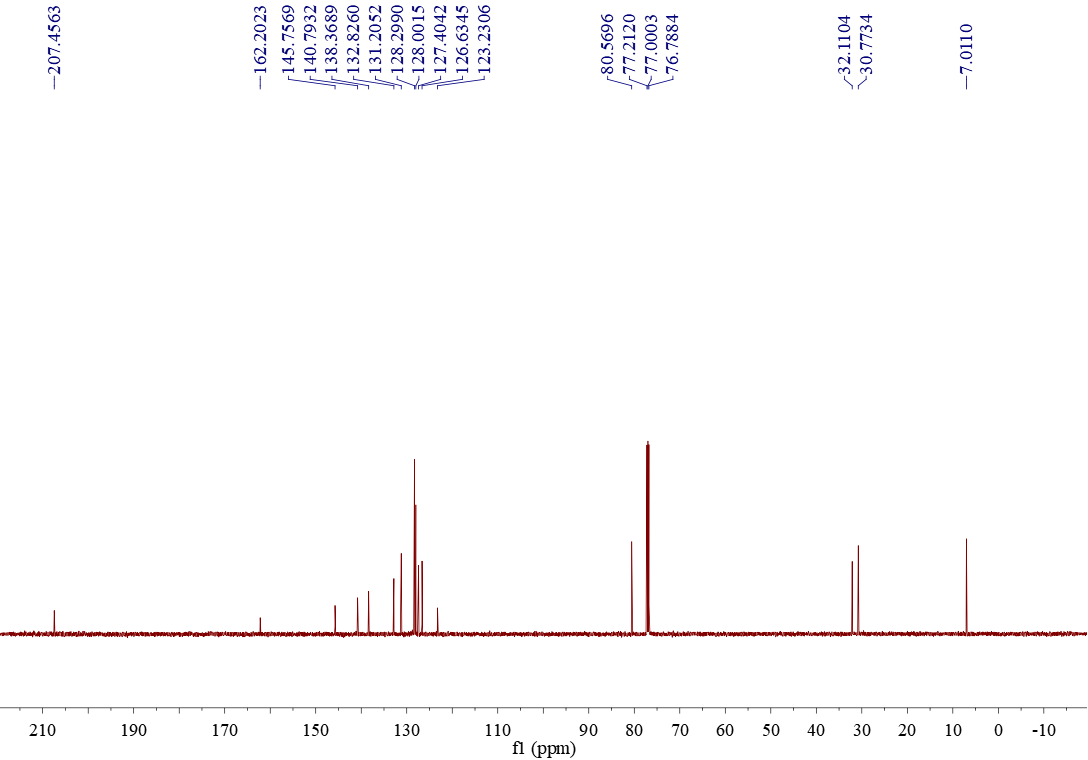
**

**Attached Fig.10** ^13^C NMR spectra of compound **2e**

**^
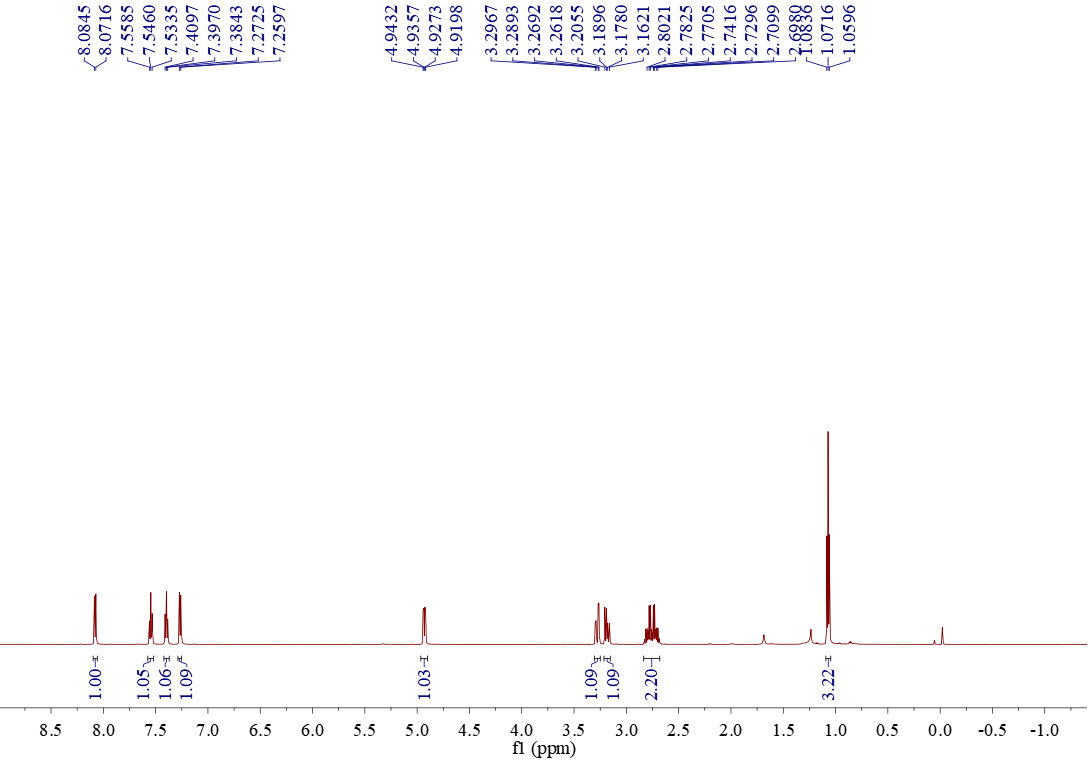
^**

**Attached Fig.11** ^1^H NMR spectra of compound **2f**

**^13^C NMR spectra of compound 3f**

**
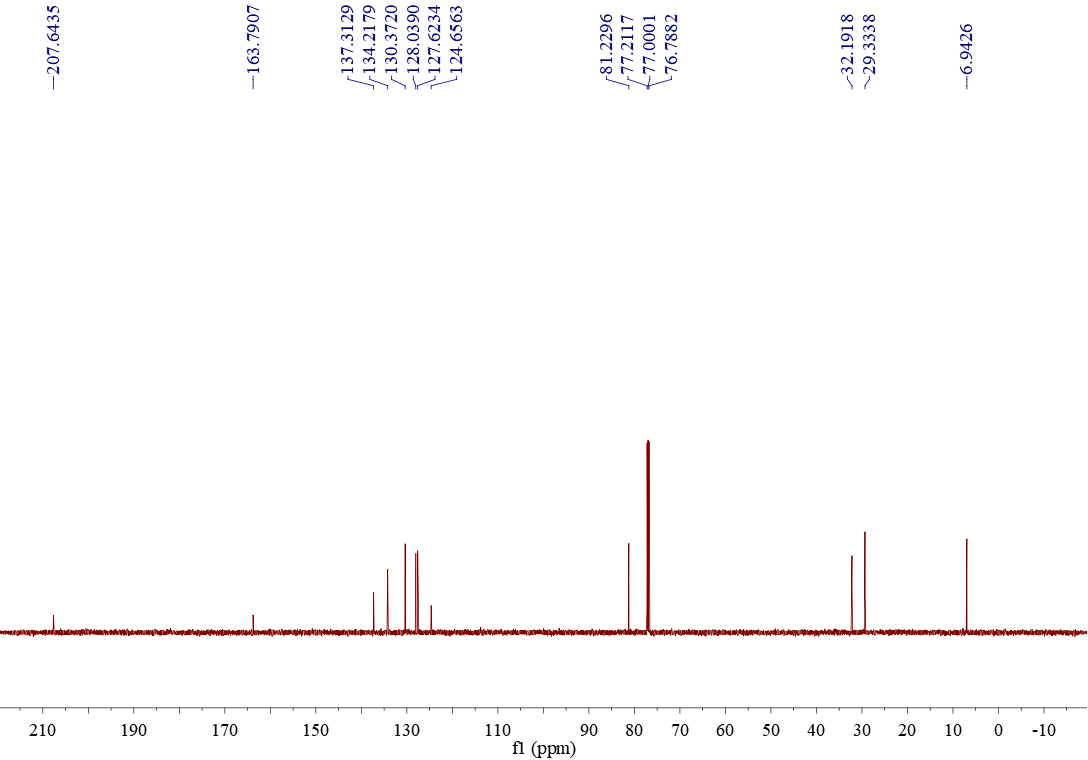
**

**Attached Fig.12** ^13^C NMR spectra of compound **2f**

**^
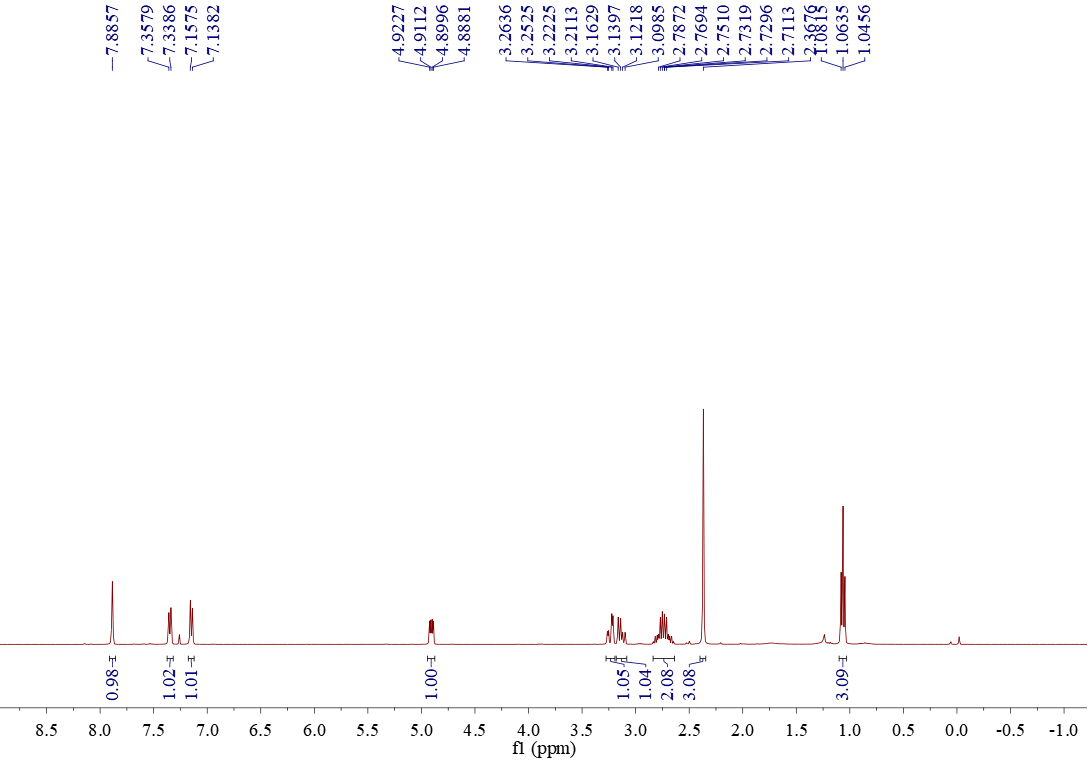
^**

**Attached Fig.13** ^1^H NMR spectra of compound **2g**

**^13^C NMR spectra of compound 3g**

**
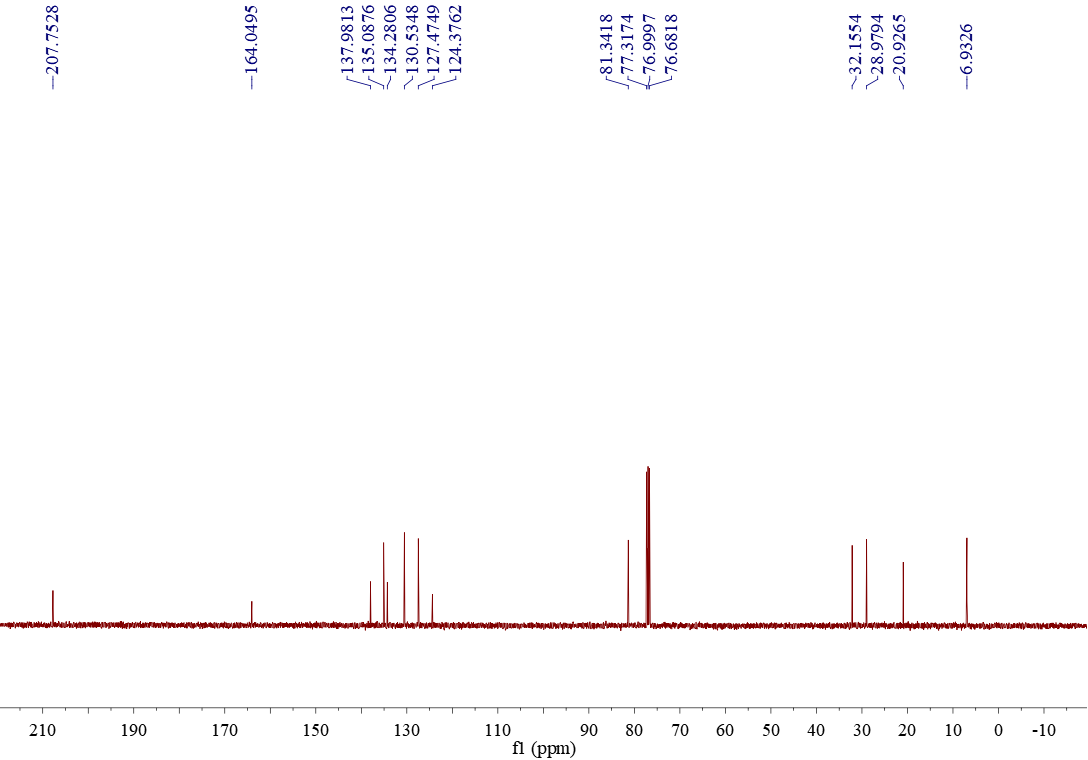
**

**Attached Fig.14** ^13^C NMR spectra of compound **2g**

**^
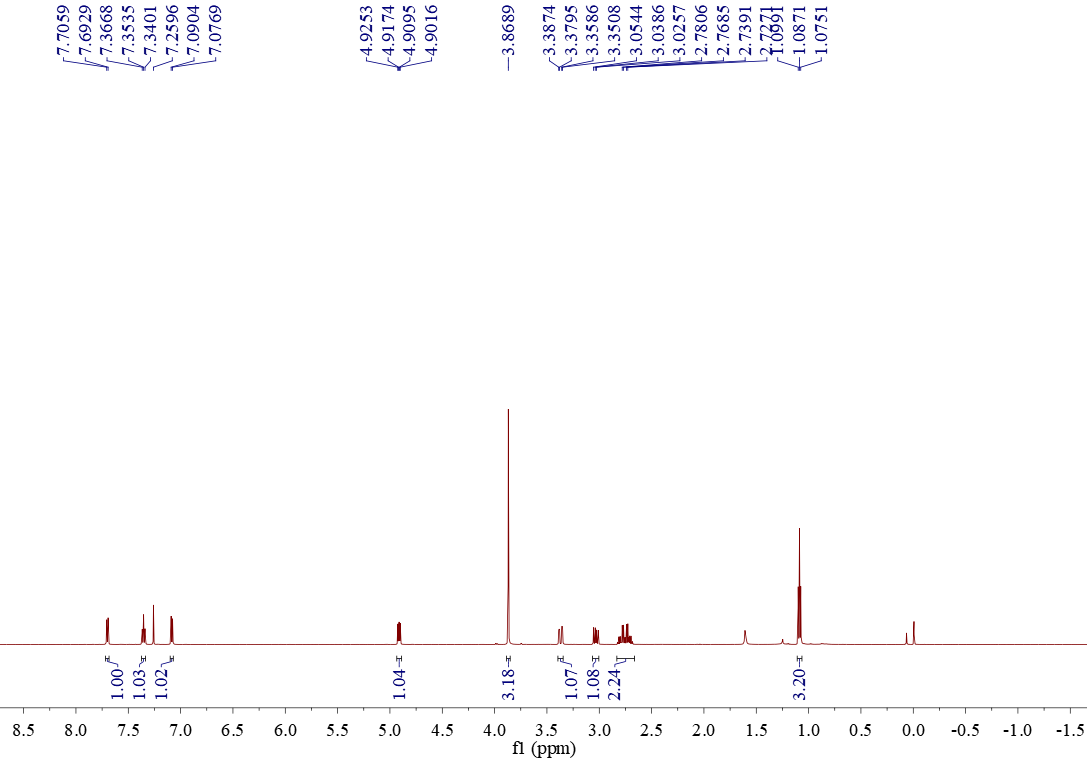
^**

**Attached Fig.15** ^1^H NMR spectra of compound **2h**


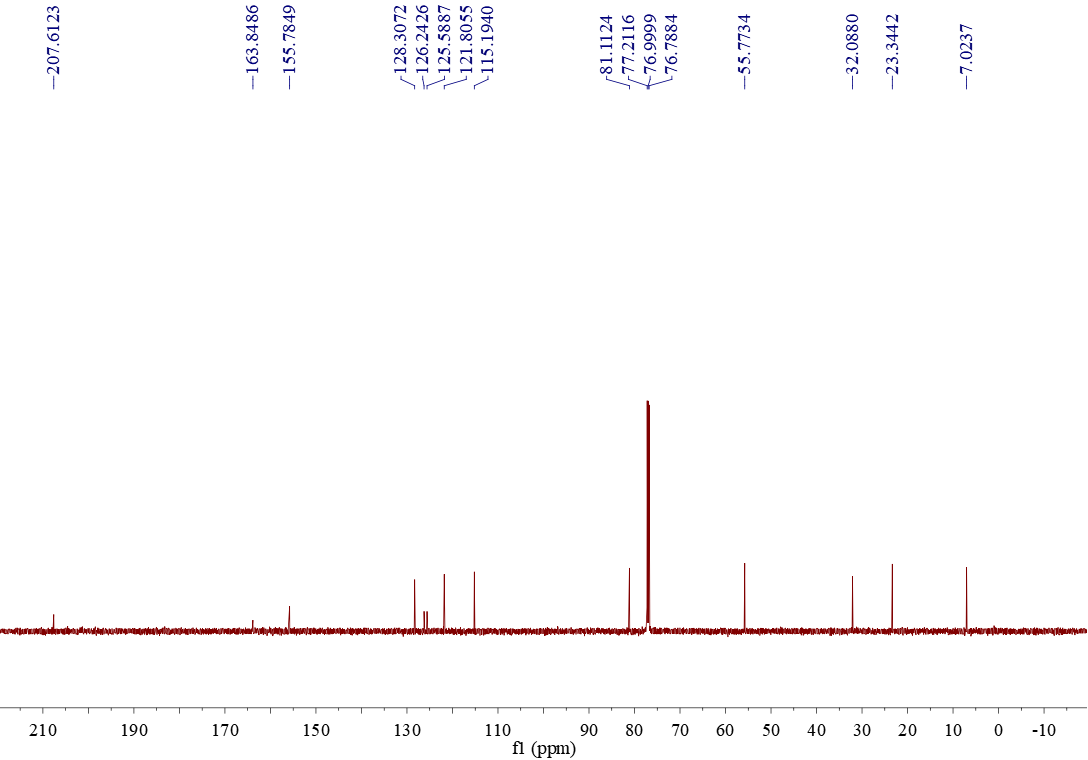


**Attached Fig.16** ^13^C NMR spectra of compound **2h**

**^
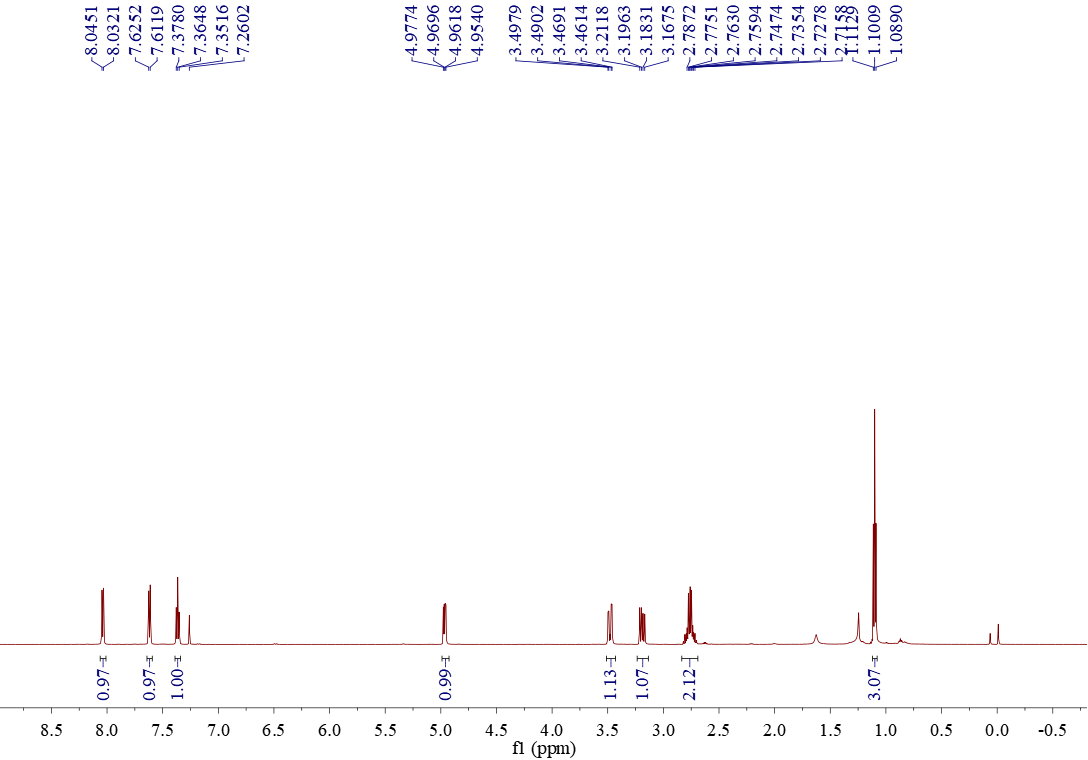
^**

**Attached Fig.17** ^1^H NMR spectra of compound **2i**

**^13^C NMR spectra of compound 3i**


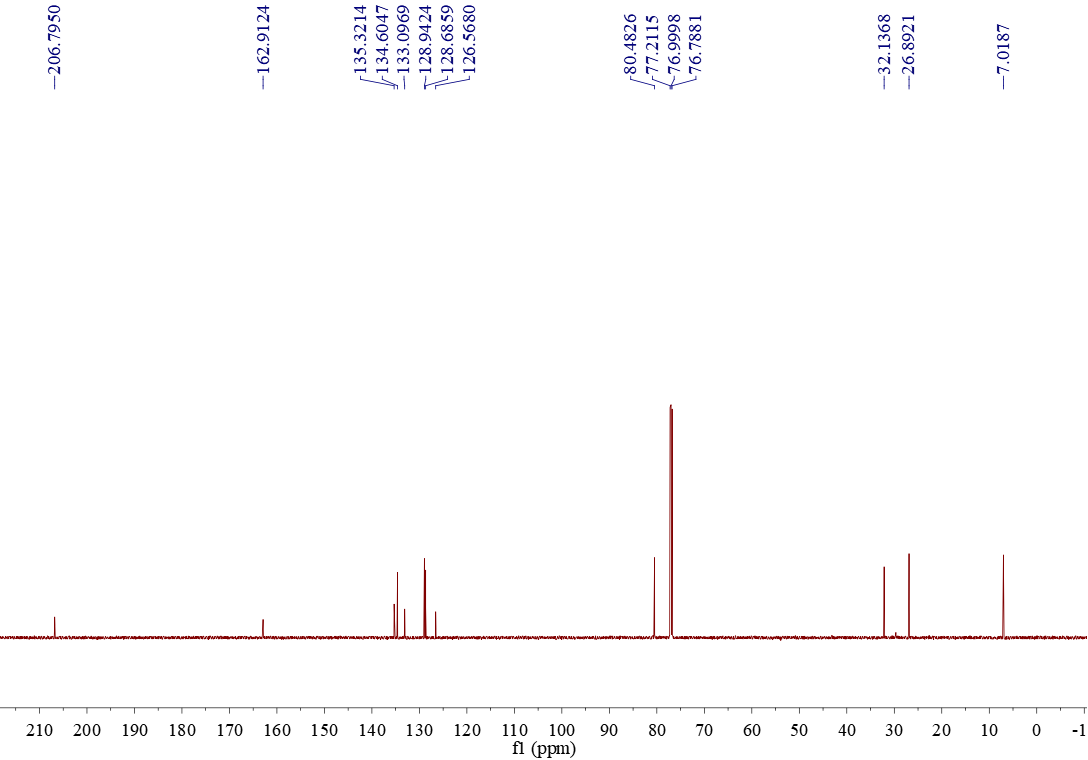


**Attached Fig.18** ^13^C NMR spectra of compound **2i**

**^
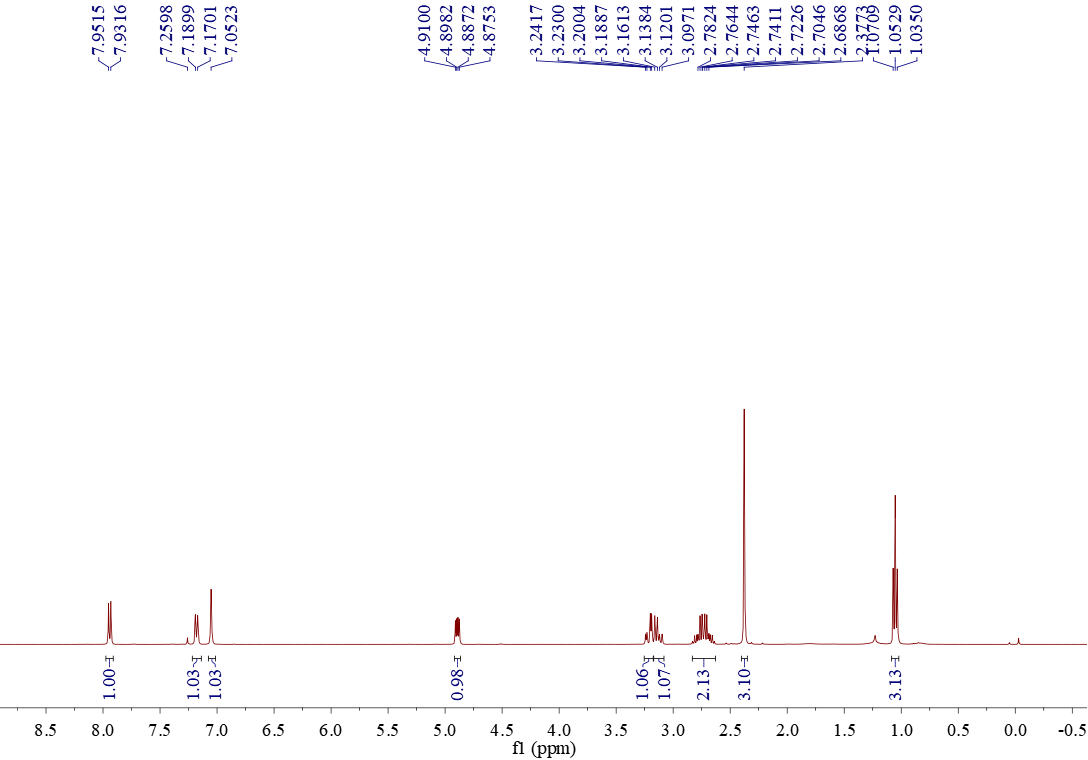
^**

**Attached Fig.19** ^1^H NMR spectra of compound **2j**


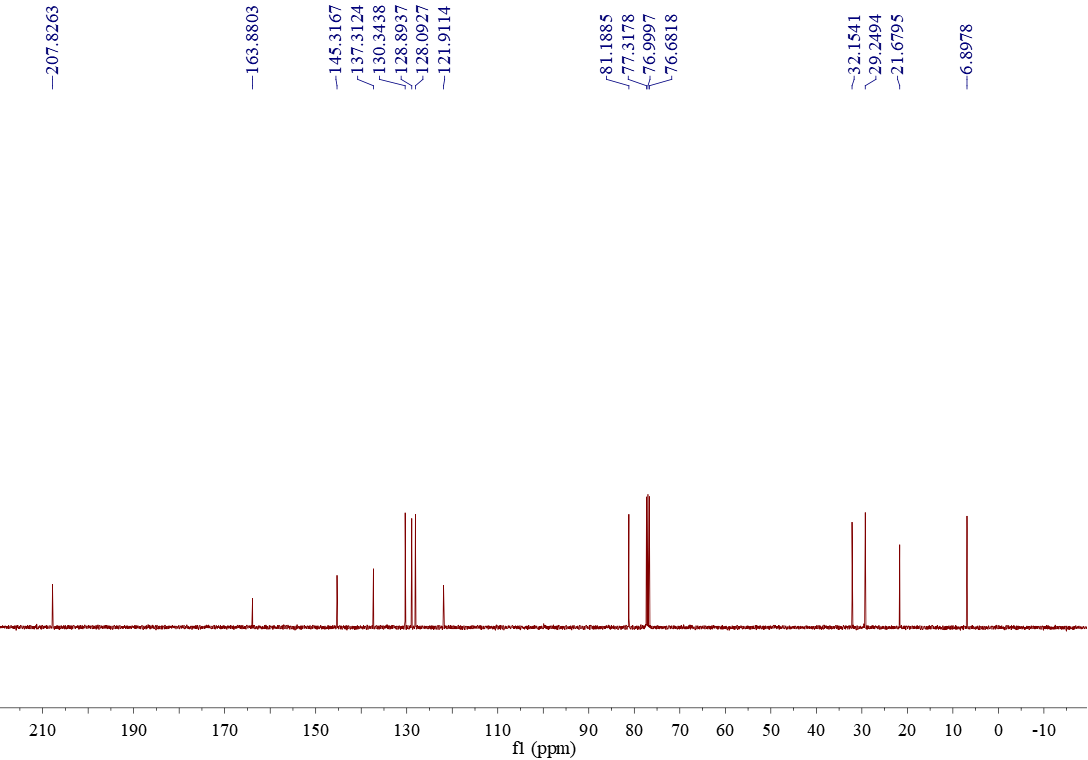


**Attached Fig.20** ^13^C NMR spectra of compound **2j**

**^1^H NMR spectra of compound 3k**

**
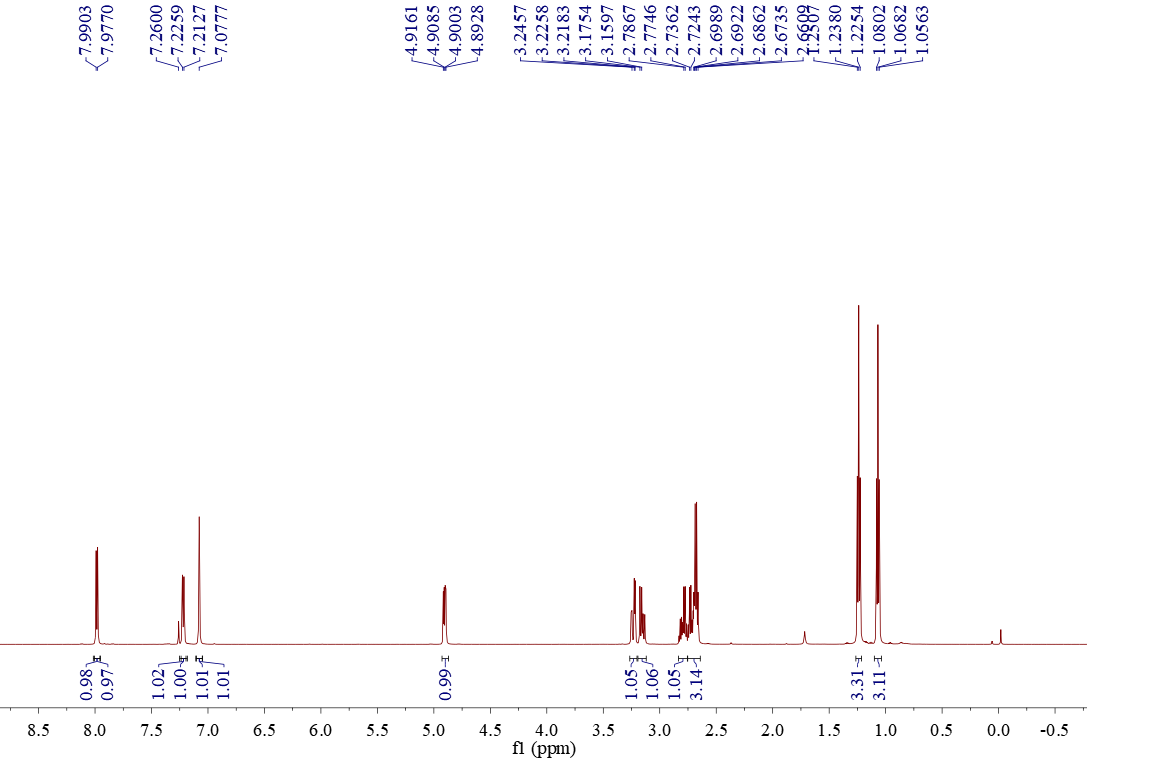
**

**Attached Fig.21** ^1^H NMR spectra of compound **2k**

**
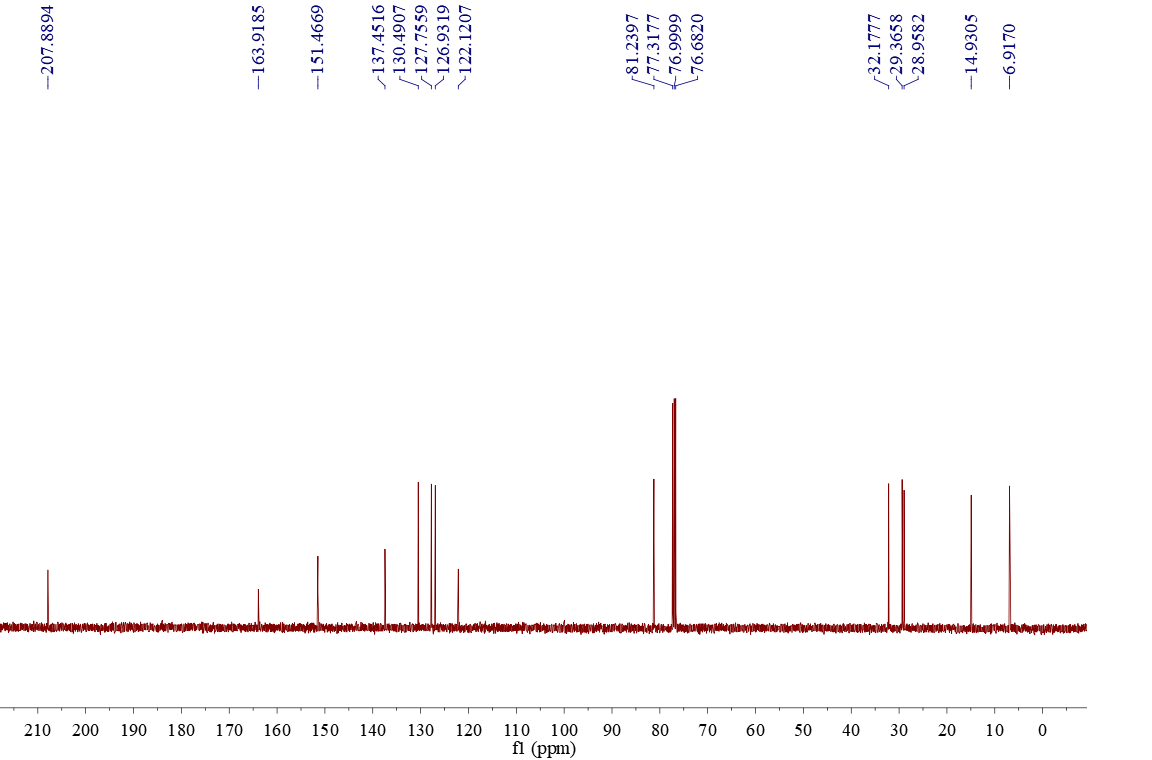
**

**Attached Fig.22** ^13^C NMR spectra of compound **2k**

**
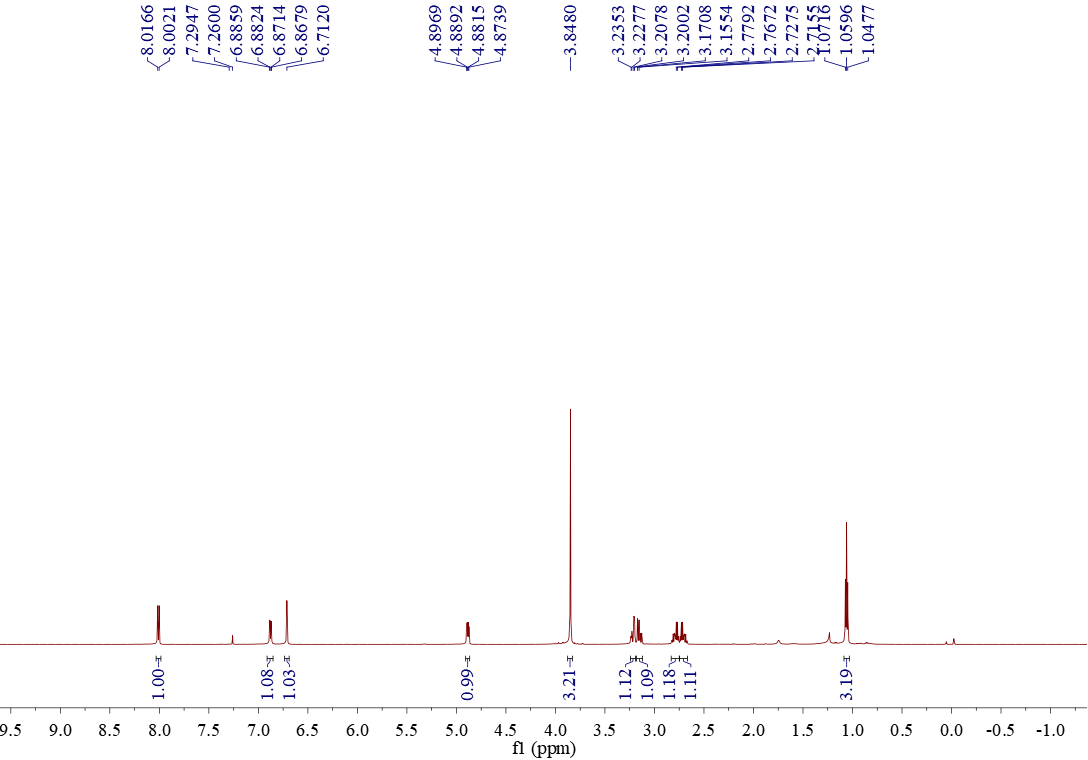
**

**Attached Fig.23** ^1^H NMR spectra of compound **2l**

**
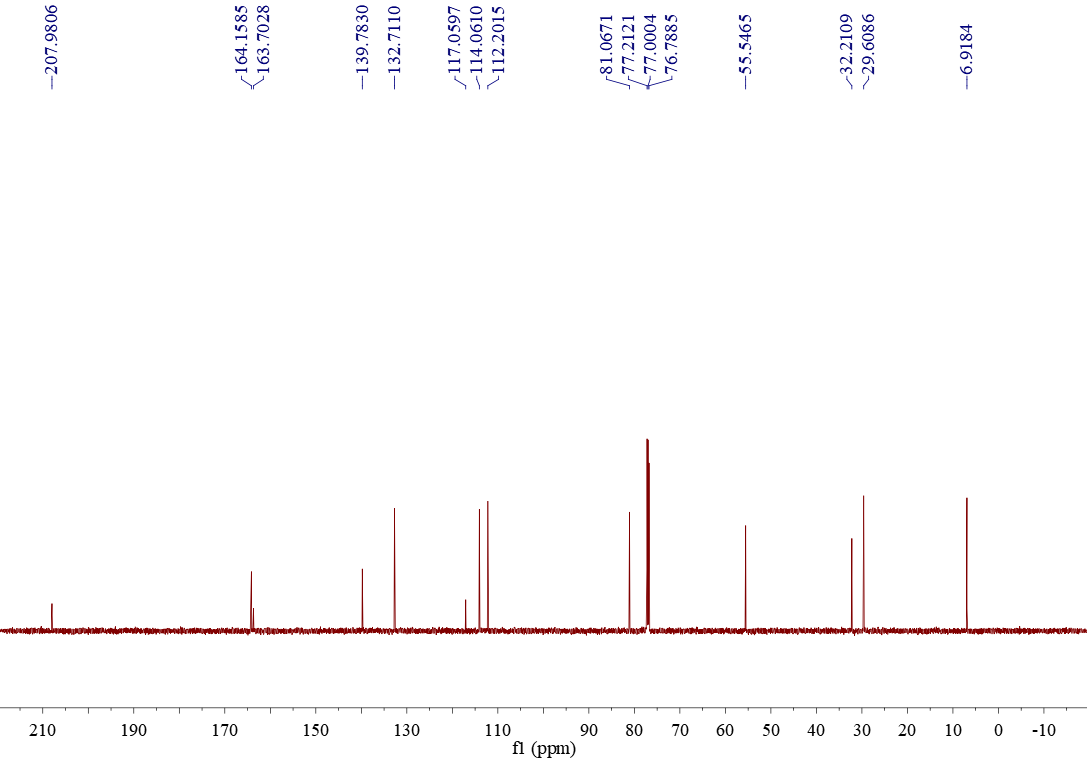
**

**Attached Fig.24** ^13^C NMR spectra of compound **2l**

**
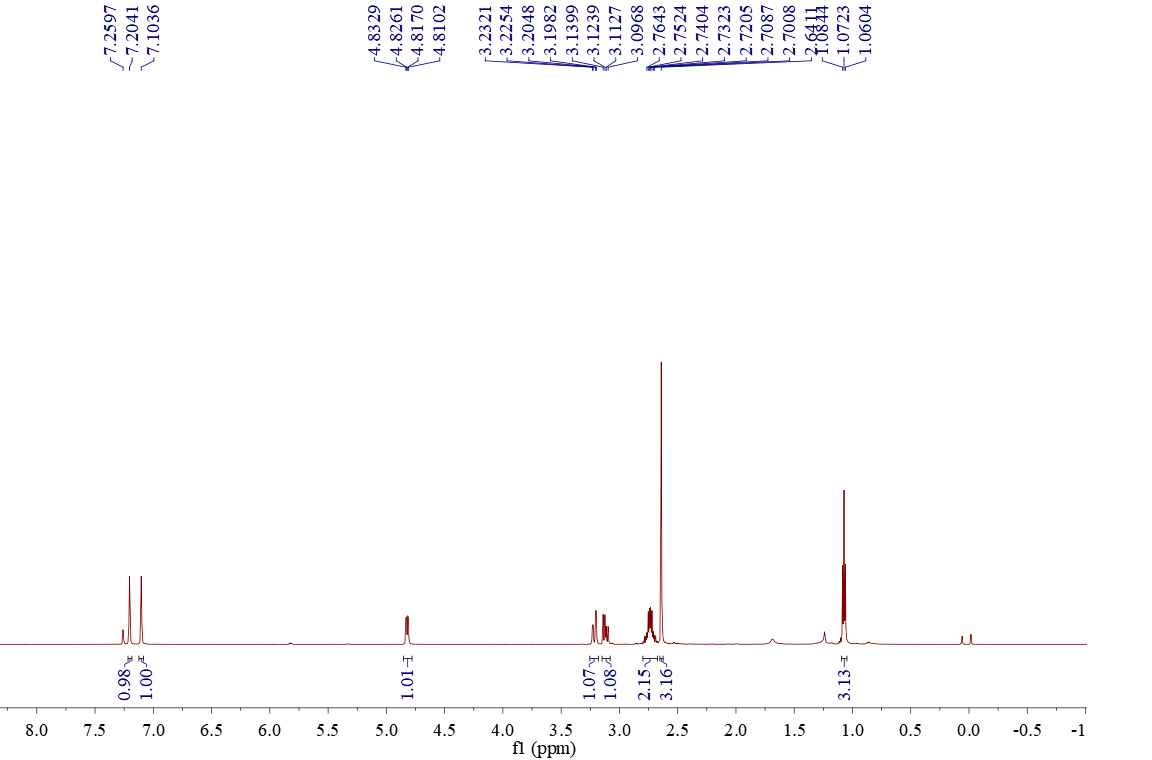
**

**Attached Fig.25** ^1^H NMR spectra of compound **2m**

**
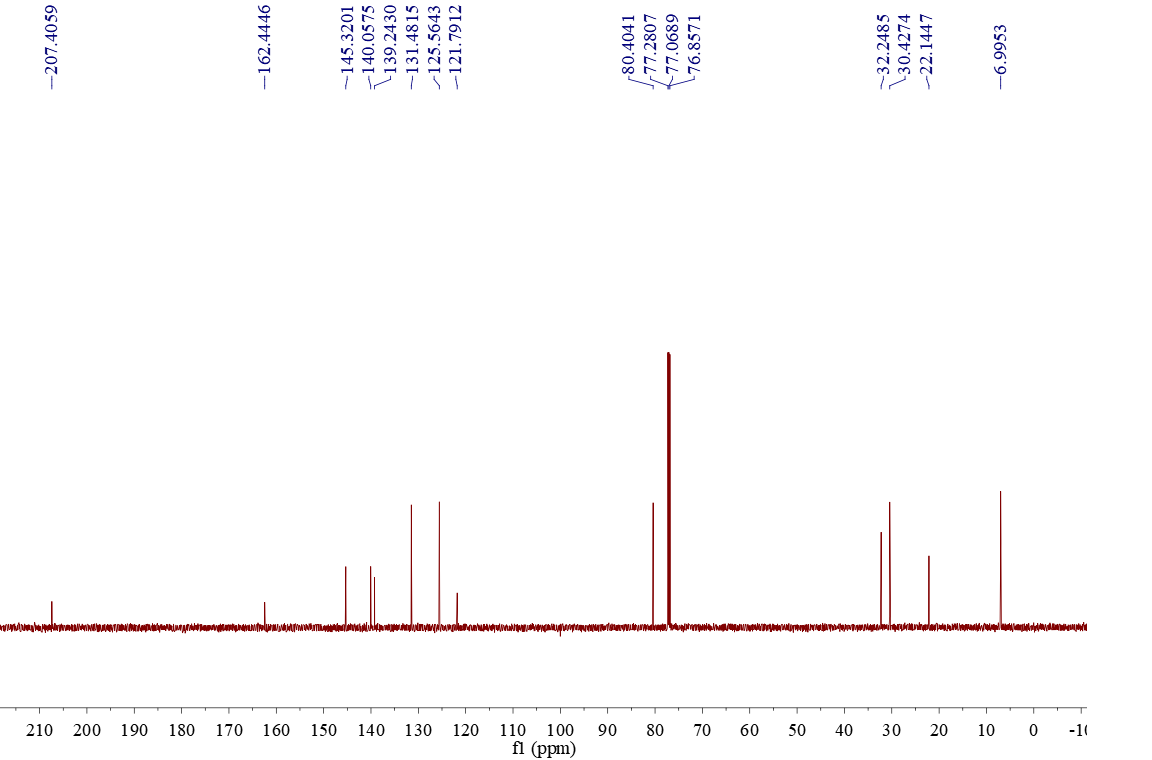
**

**Attached Fig.26** ^13^C NMR spectra of compound **2m**

**
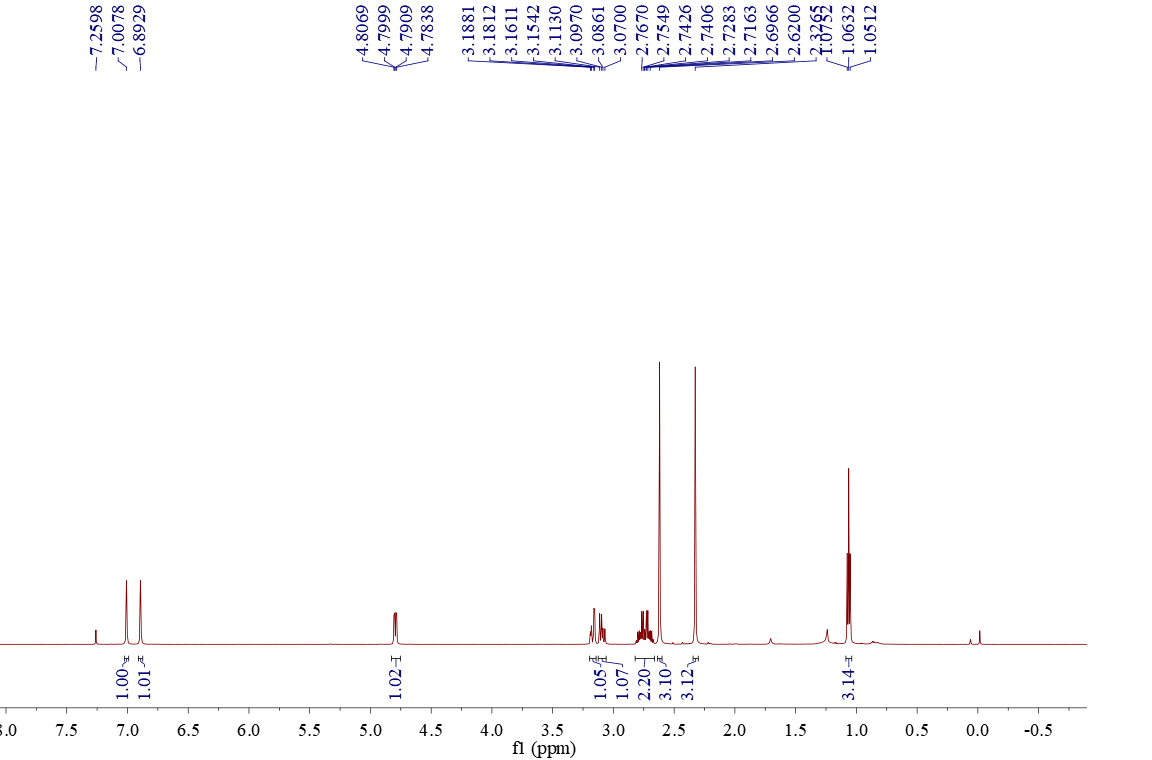
**

**Attached Fig.27** ^1^H NMR spectra of compound **2n**

**
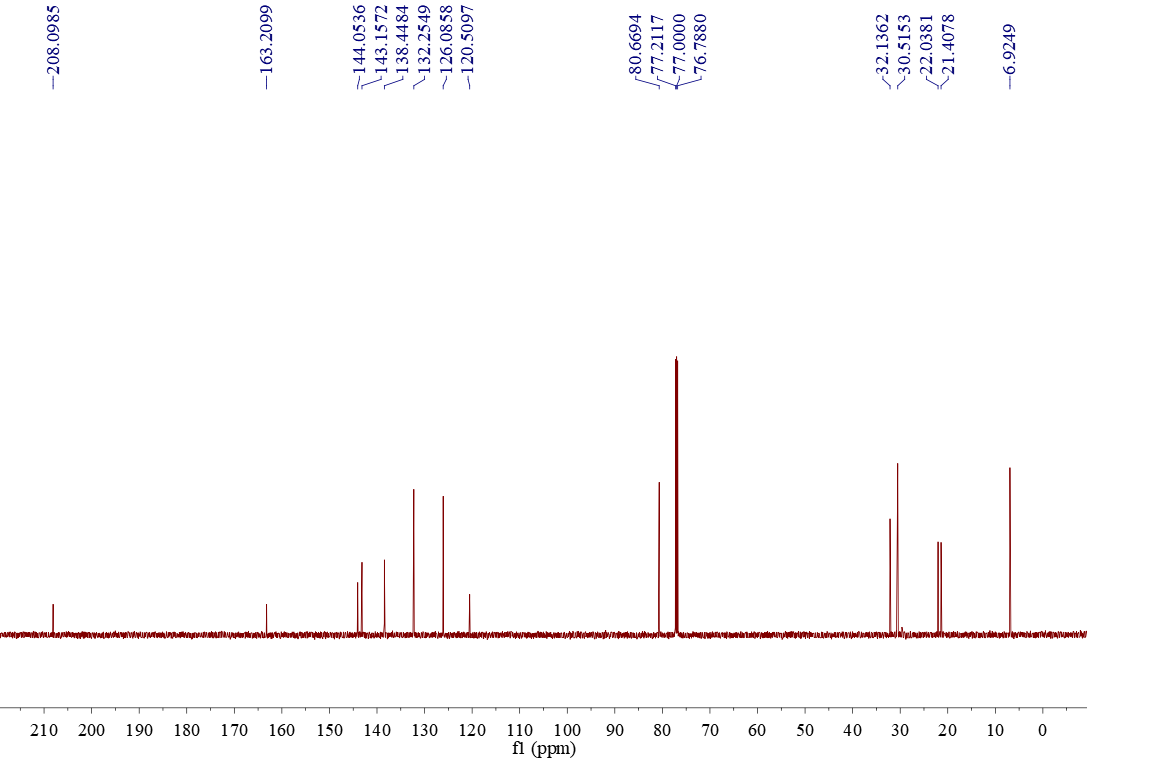
**

**Attached Fig.28** ^13^C NMR spectra of compound **2n**

**^
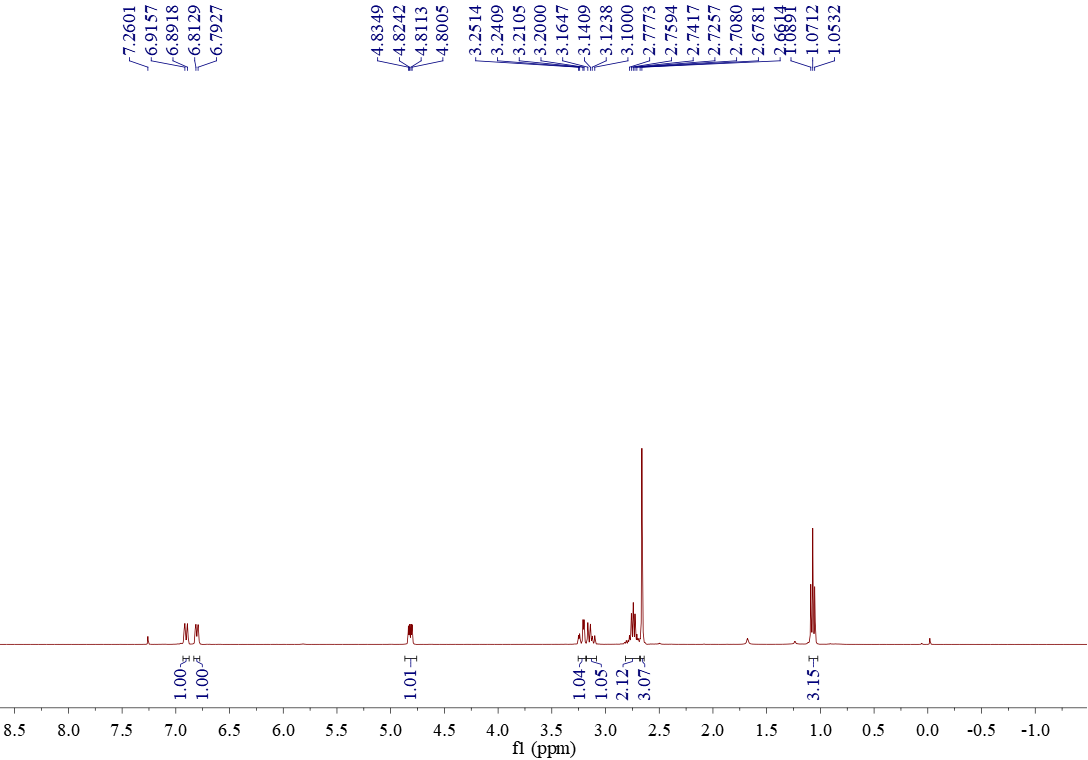
^**

**Attached Fig.29** ^1^H NMR spectra of compound **2o**

**
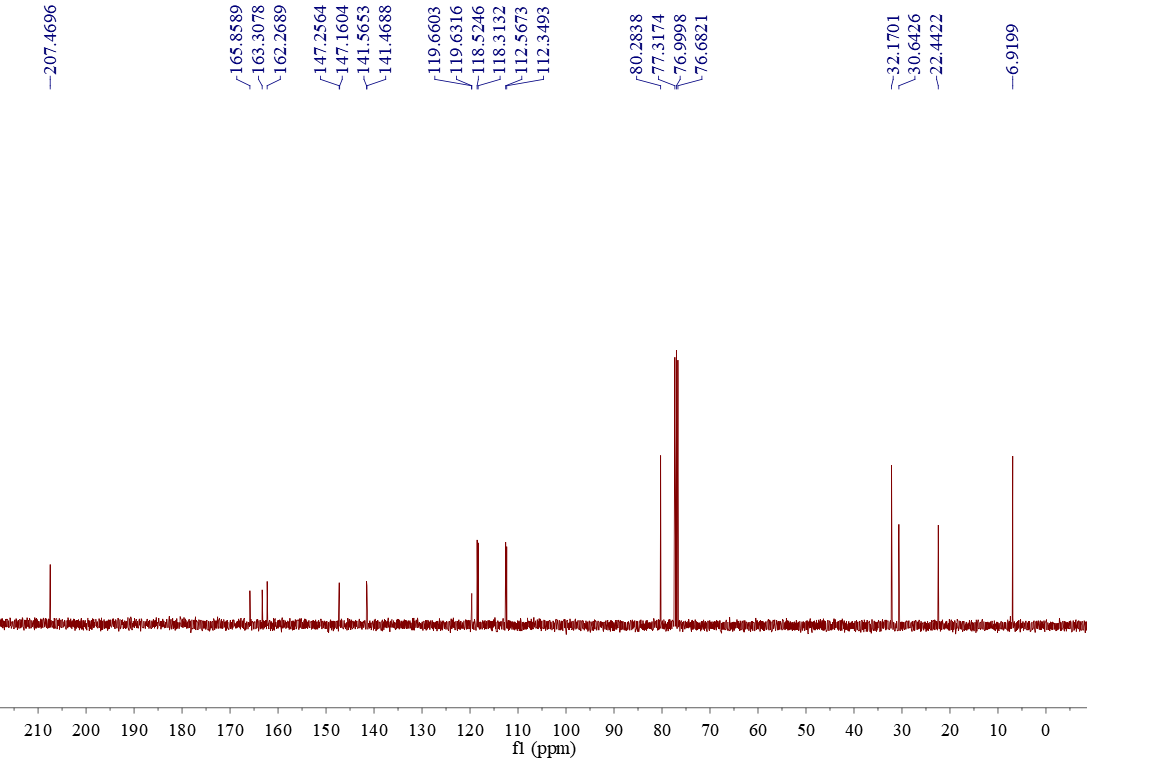
**

**Attached Fig.30** ^13^C NMR spectra of compound **2o**

**
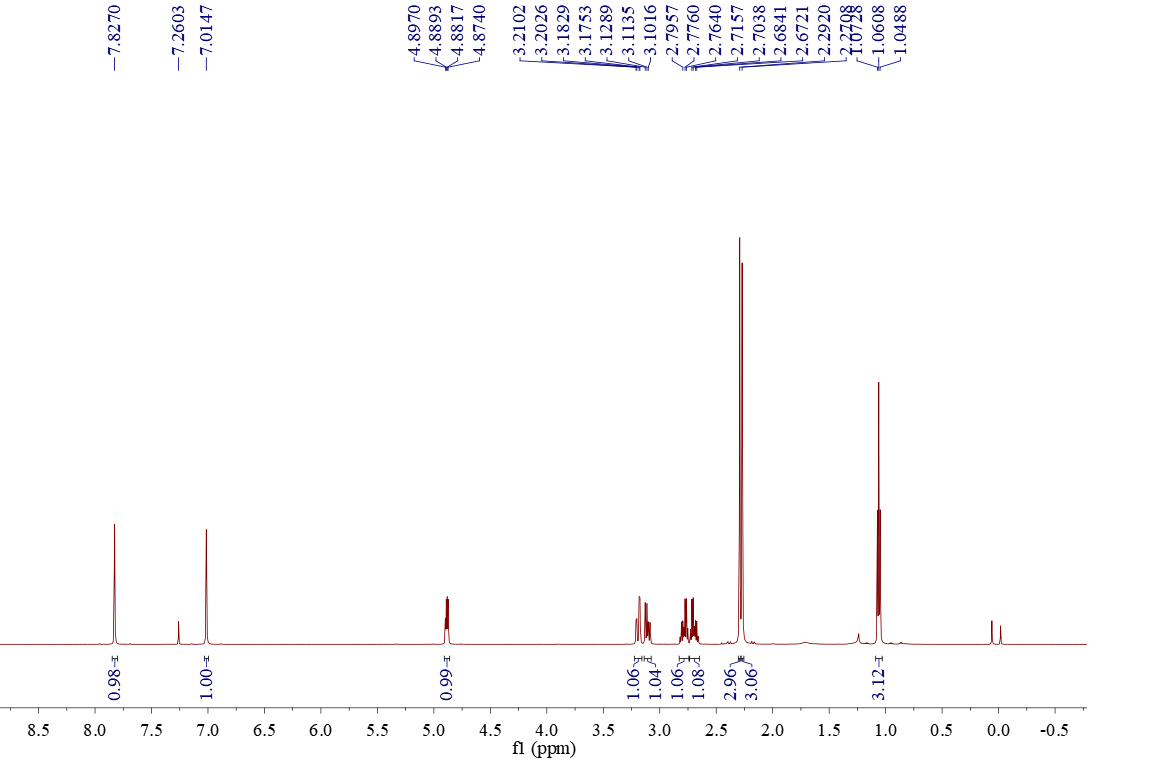
**

**Attached Fig.31** ^1^H NMR spectra of compound **2p**

**
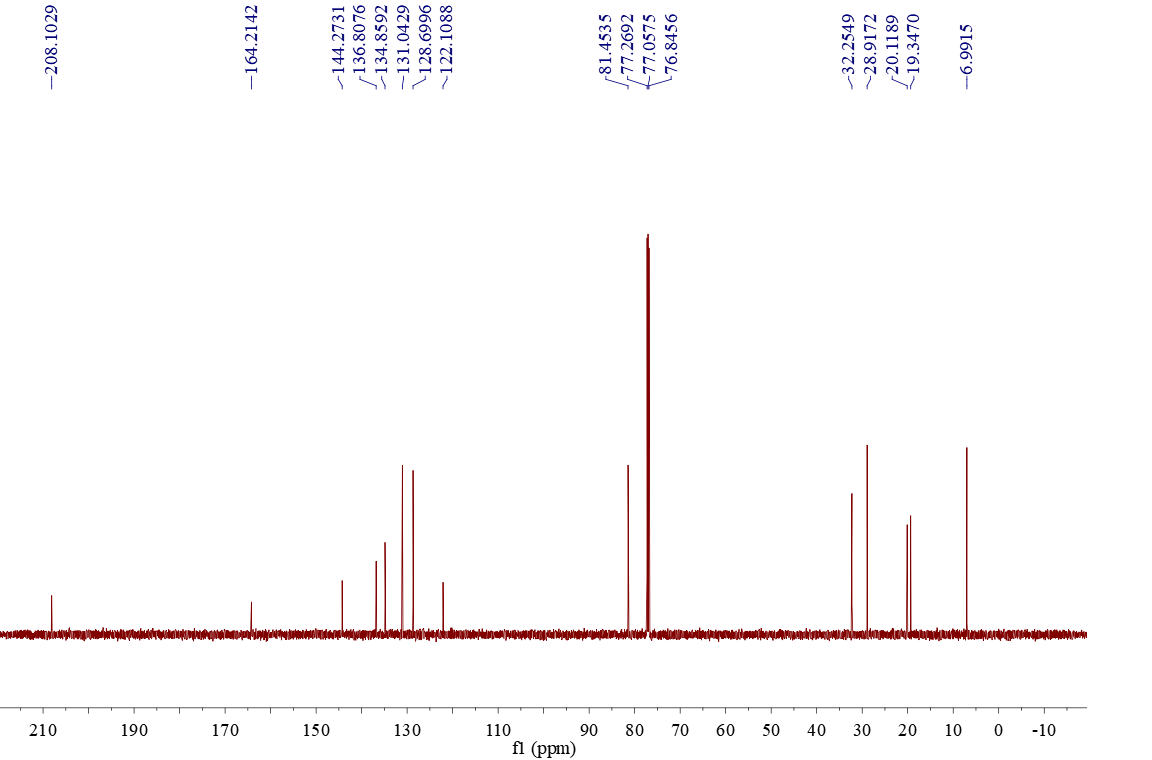
**

**Attached Fig.32** ^13^C NMR spectra of compound **2p**

**
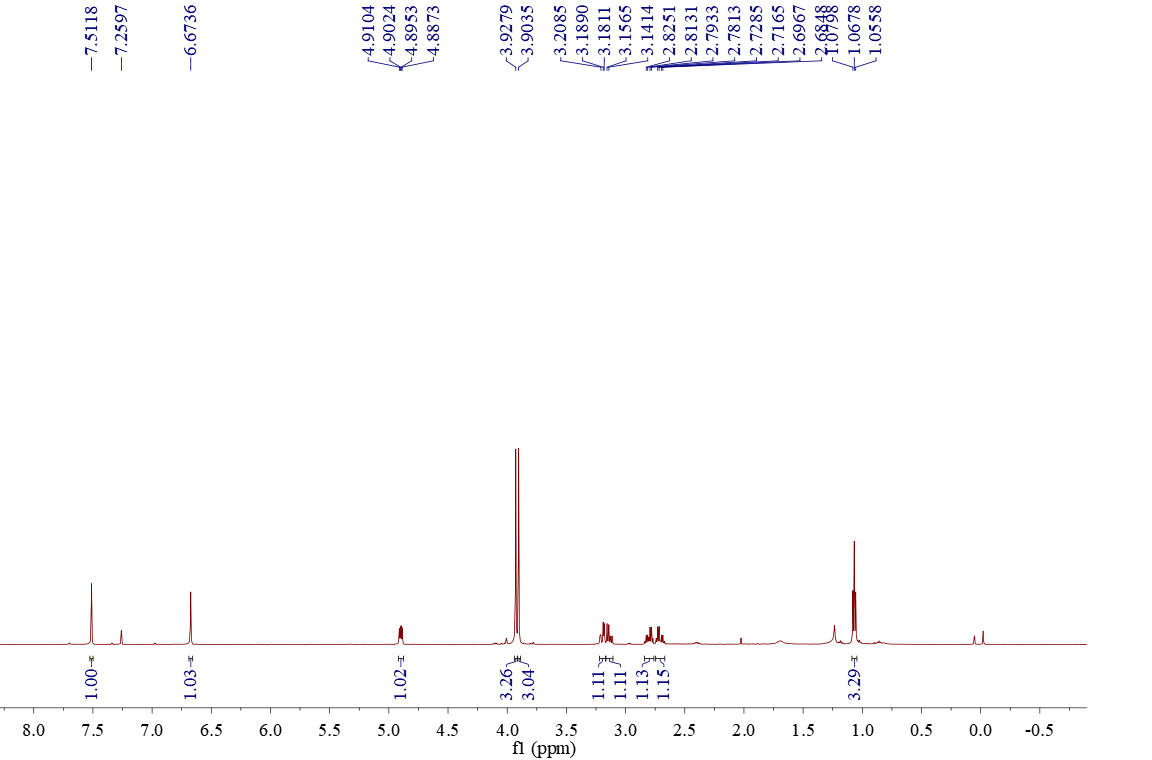
**

**Attached Fig.33** ^1^H NMR spectra of compound **2q**

**
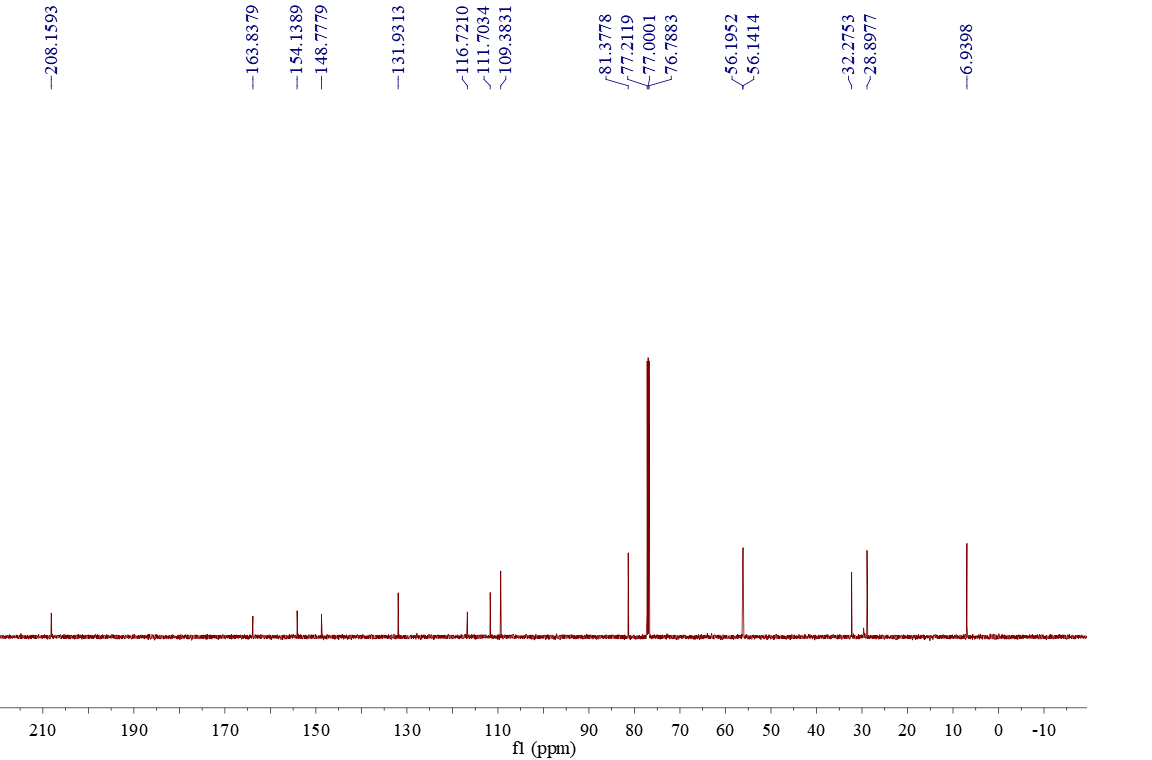
**

**Attached Fig.34** ^13^C NMR spectra of compound **2q**

**
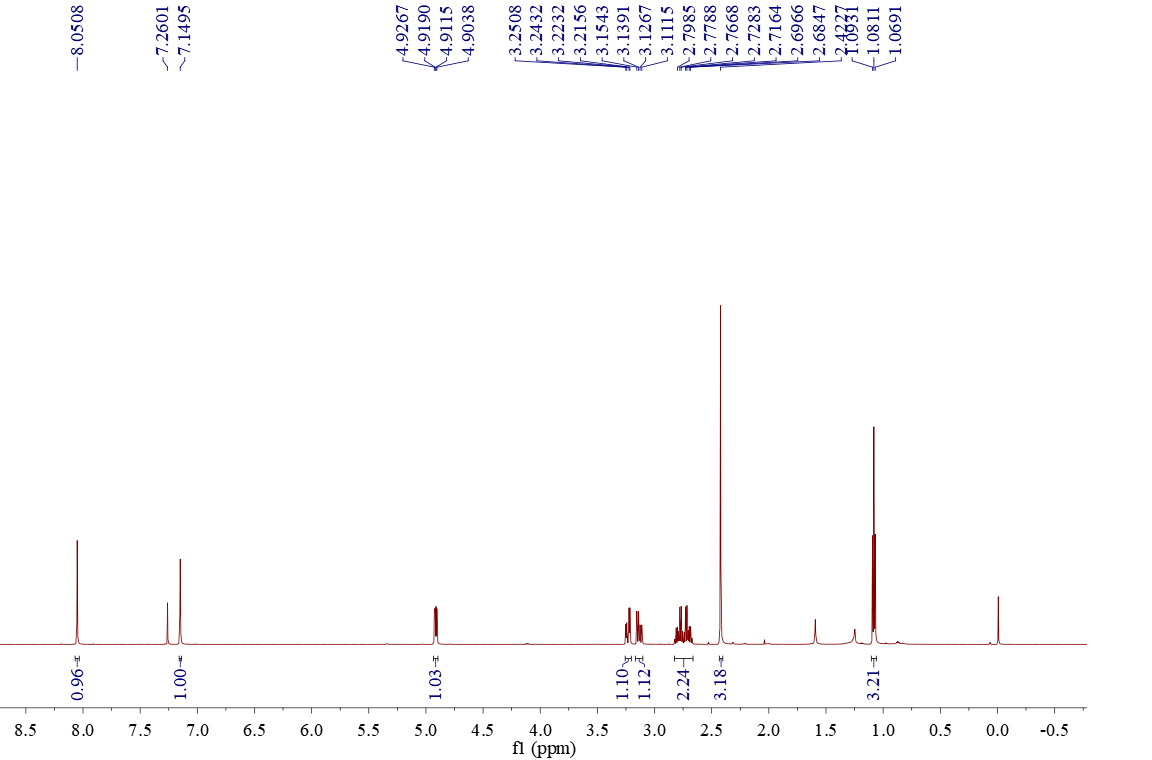
**

**Attached Fig.35** ^1^H NMR spectra of compound **2r**

**^13^C NMR spectra of compound 3r**

**
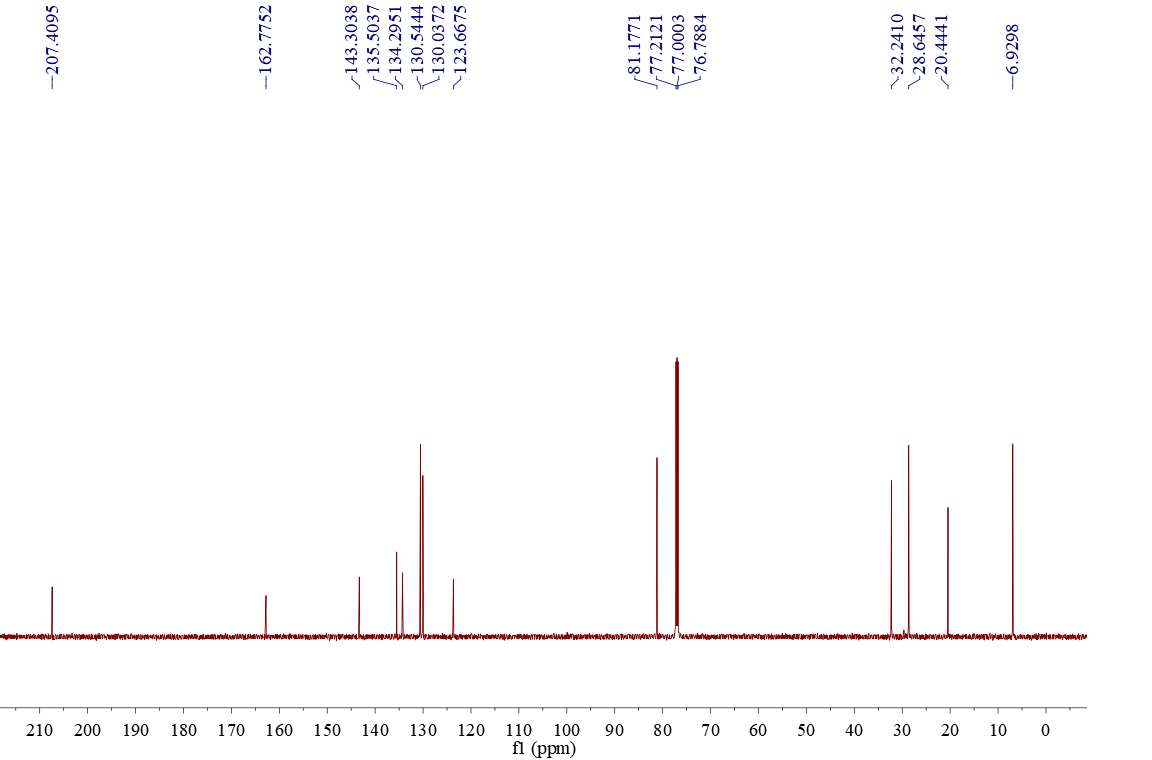
**

**Attached Fig.36** ^13^C NMR spectra of compound **2r**

**^1^H NMR spectra of compound 3s**

**
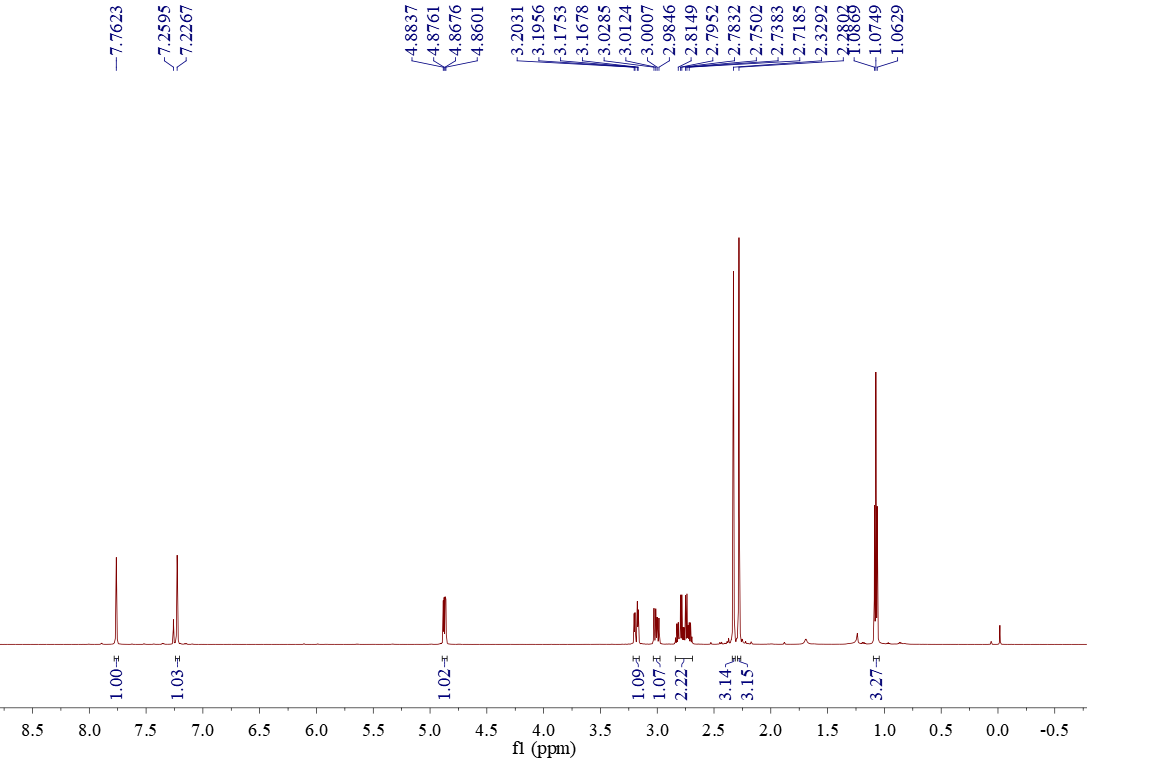
**

**Attached Fig.37** ^1^H NMR spectra of compound **2s**

**
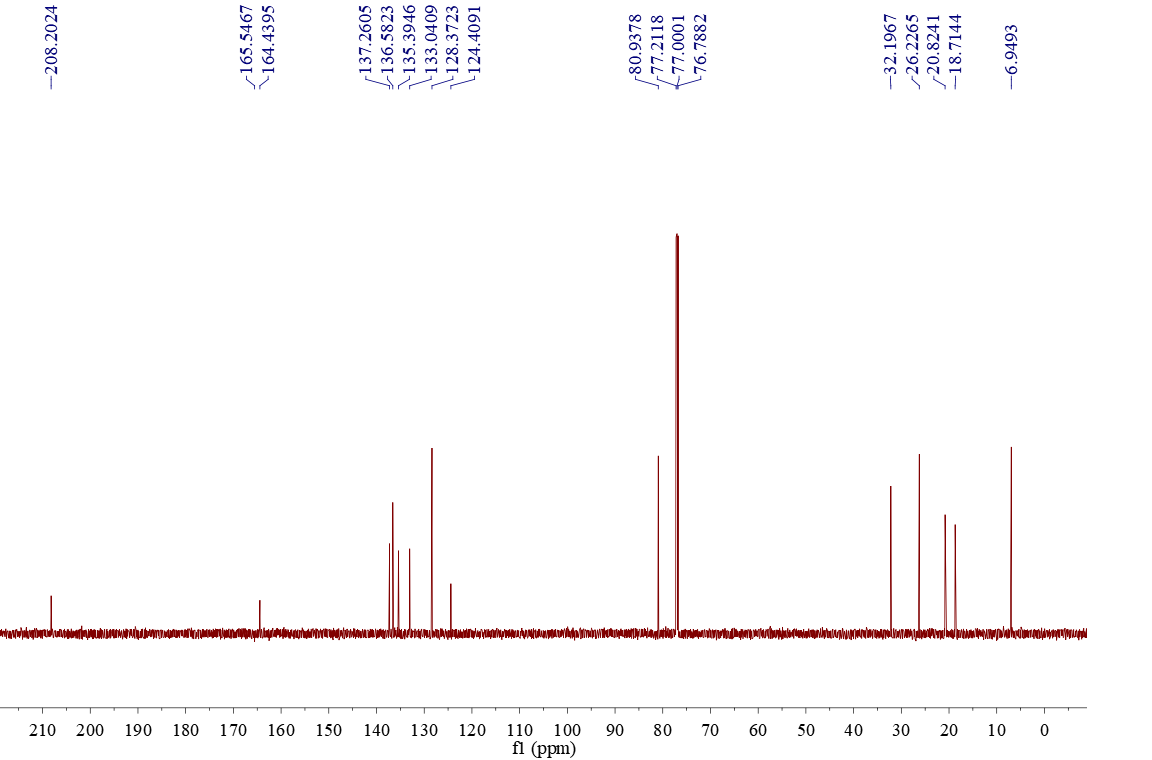
**

**Attached Fig.38** ^13^C NMR spectra of compound **2s**

**
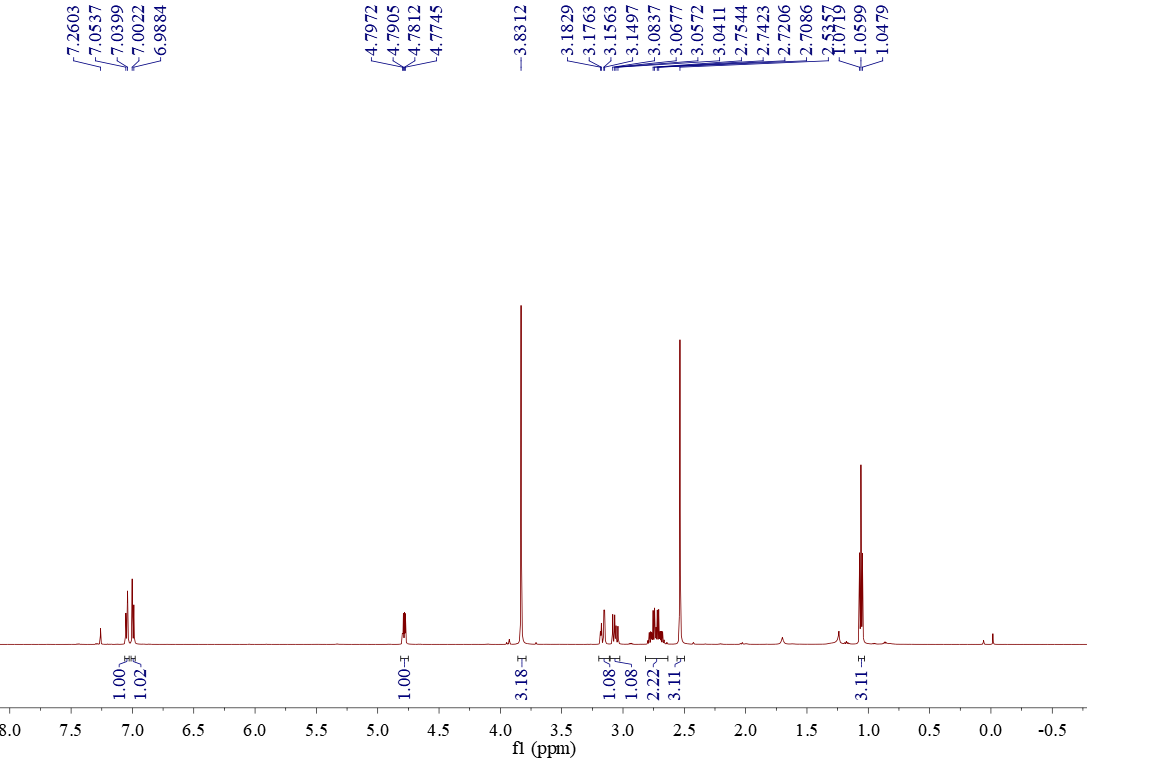
**

**Attached Fig.41** ^1^H NMR spectra of compound **2t**

**
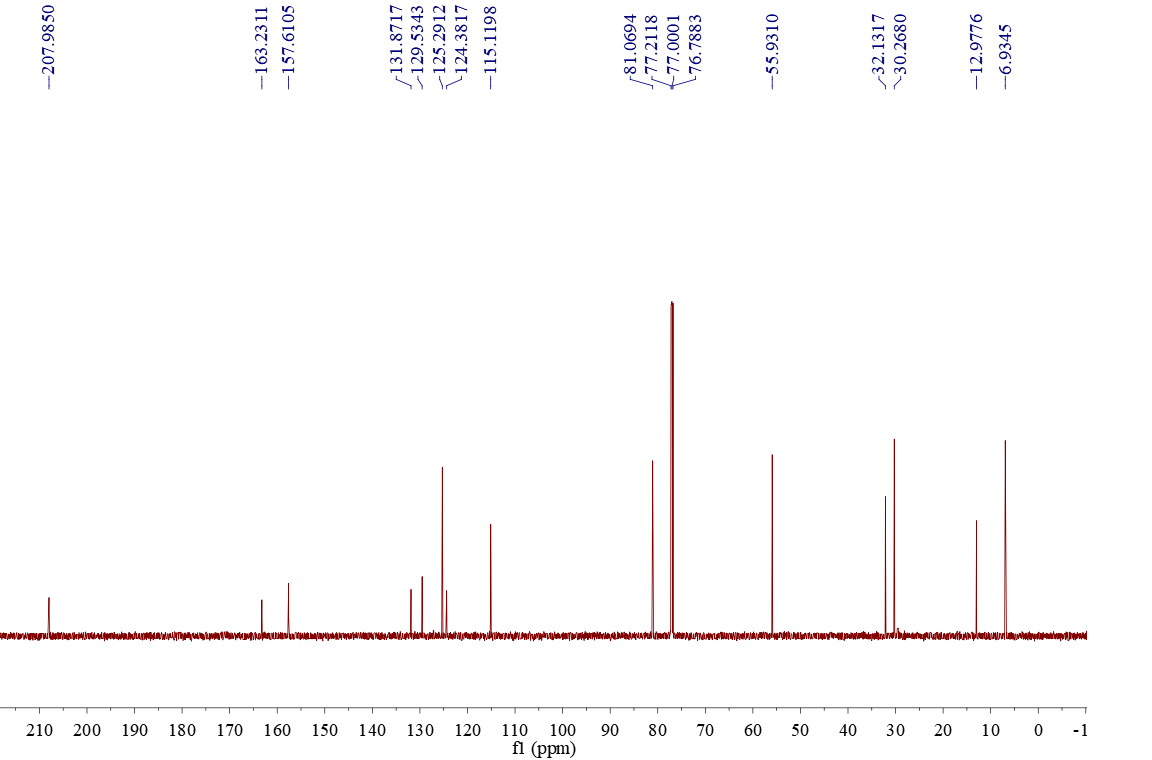
**

**Attached Fig.42** ^13^C NMR spectra of compound **2t**

**^
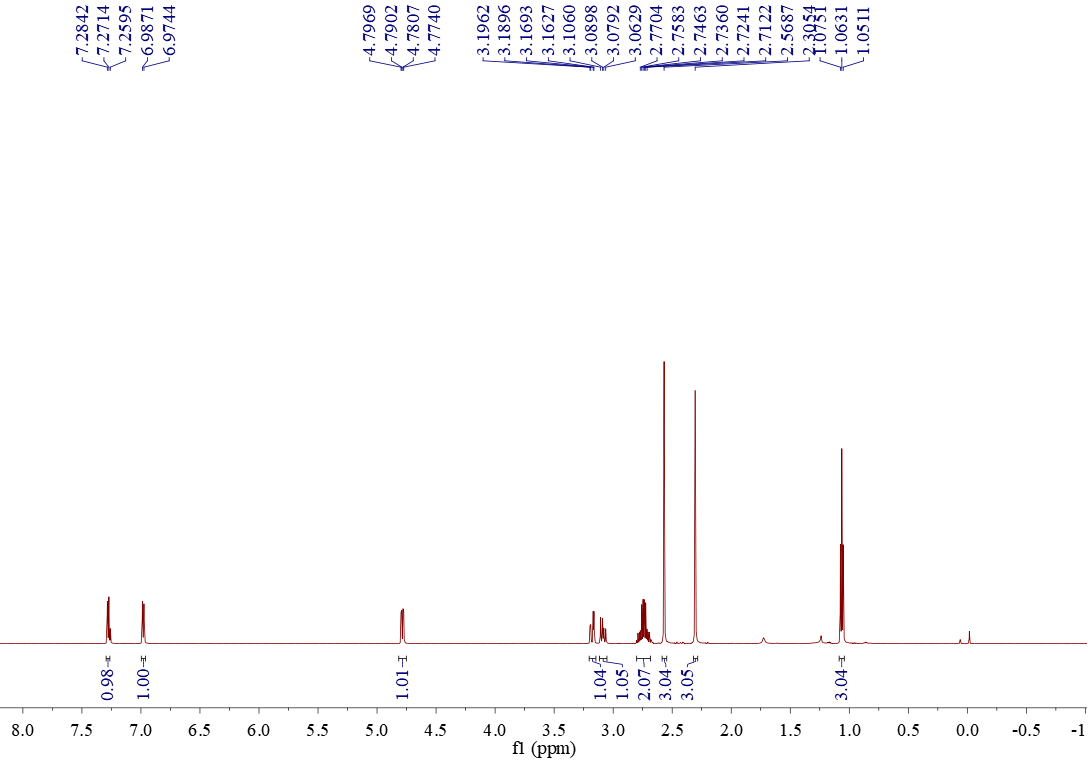
^**

**Attached Fig.43** ^1^H NMR spectra of compound **2u**

**^
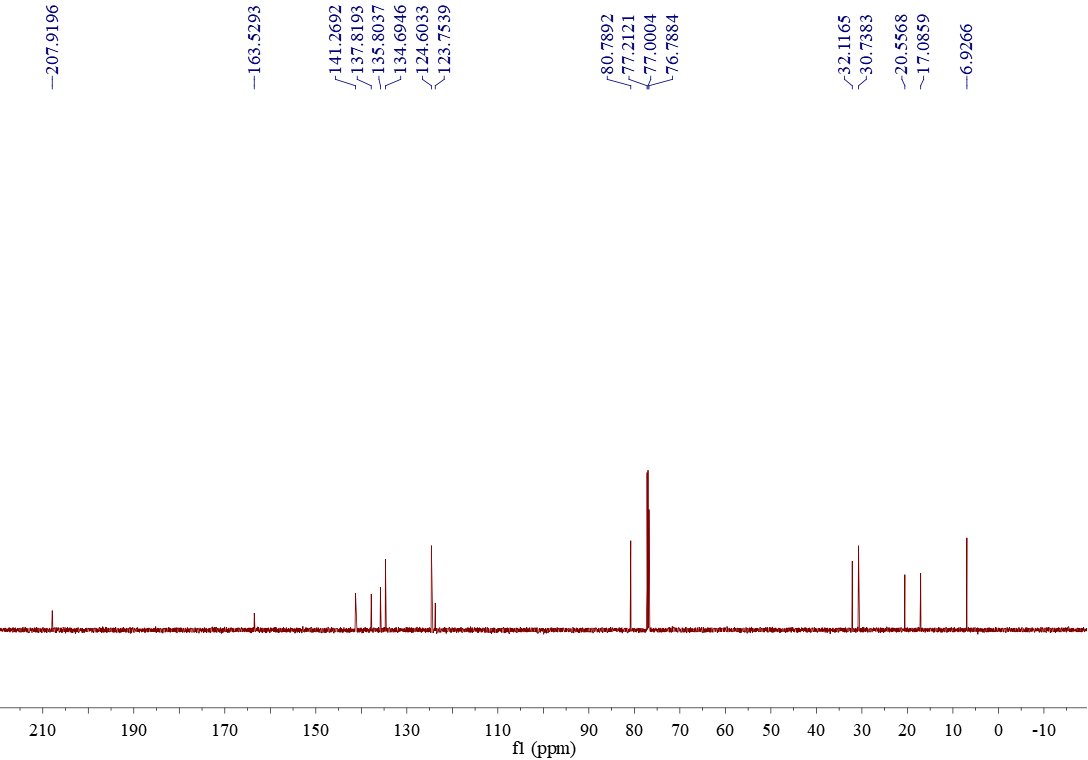
^**

**Attached Fig.44** ^13^C NMR spectra of compound **2u**

**
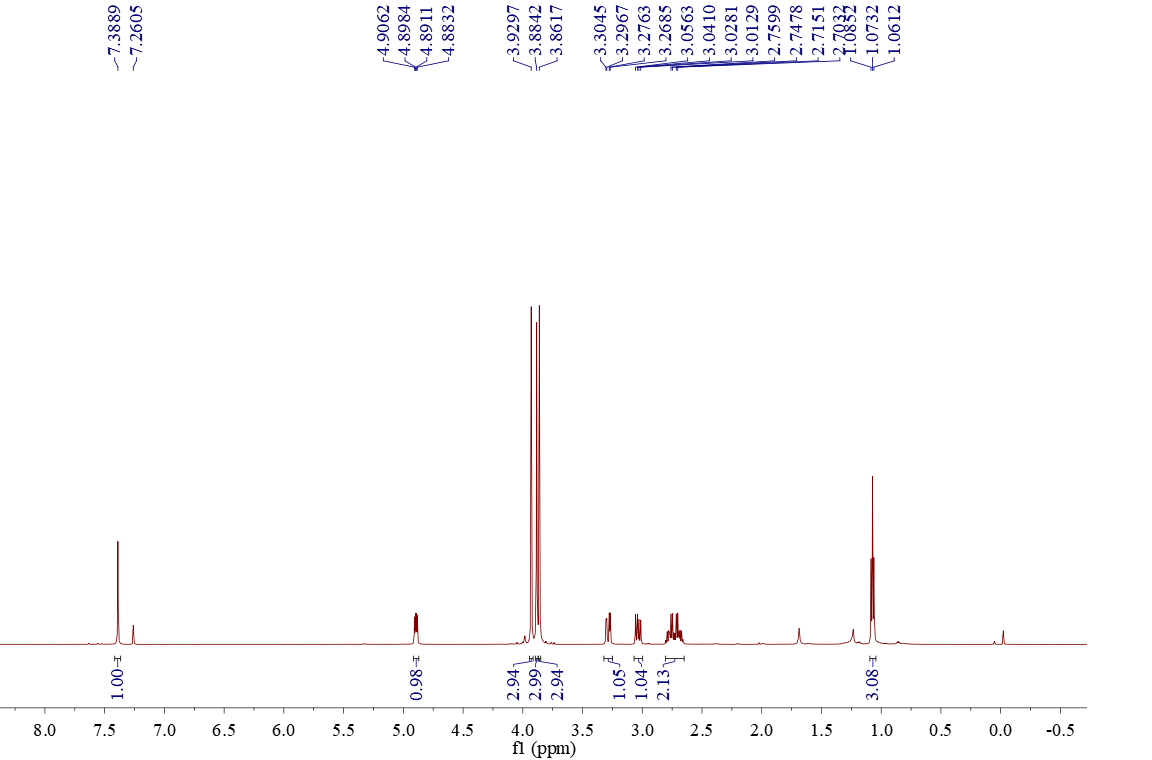
**

**Attached Fig.39** ^1^H NMR spectra of compound **2v**

**
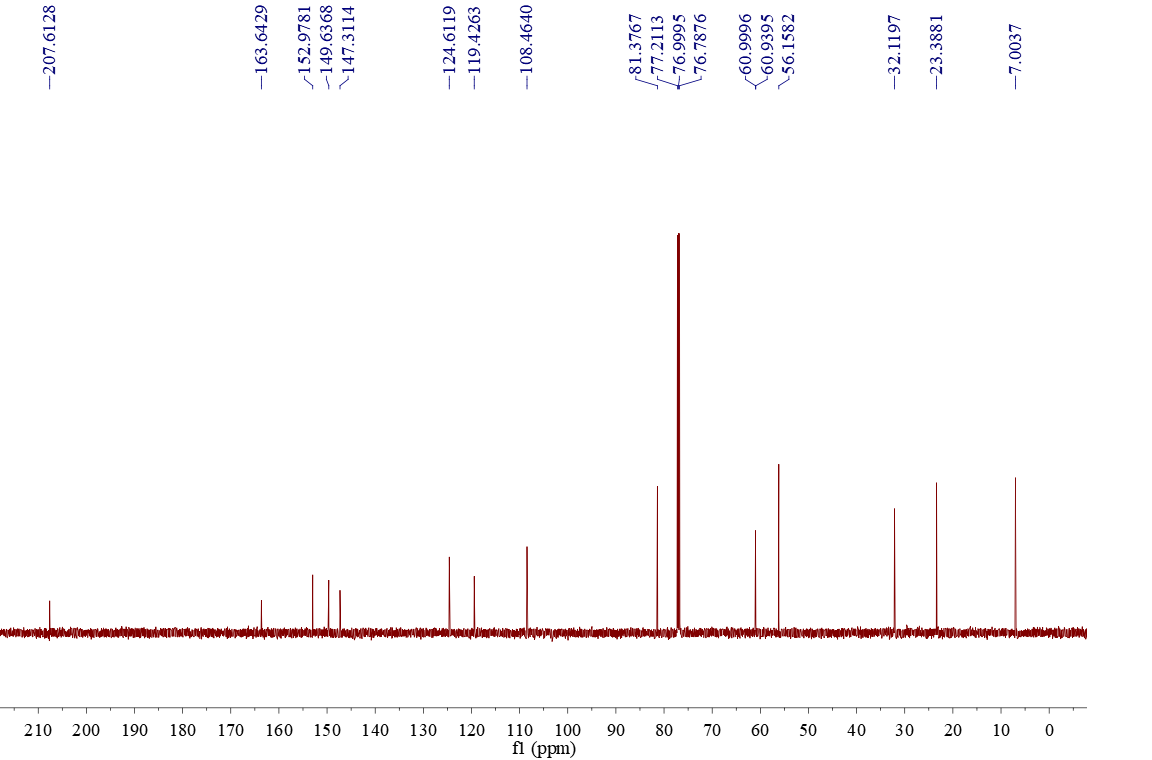
**

**Attached Fig.40** ^13^C NMR spectra of compound **2v**

**
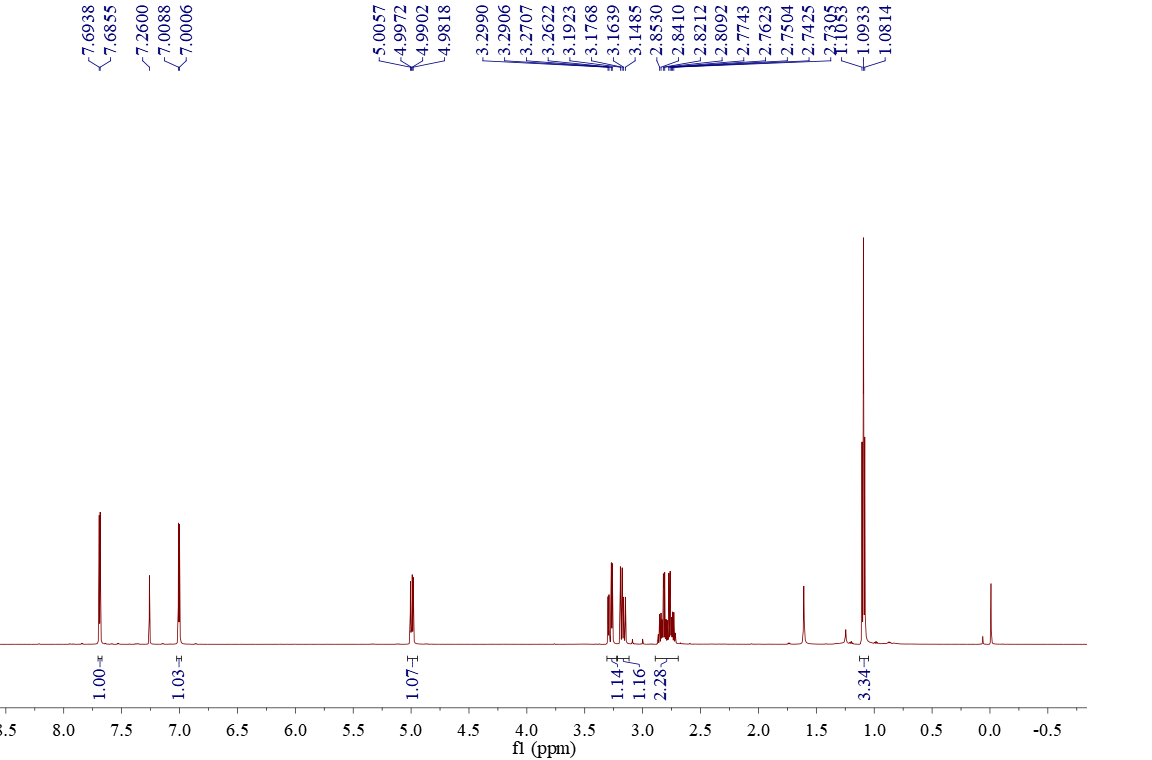
**

**Attached Fig.45** ^1^H NMR spectra of compound **2w**

**
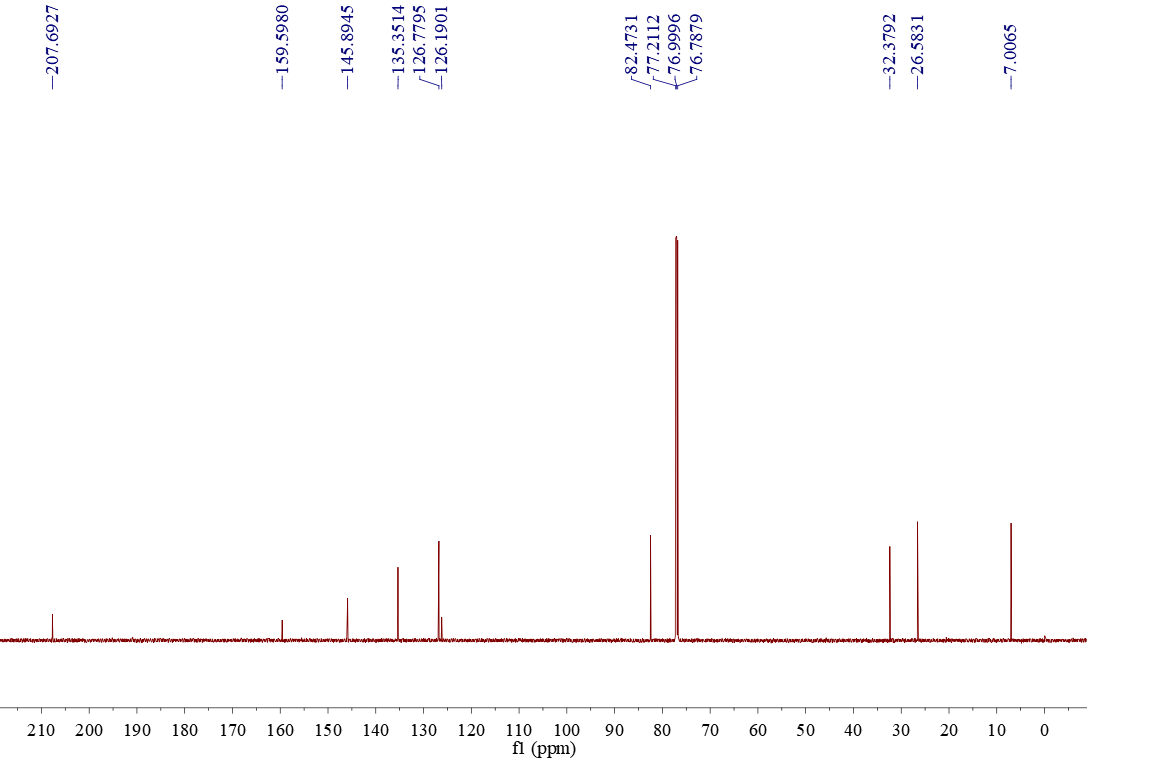
**

**Attached Fig.46** ^13^C NMR spectra of compound **2w**

**
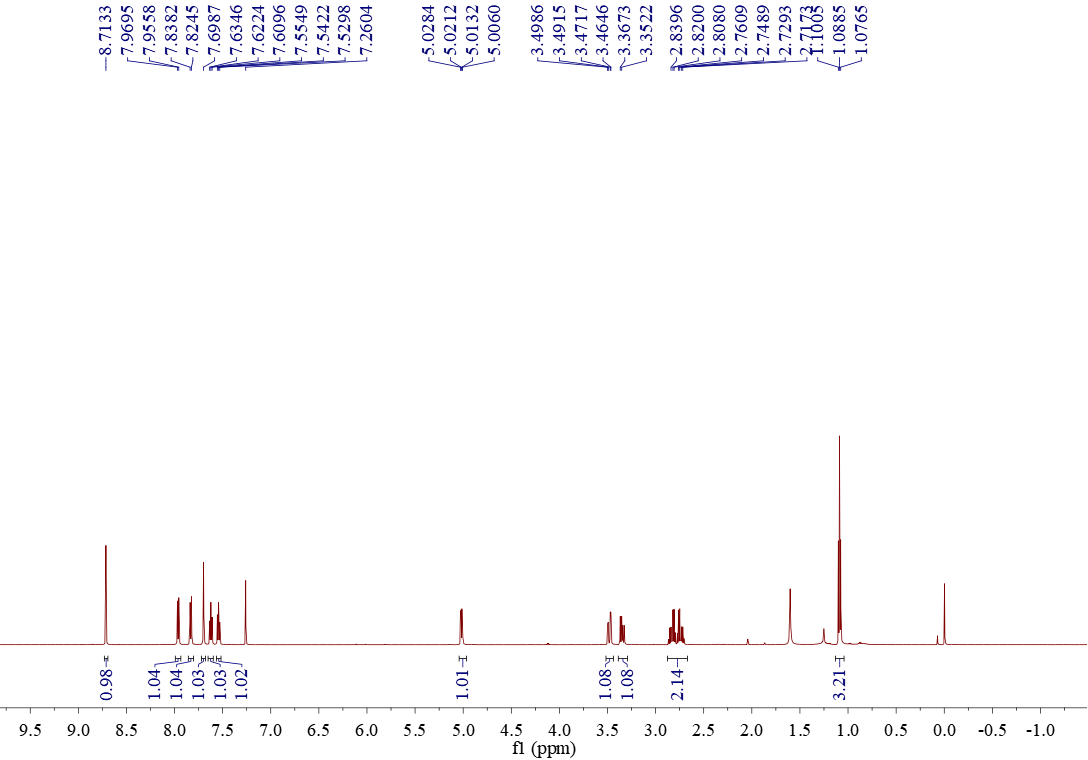
**

**Attached Fig.47** ^1^H NMR spectra of compound **2x**

**
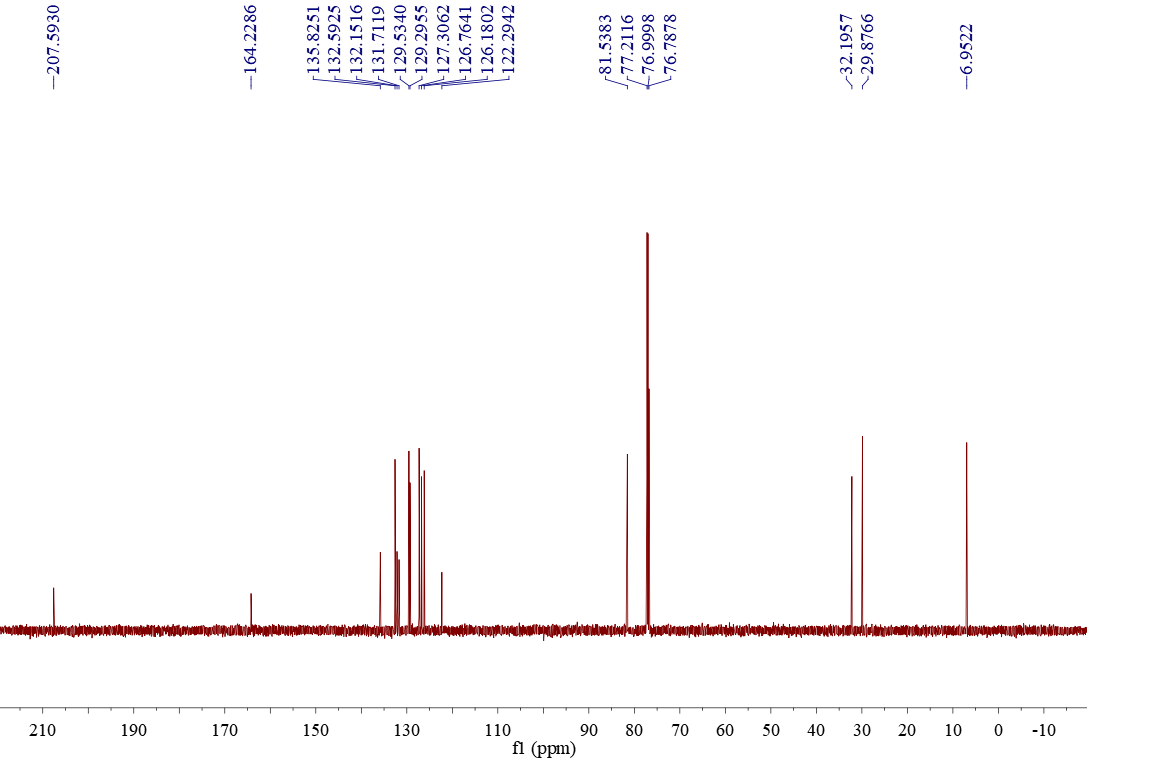
**

**Attached Fig.48** ^13^C NMR spectra of compound **2x**

**^
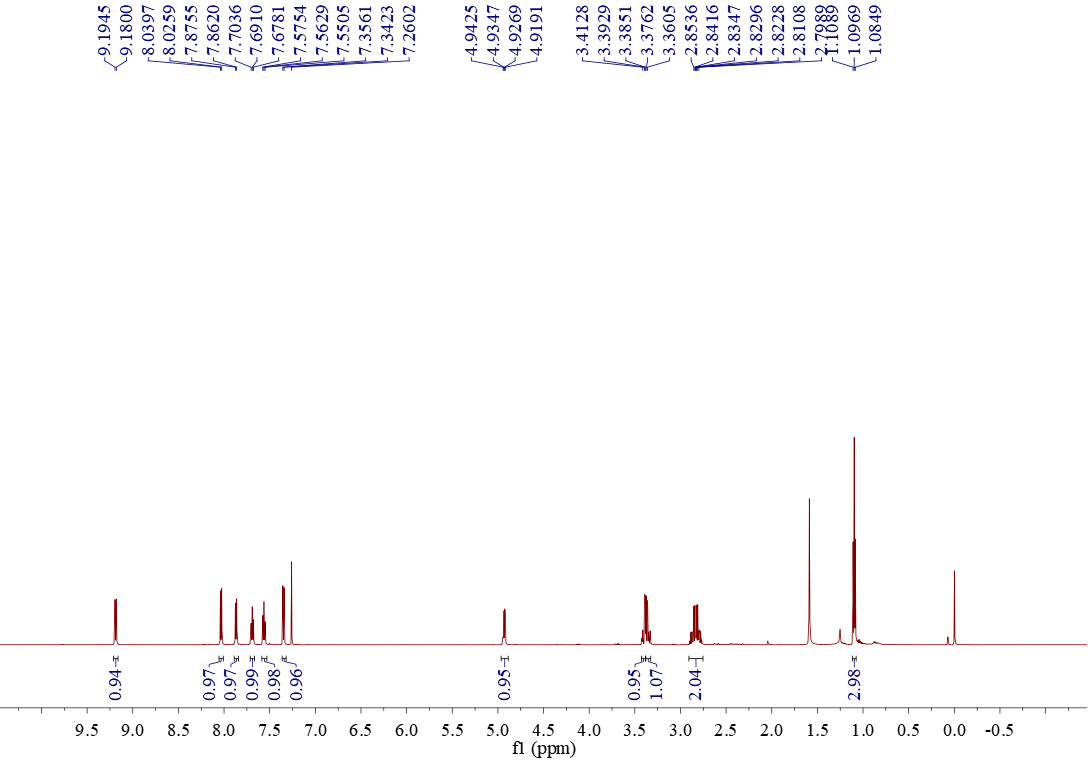
^**

**Attached Fig.49** ^1^H NMR spectra of compound **2y**

**
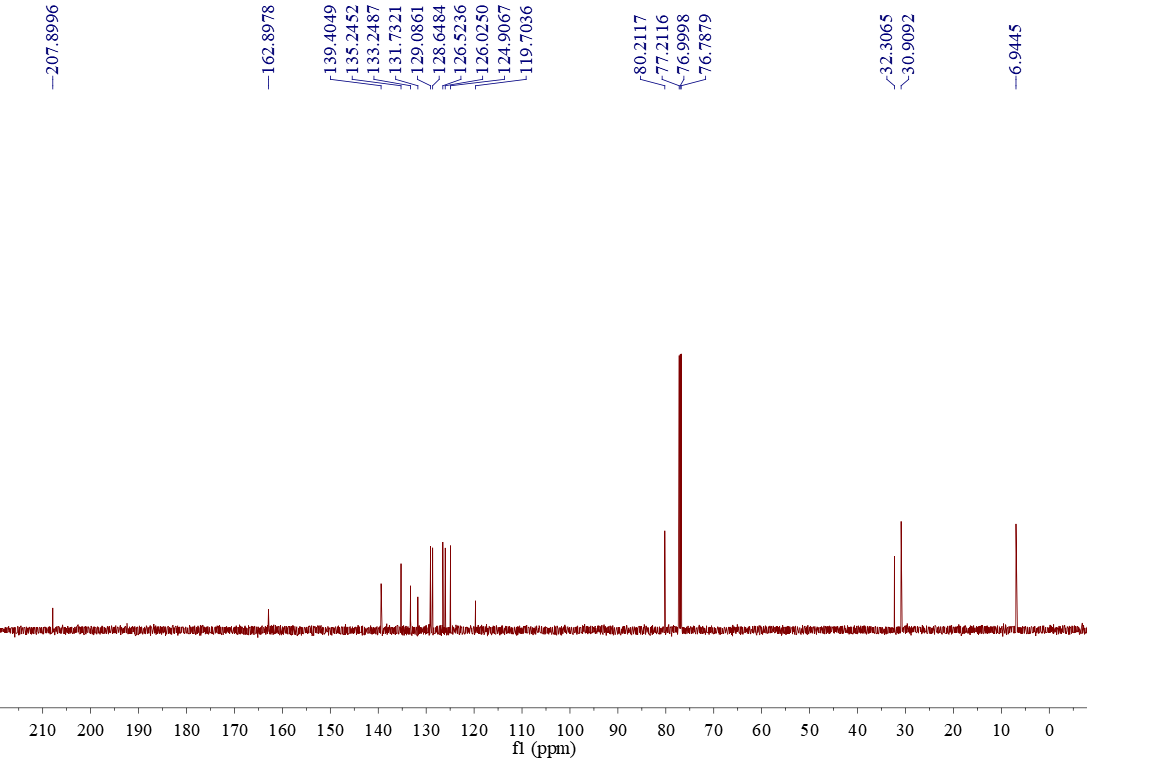
**

**Attached Fig.50** ^13^C NMR spectra of compound **2y**

**
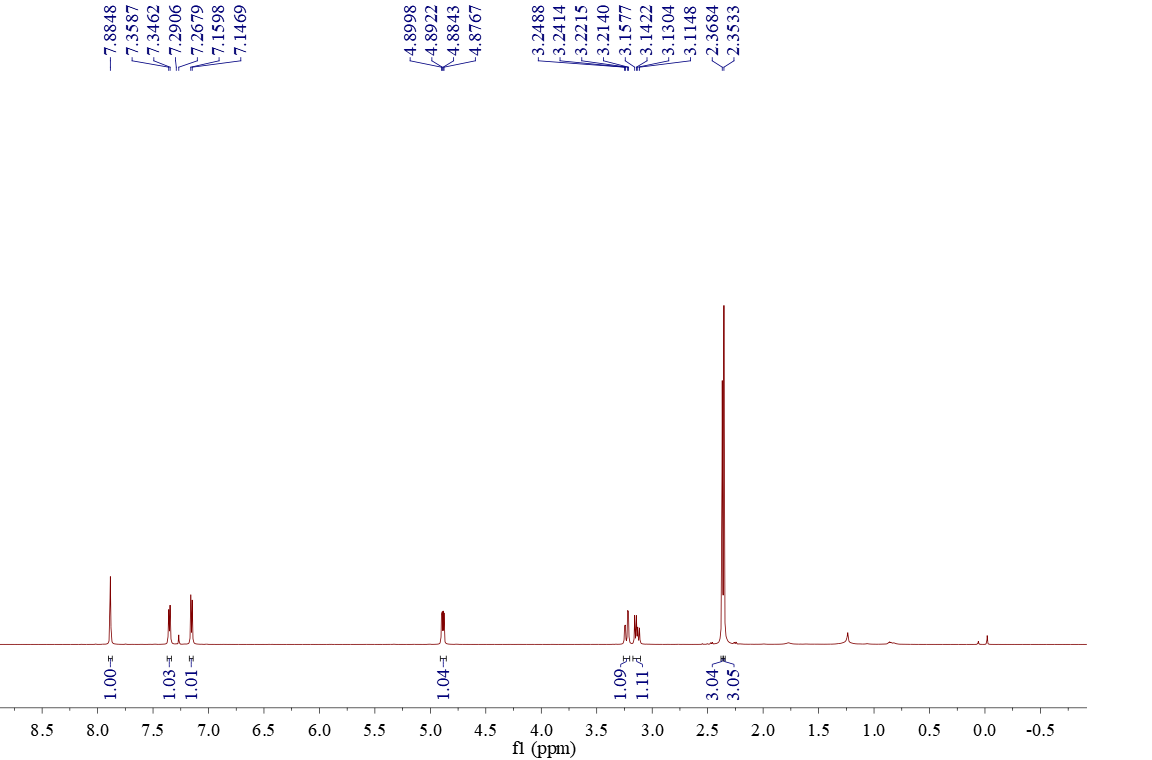
**

**Attached Fig.51** ^1^H NMR spectra of compound **2z**

**^
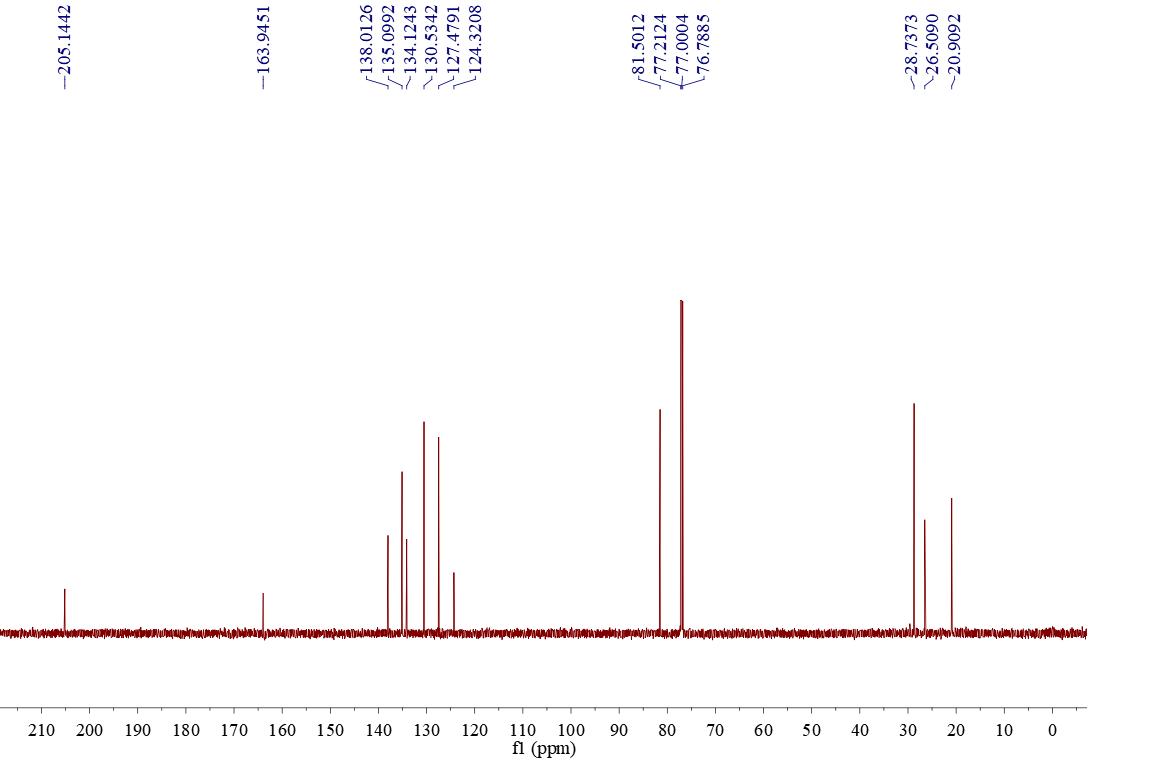
^**

**Attached Fig.52** ^13^C NMR spectra of compound **2z**

**^
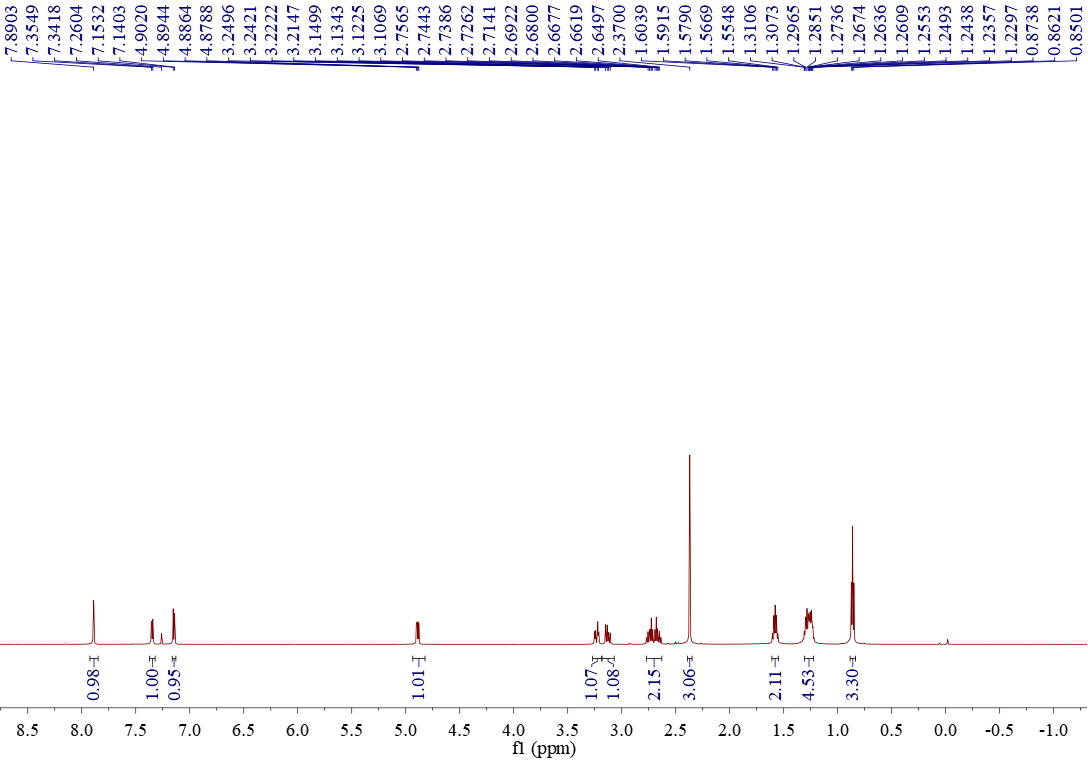
^**

**Attached Fig.53** ^1^H NMR spectra of compound **2aa**

**
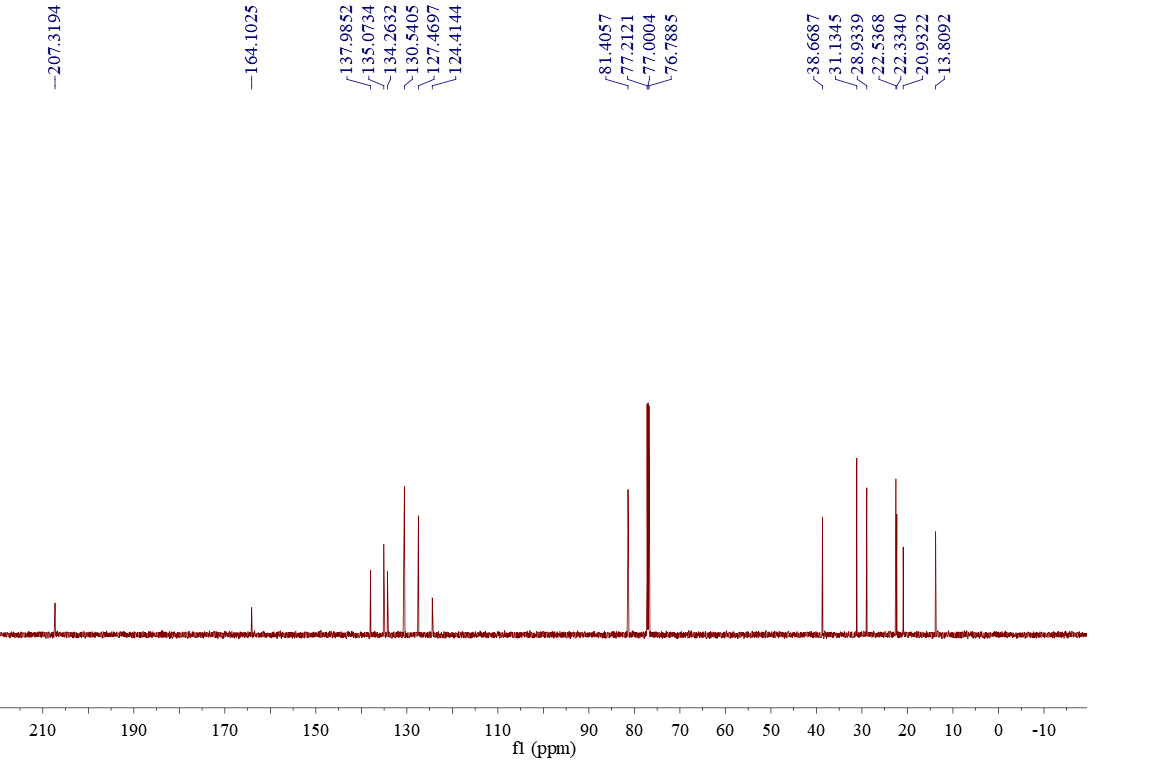
**

**Attached Fig.54** ^13^C NMR spectra of compound **2aa**


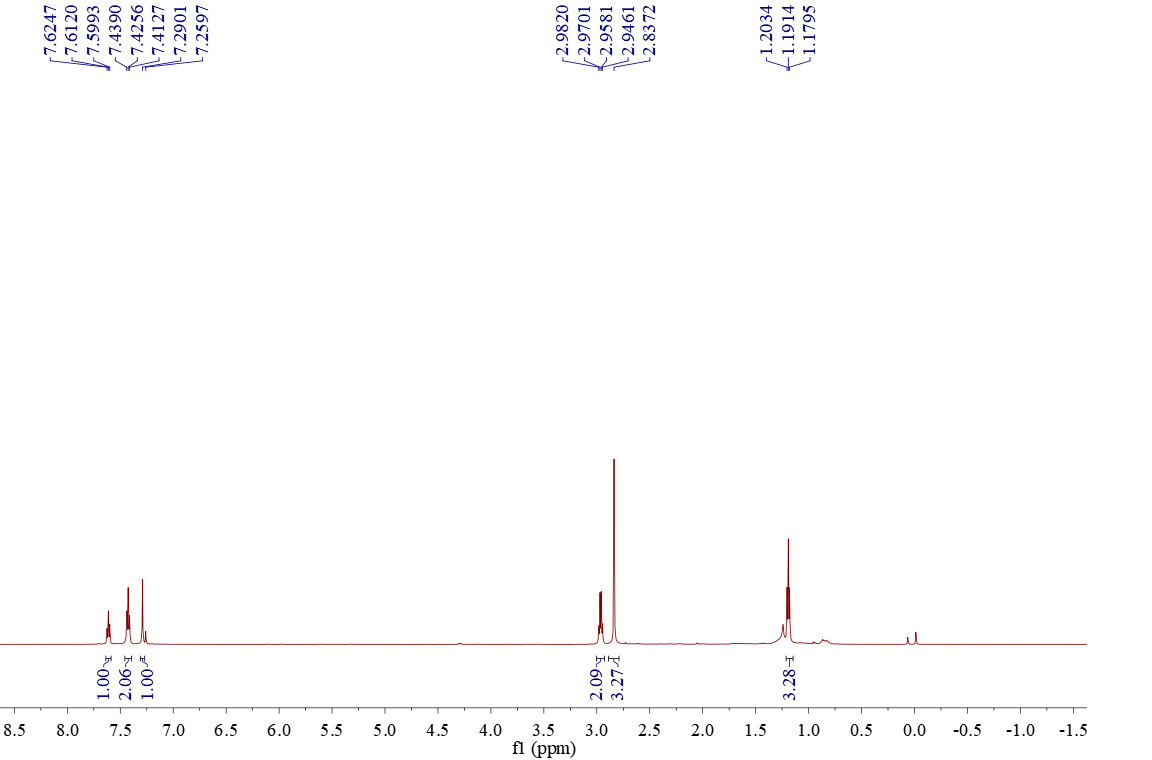


**Attached Fig.55** ^1^H NMR spectra of compound **3a**

**
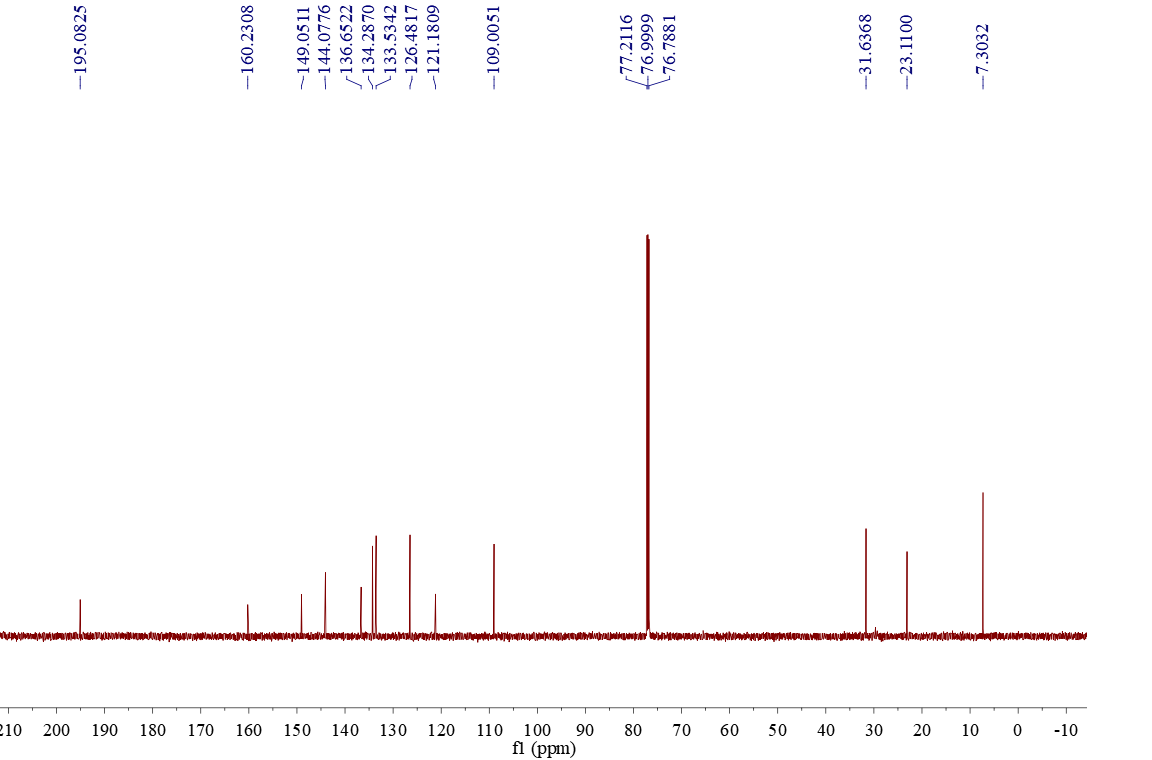
**

**Attached Fig.56** ^13^C NMR spectra of compound **3a**

**
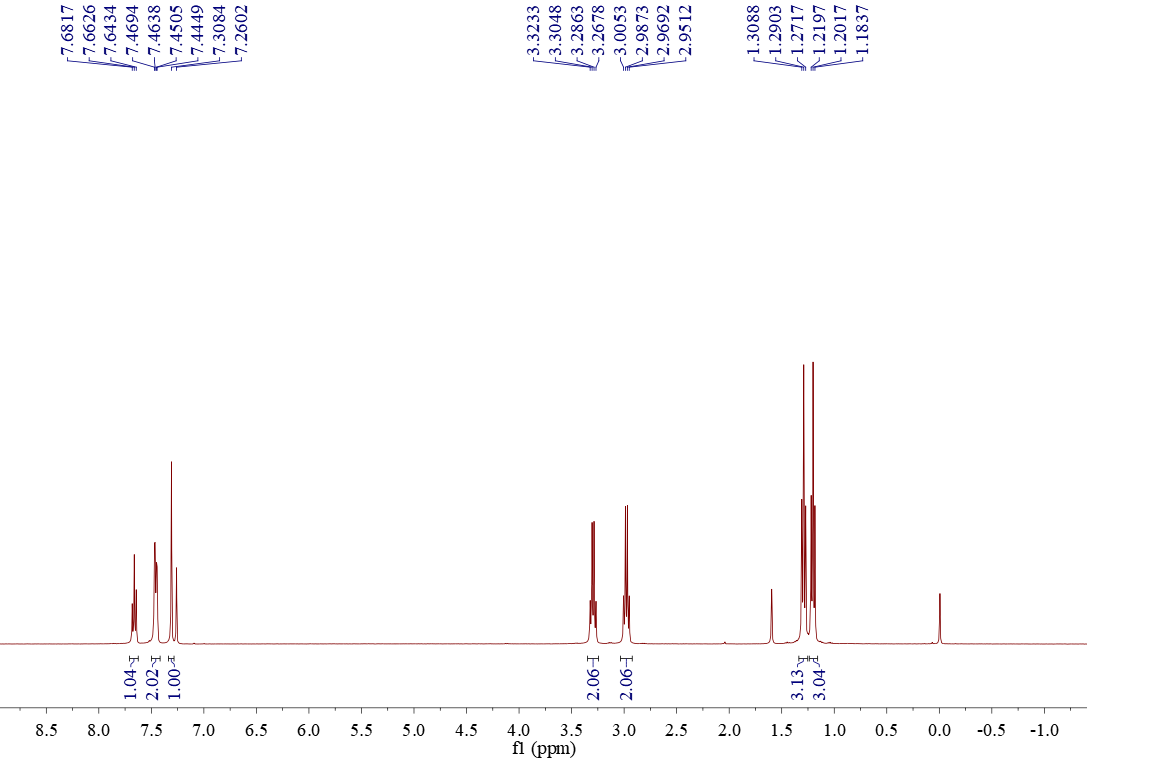
**

**Attached Fig.57** ^1^H NMR spectra of compound **3b**

**
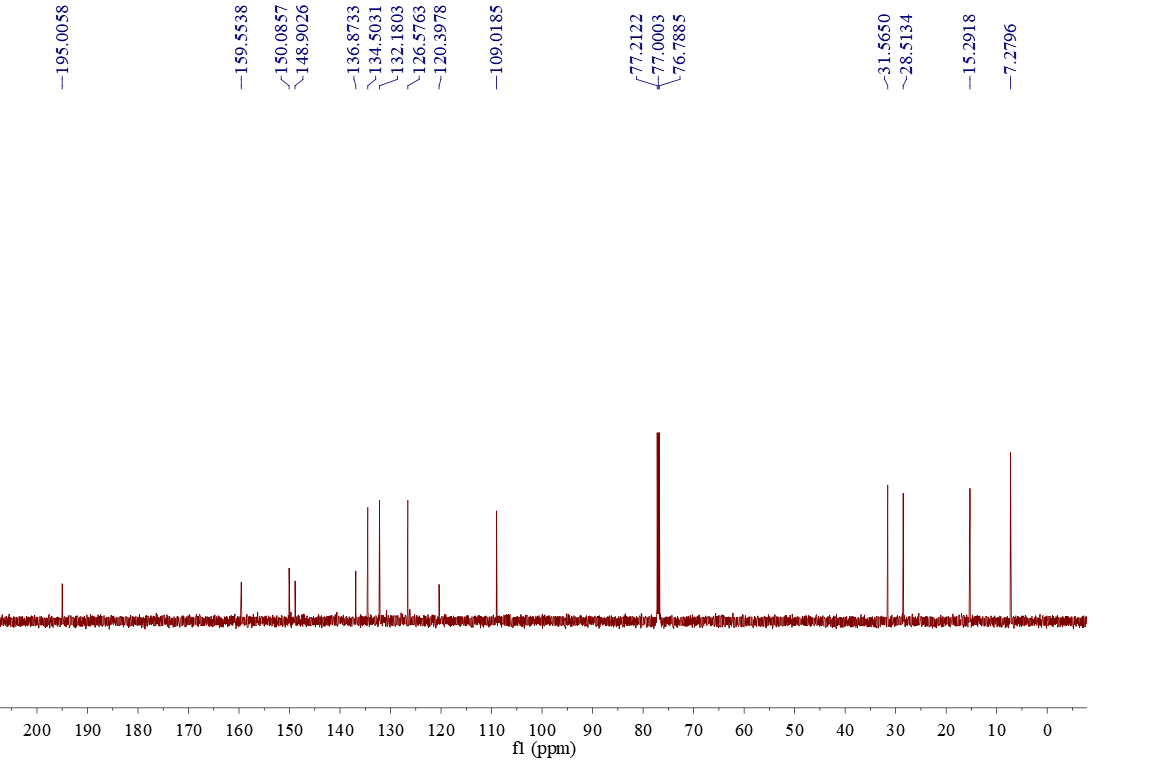
**

**Attached Fig.58** ^13^C NMR spectra of compound **3b**

**
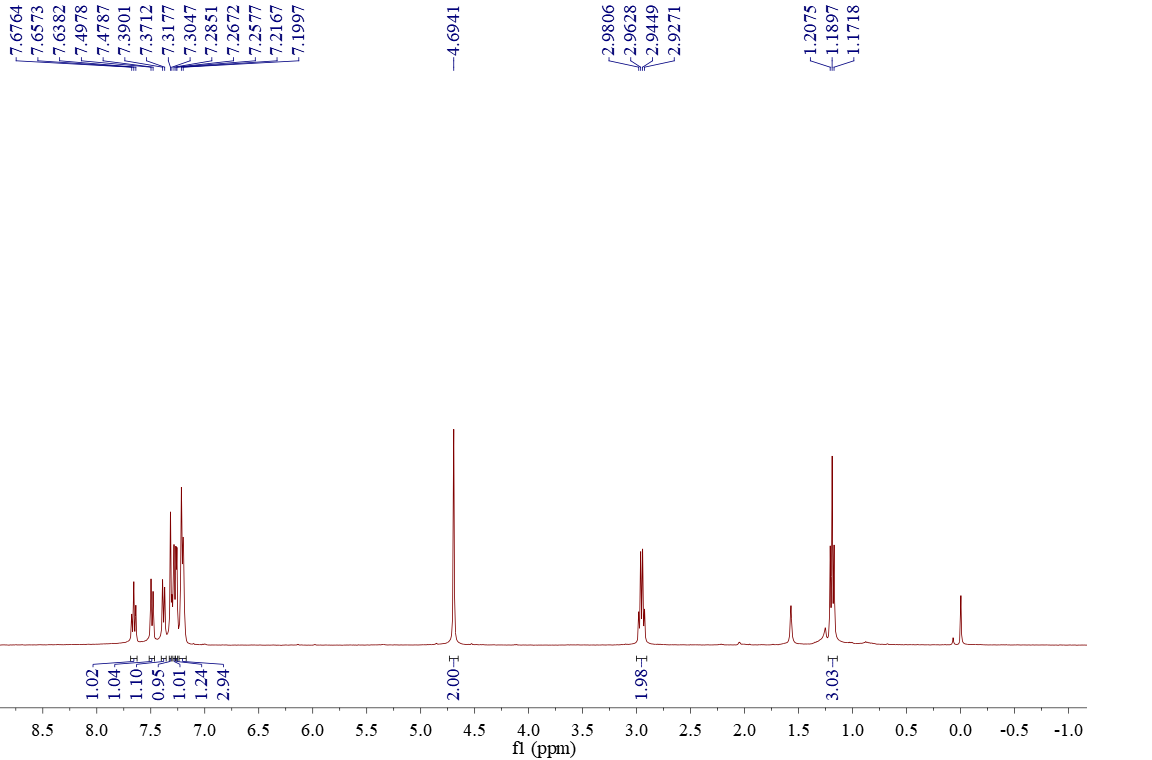
**

**Attached Fig.59** ^1^H NMR spectra of compound **3c**

**
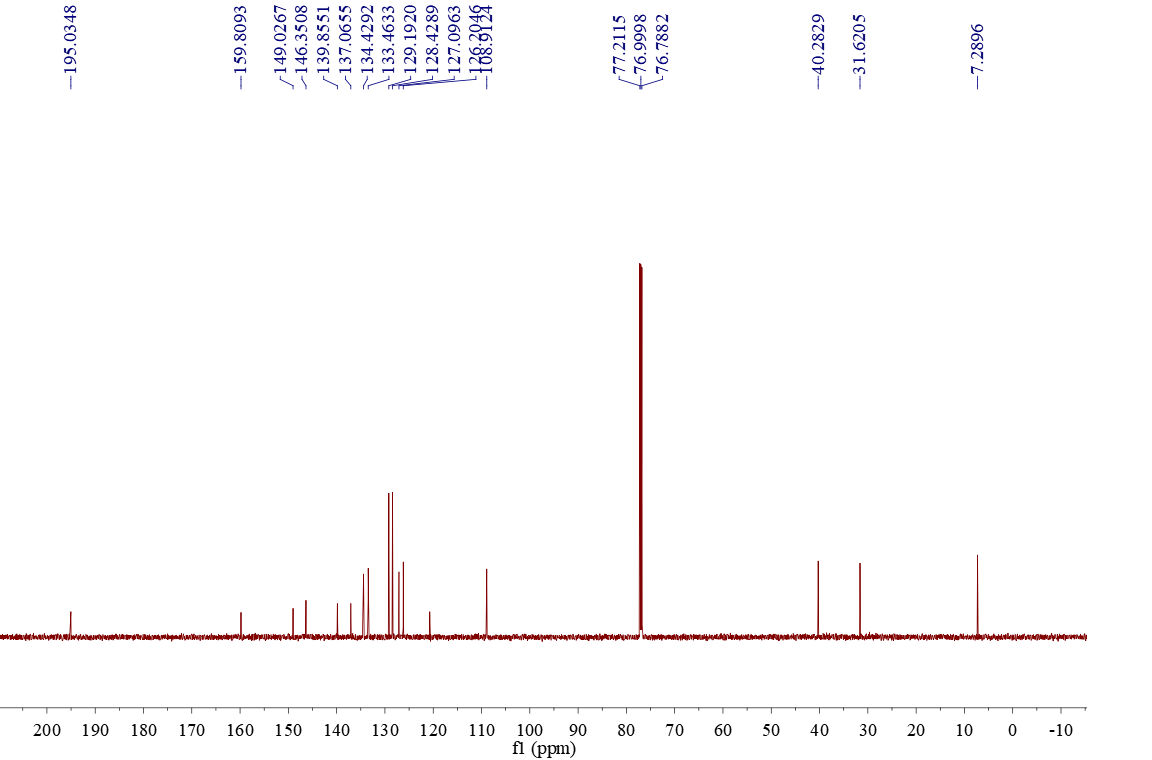
**

**Attached Fig.60** ^13^C NMR spectra of compound **3c**

**^
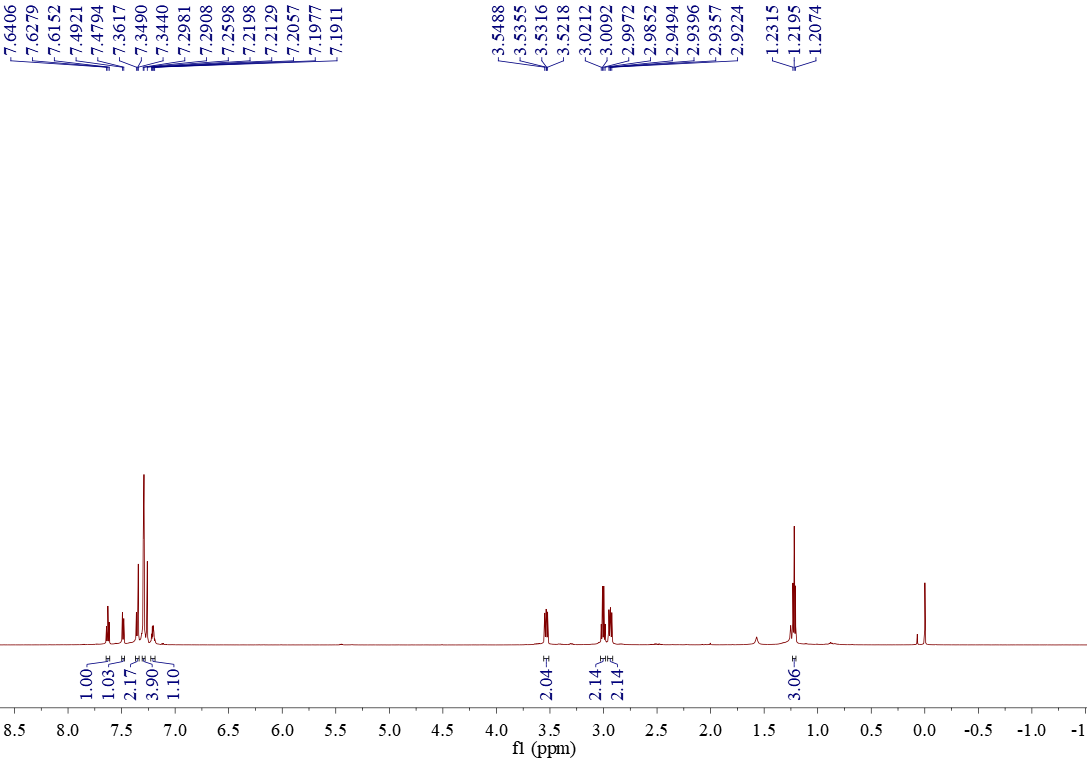
^**

**Attached Fig.61** ^1^H NMR spectra of compound **3d**

**^13^C NMR spectra of compound 4d**

**
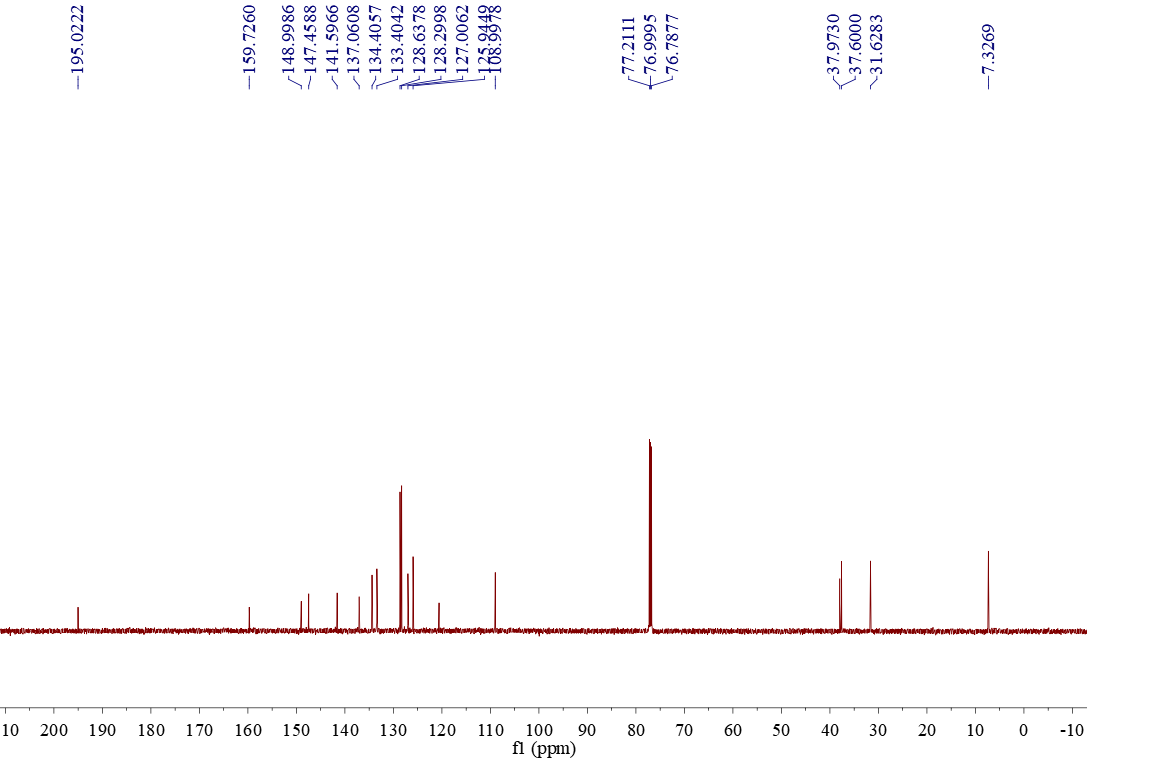
**

**Attached Fig.62** ^13^C NMR spectra of compound **3d**

**
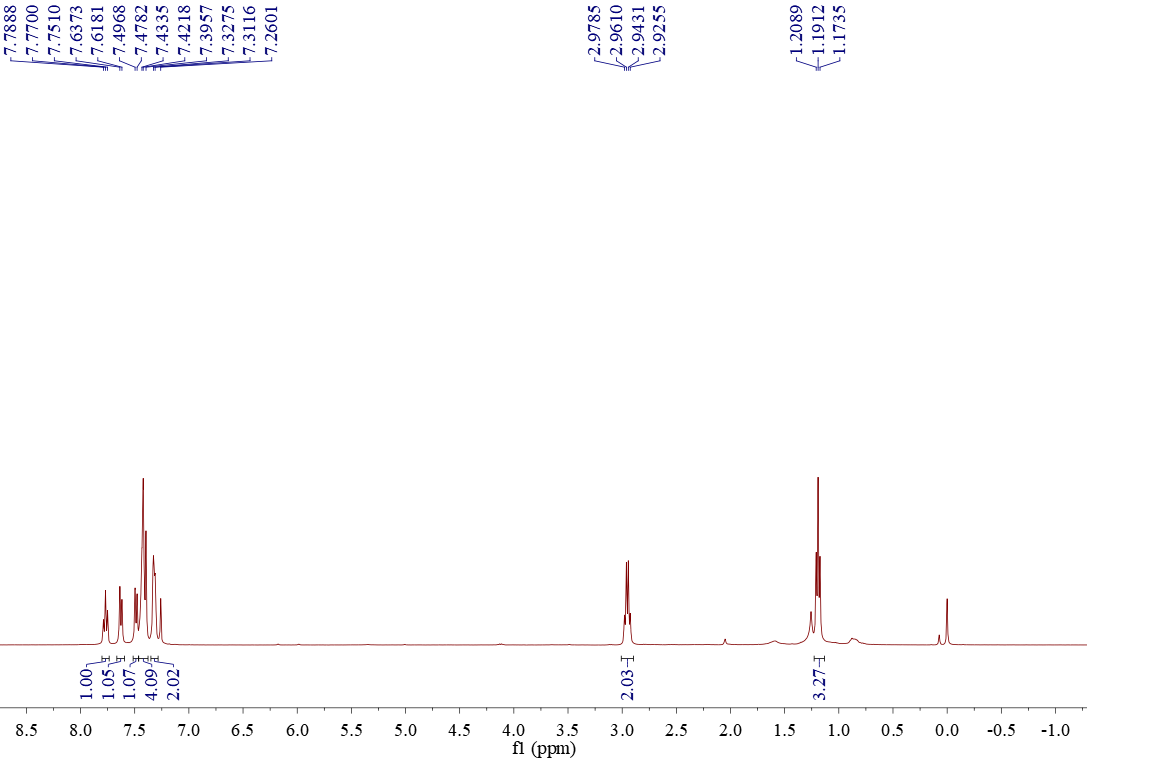
**

**Attached Fig.63** ^1^H NMR spectra of compound **3e**

**^13^C NMR spectra of compound 4e**

**
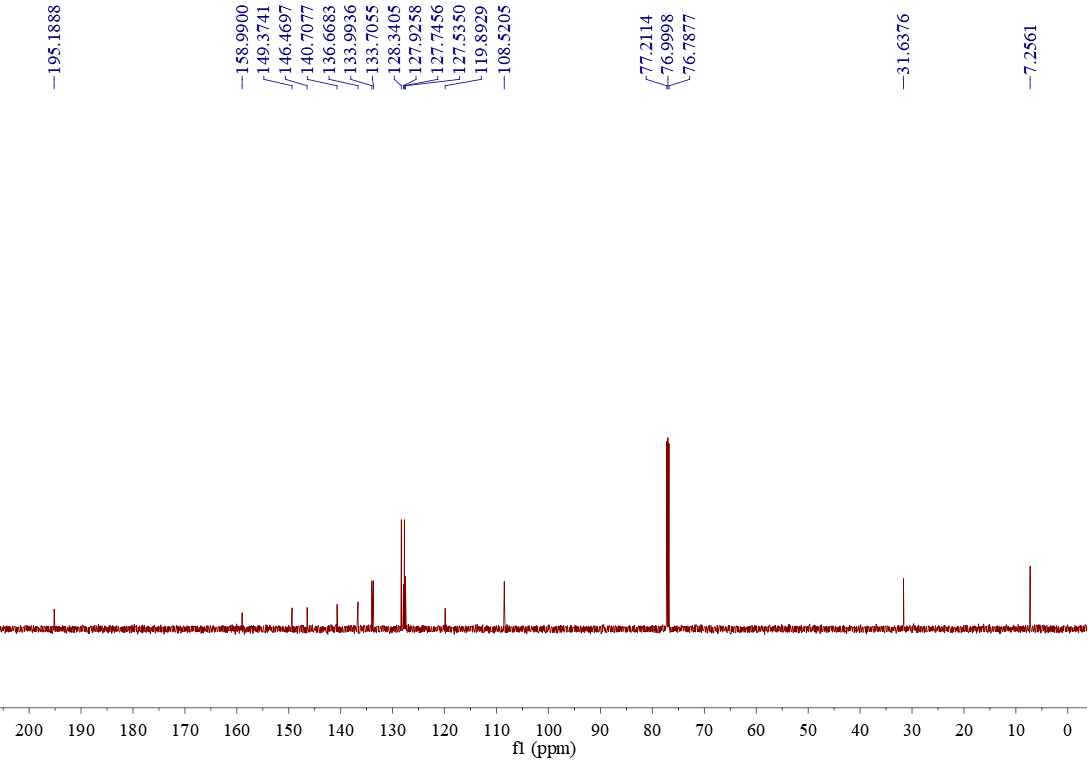
**

**Attached Fig.64** ^13^C NMR spectra of compound **3e**

**
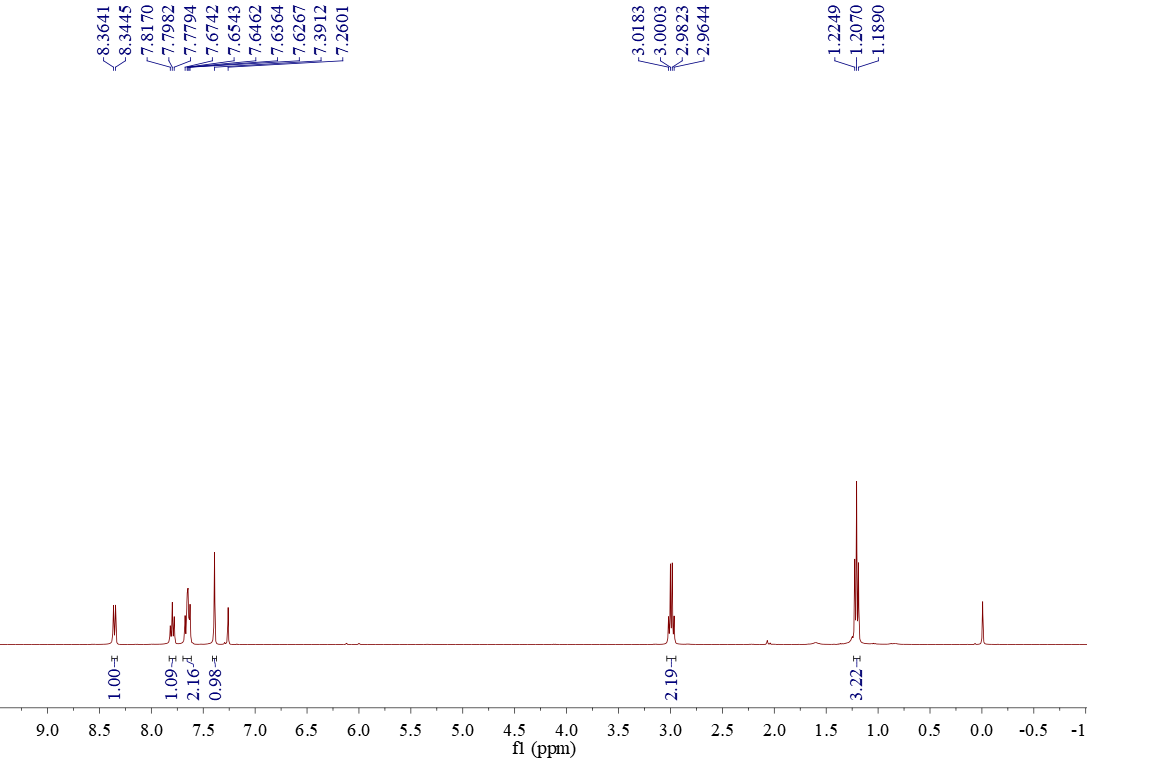
**

**Attached Fig.65** ^1^H NMR spectra of compound **3f**


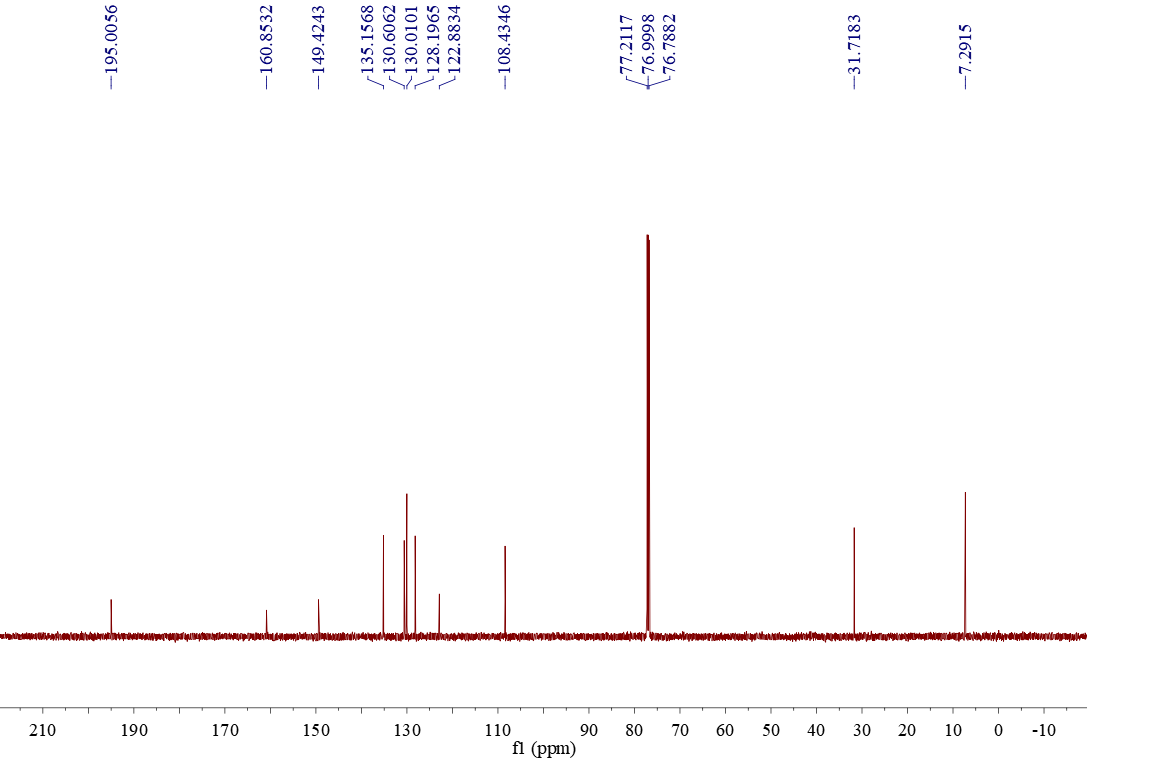


**Attached Fig.66** ^13^C NMR spectra of compound **3f**

**
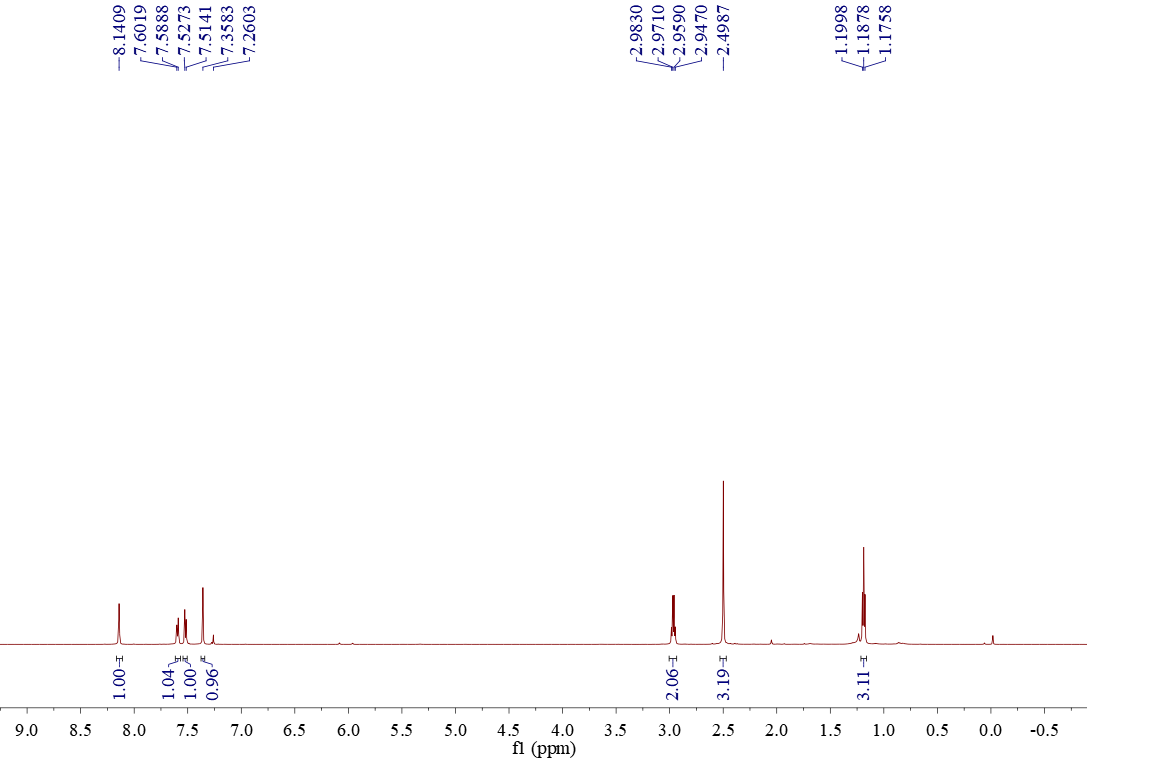
**

**Attached Fig.67** ^1^H NMR spectra of compound **3g**

**
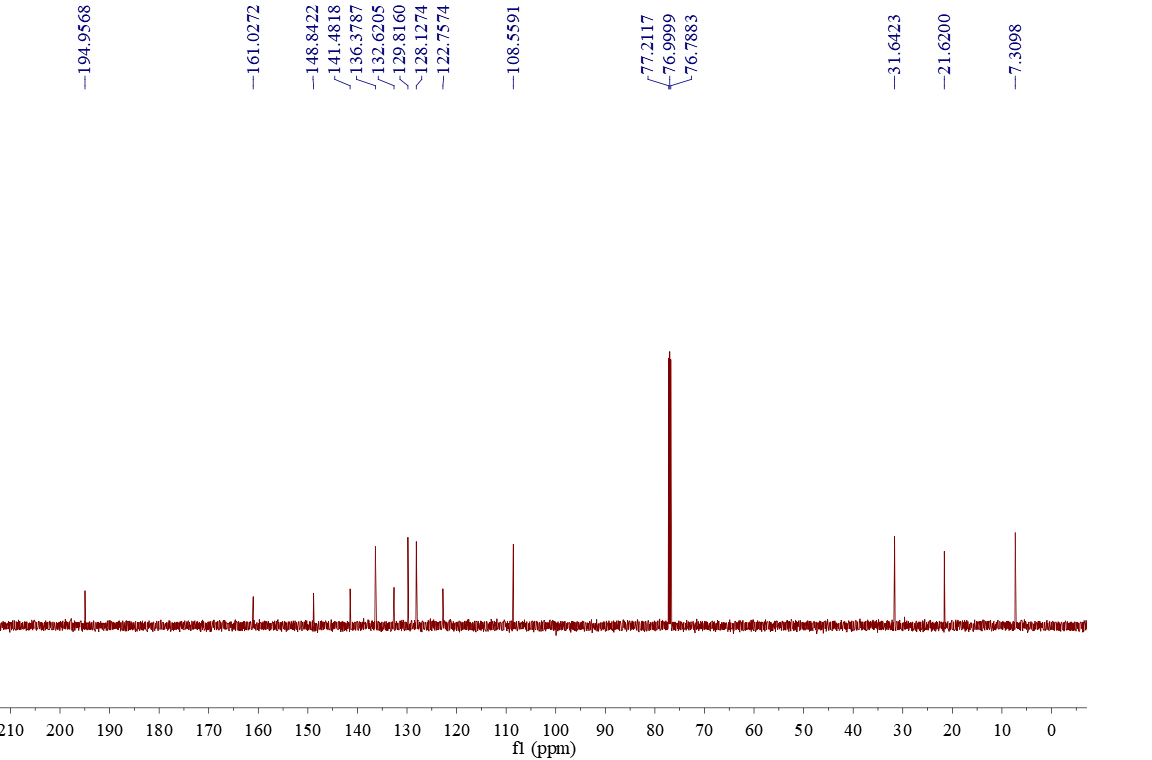
**

**Attached Fig.68** ^13^C NMR spectra of compound **3g**

**^
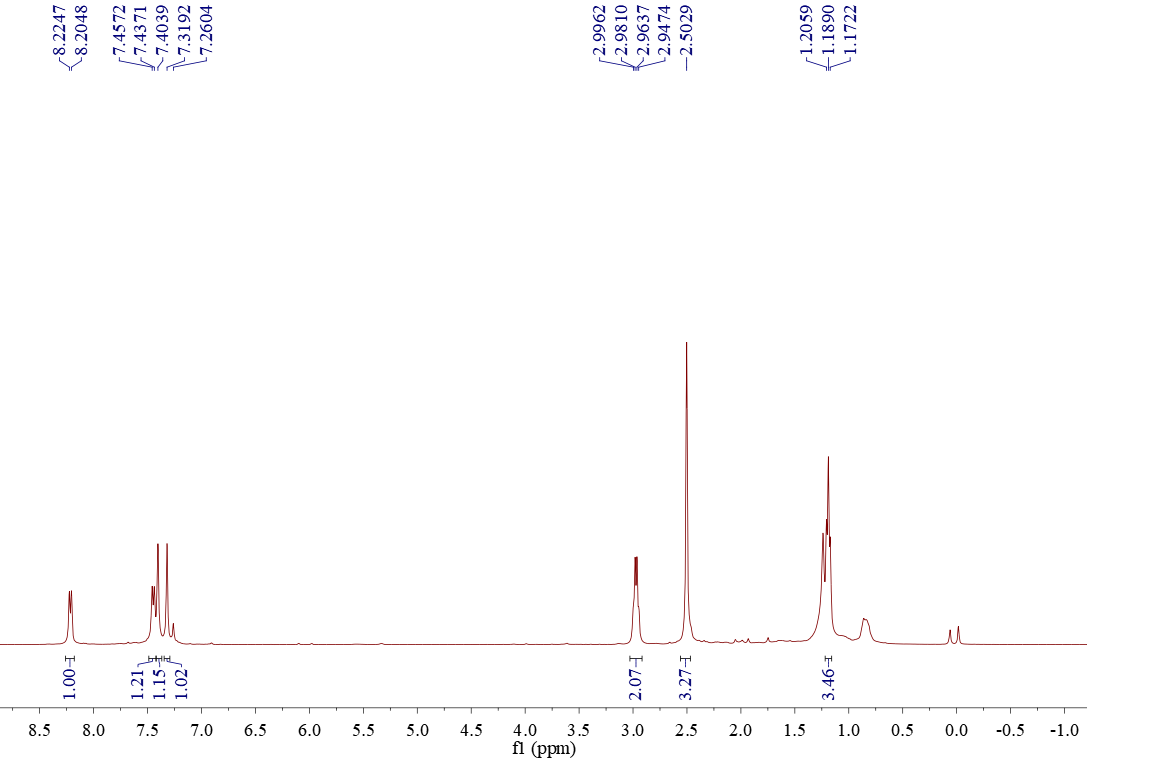
^**

**Attached Fig.69** ^1^H NMR spectra of compound **3h**

**^
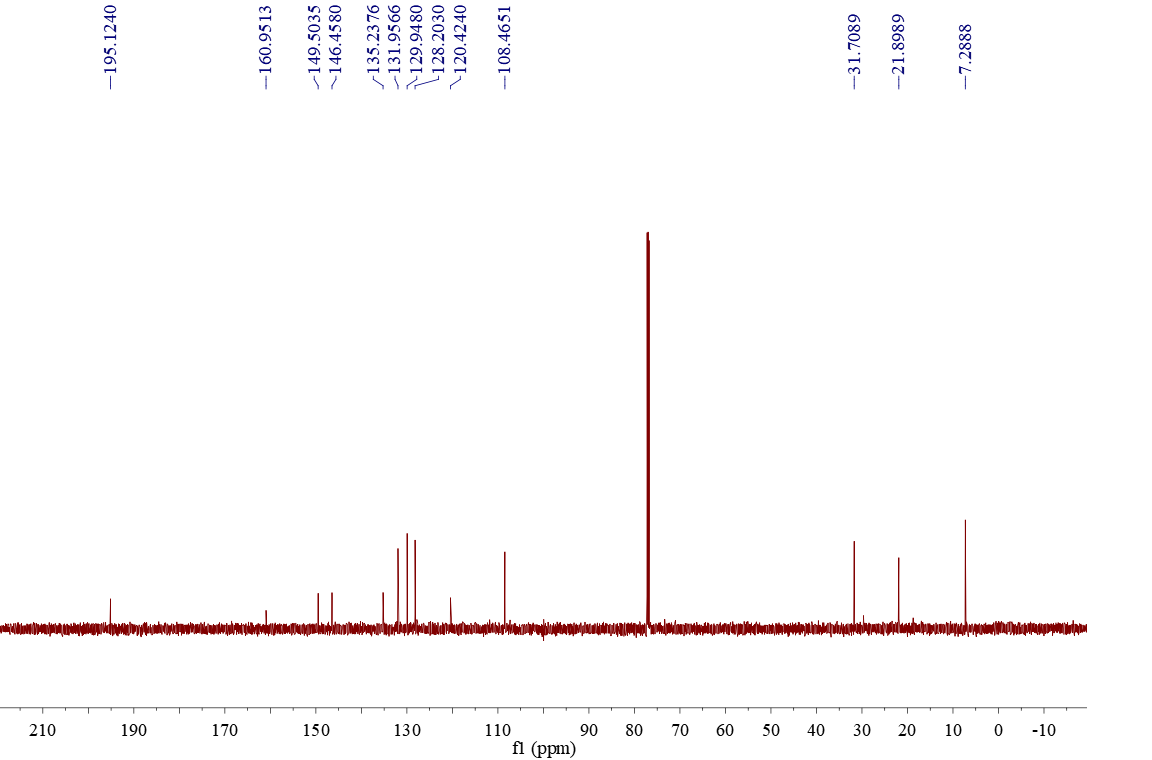
^**

**Attached Fig.70** ^13^C NMR spectra of compound **3h**

**
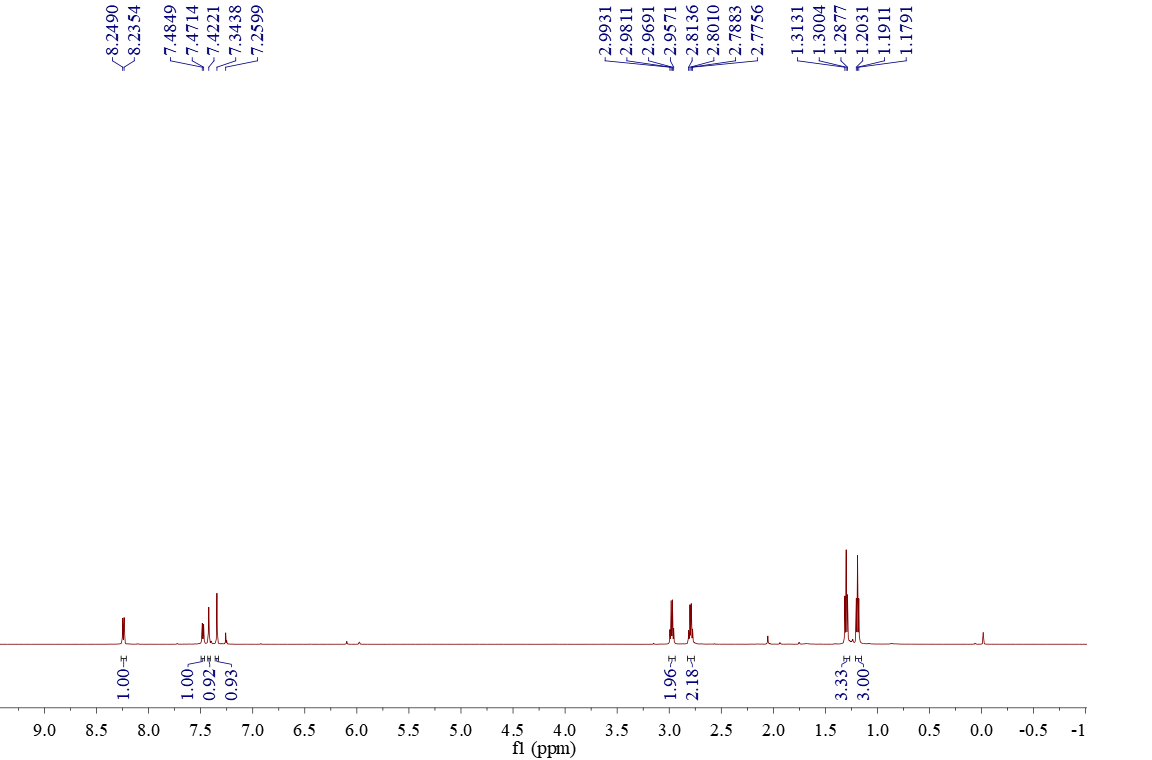
**

**Attached Fig.71** ^1^H NMR spectra of compound **3i**

**^
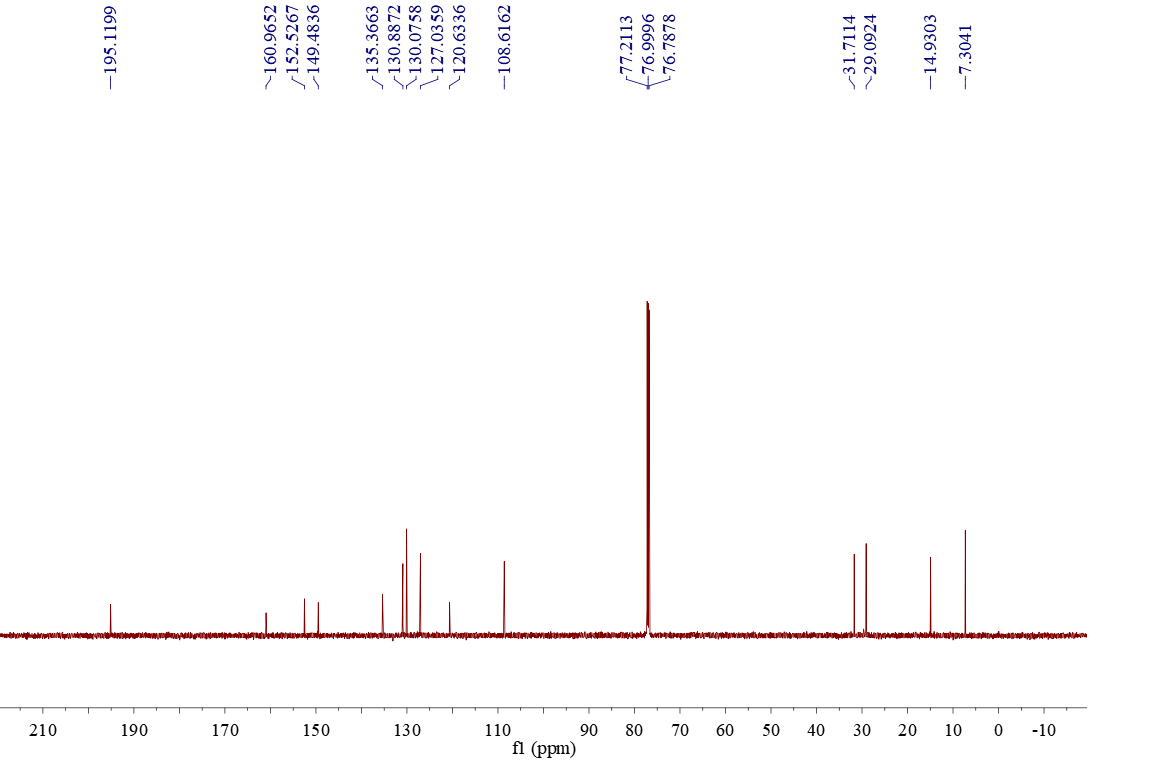
^**

**Attached Fig.72** ^13^C NMR spectra of compound **3i**

**
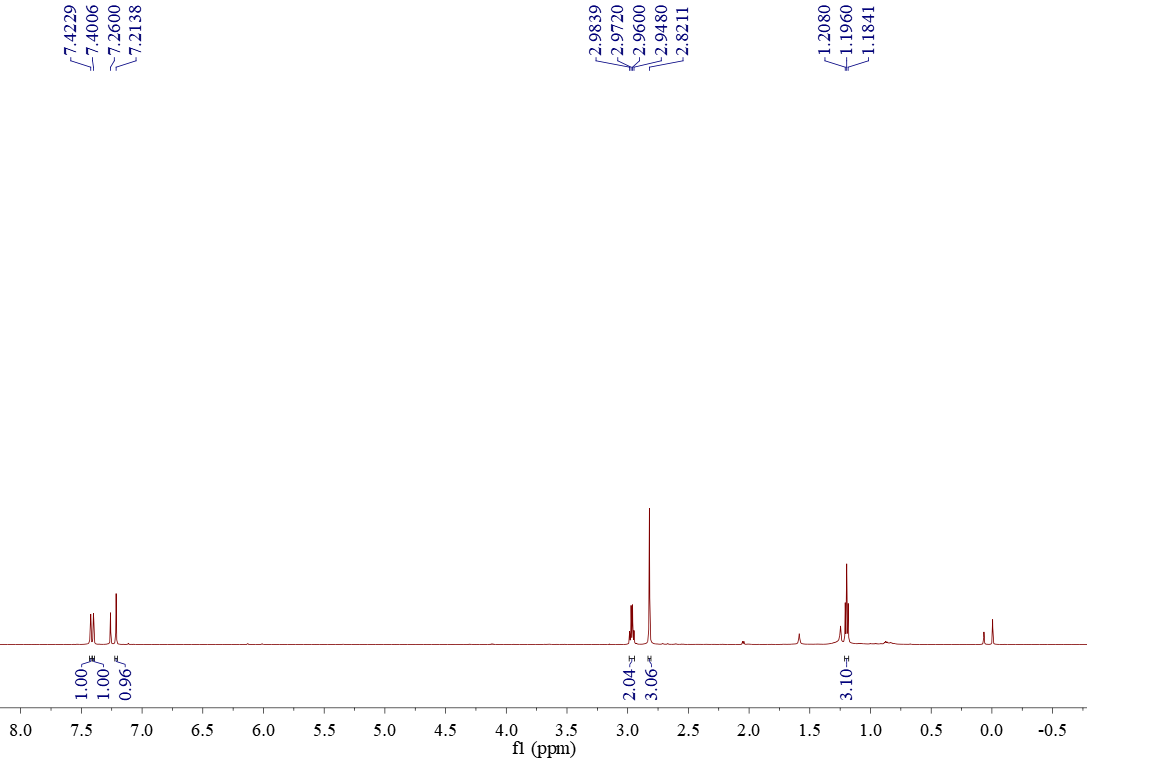
**

**Attached Fig.73** ^1^H NMR spectra of compound **3j**


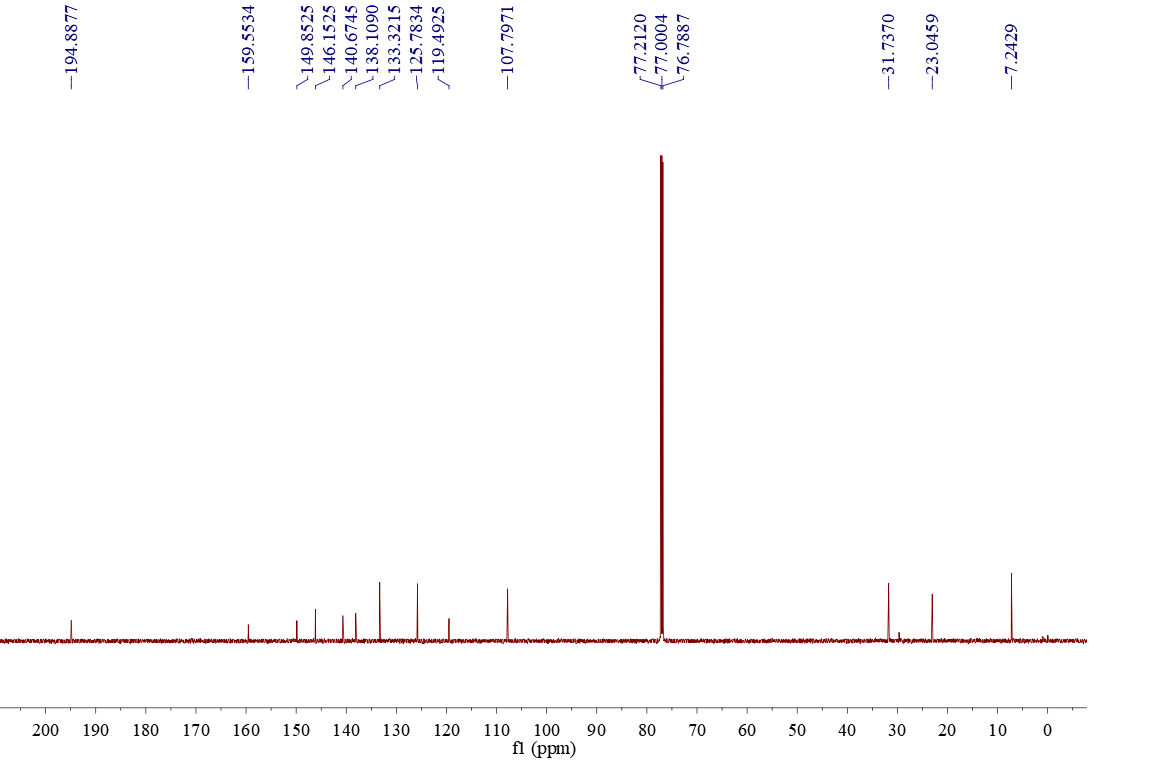


**Attached Fig.74** ^13^C NMR spectra of compound **3j**

**
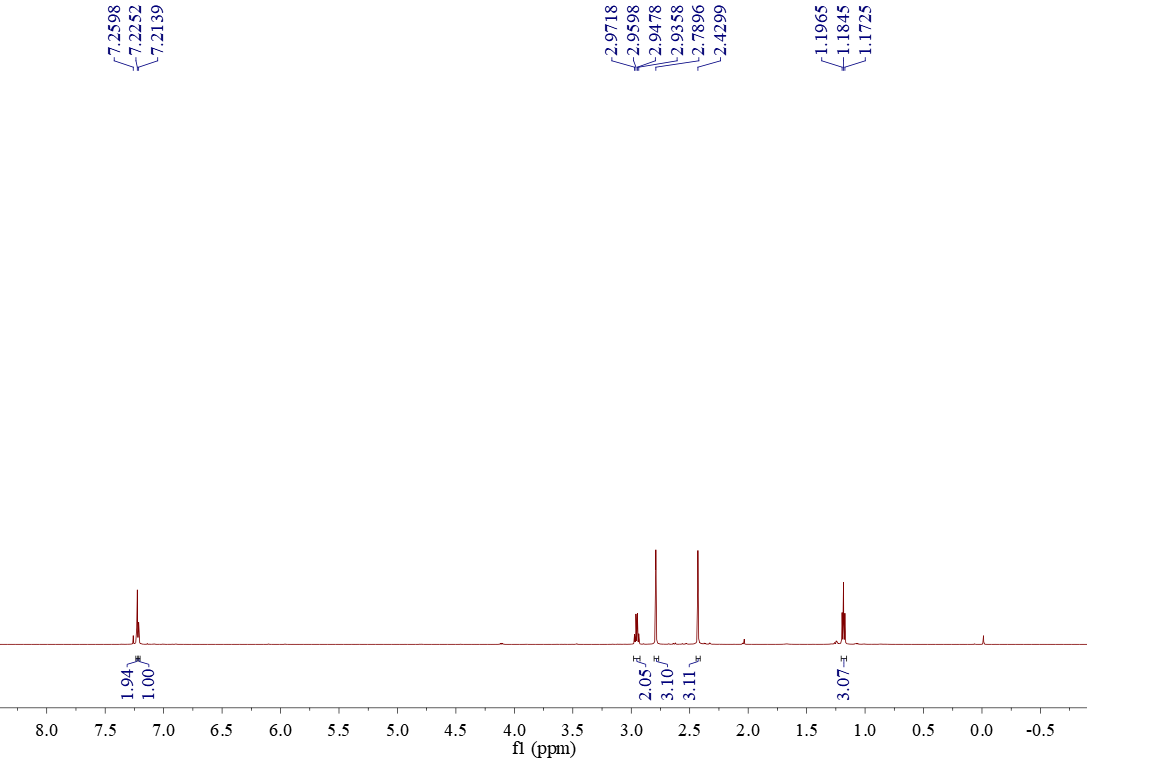
**

**Attached Fig.75** ^1^H NMR spectra of compound **3k**

**^
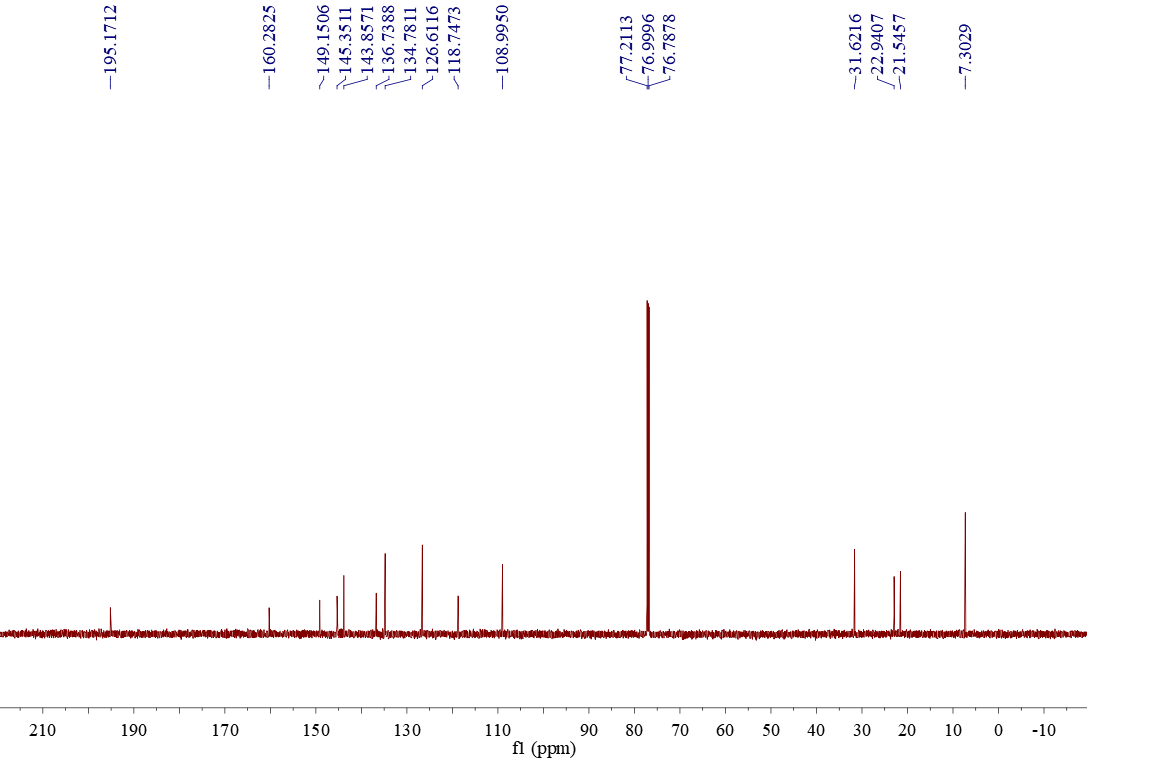
^**

**Attached Fig.76** ^13^C NMR spectra of compound **3k**

**^
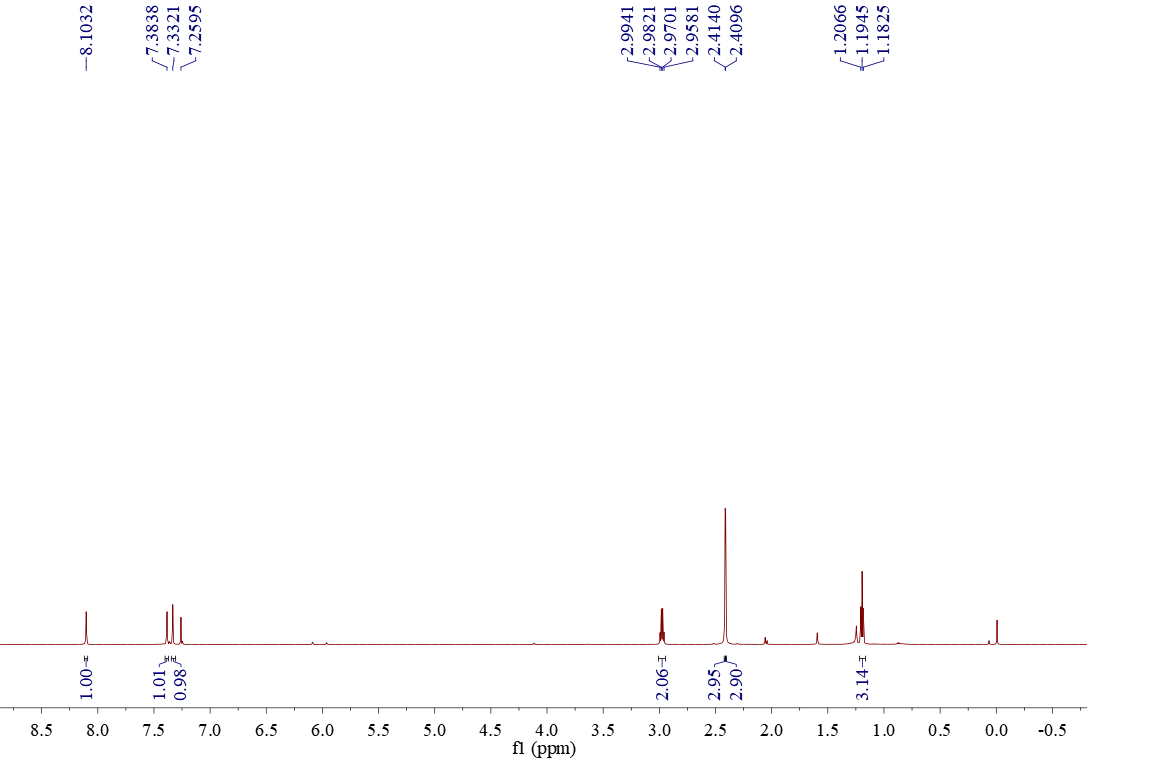
^**

**Attached Fig.77** ^1^H NMR spectra of compound **3l**

**^
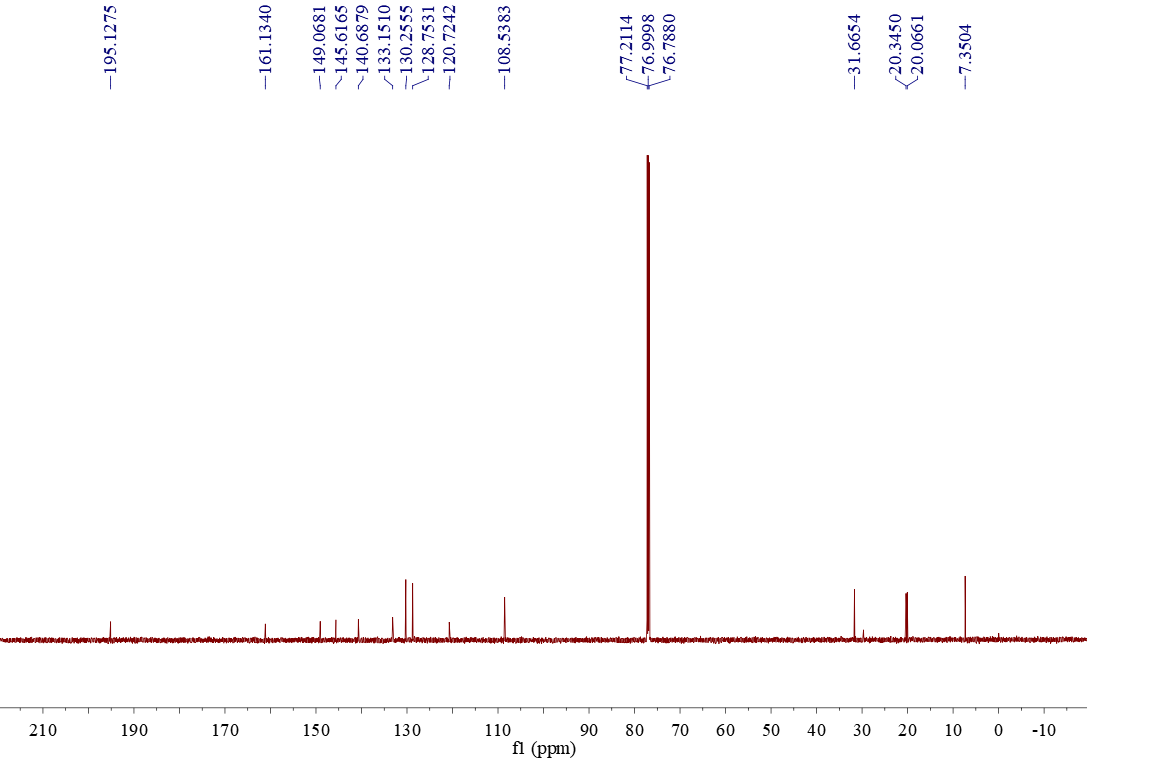
^**

**Attached Fig.78** ^13^C NMR spectra of compound **3l**

**^
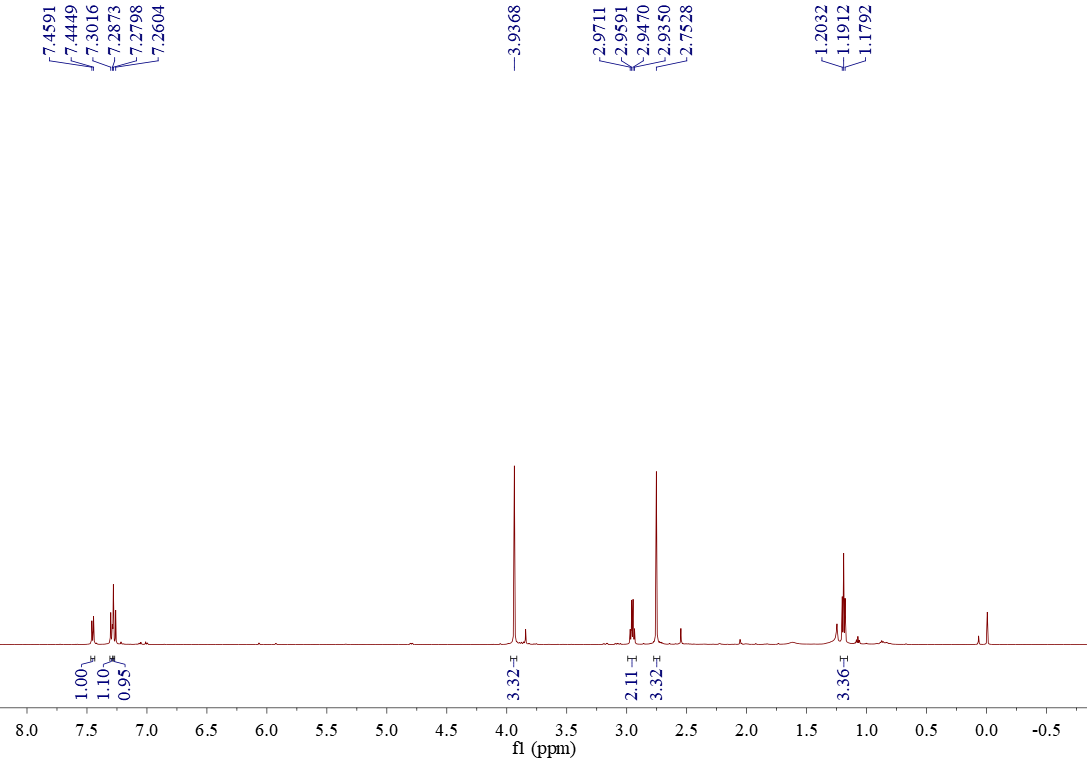
^**

**Attached Fig.81** ^1^H NMR spectra of compound **3m**

**^
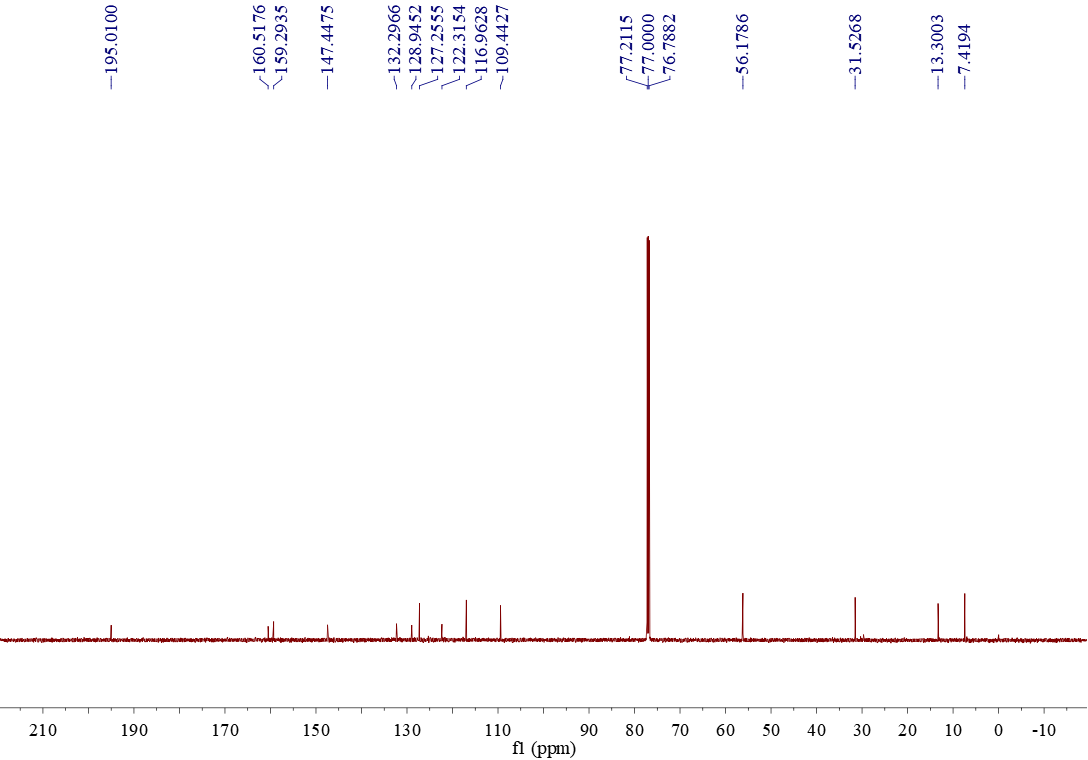
^**

**Attached Fig.82** ^13^C NMR spectra of compound **3m**

**^
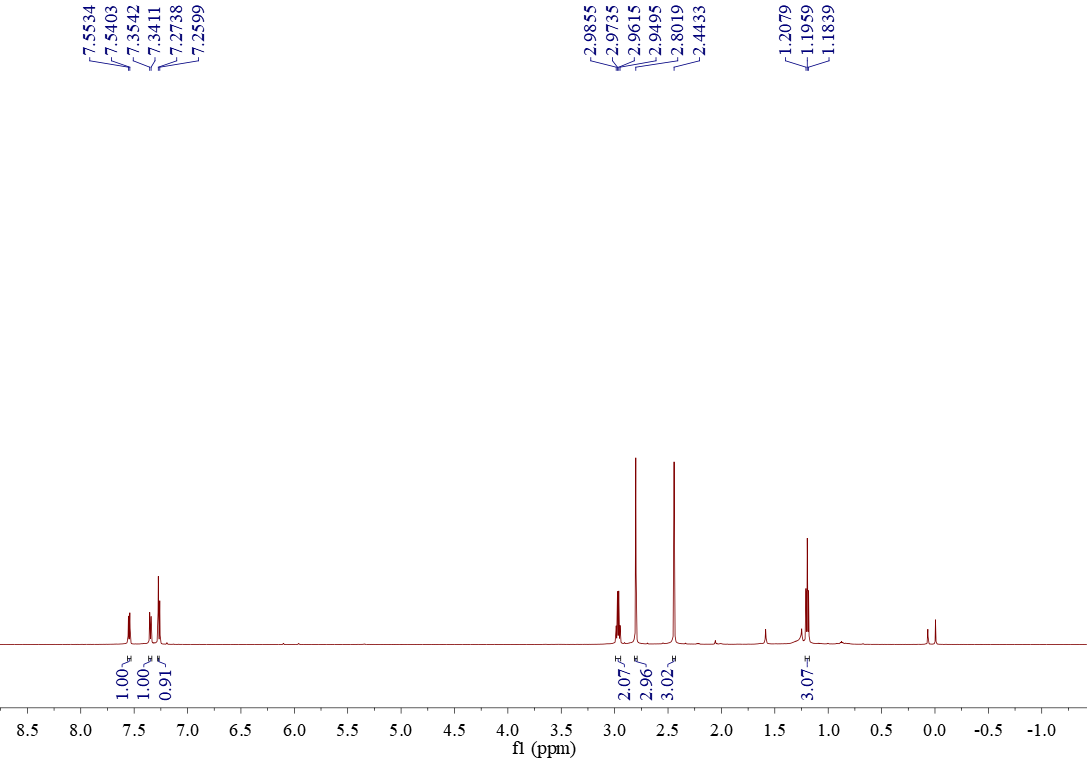
^**

**Attached Fig.83** ^1^H NMR spectra of compound **3n**

**^
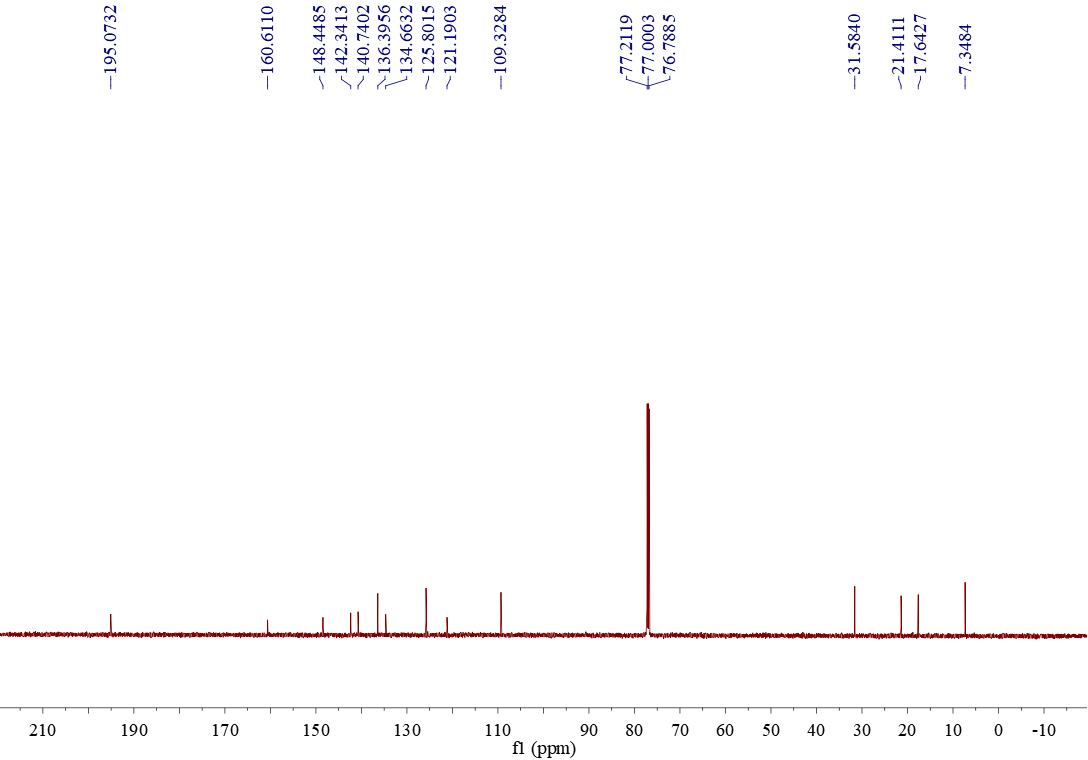
^**

**Attached Fig.84** ^13^C NMR spectra of compound **3n**

**^
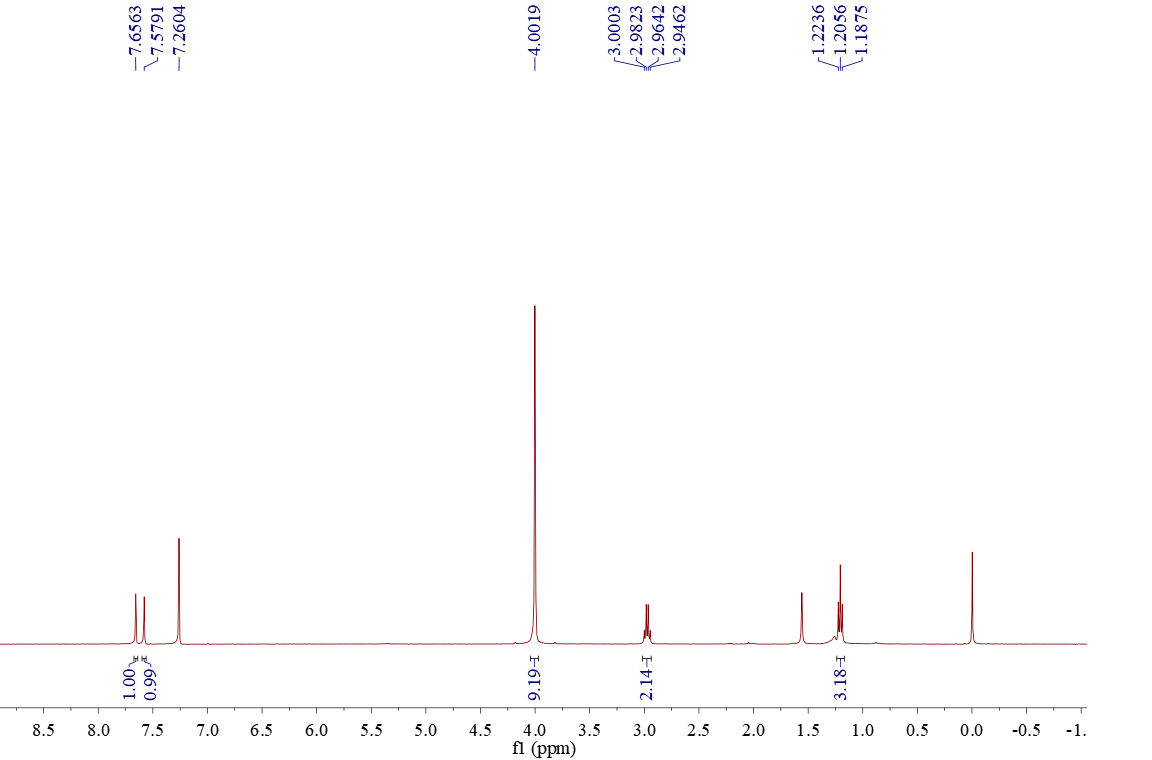
^**

**Attached Fig.79** ^1^H NMR spectra of compound **3o**

**^
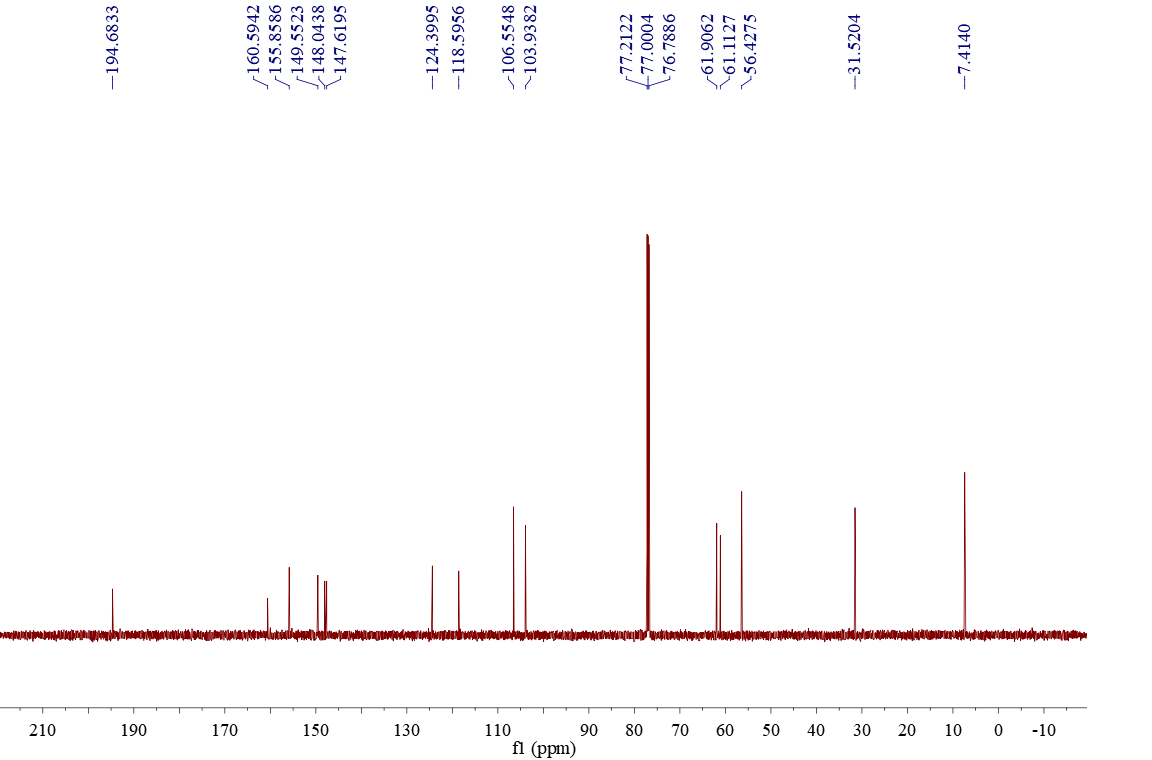
^**

**Attached Fig.80** ^13^C NMR spectra of compound **3o**

**^
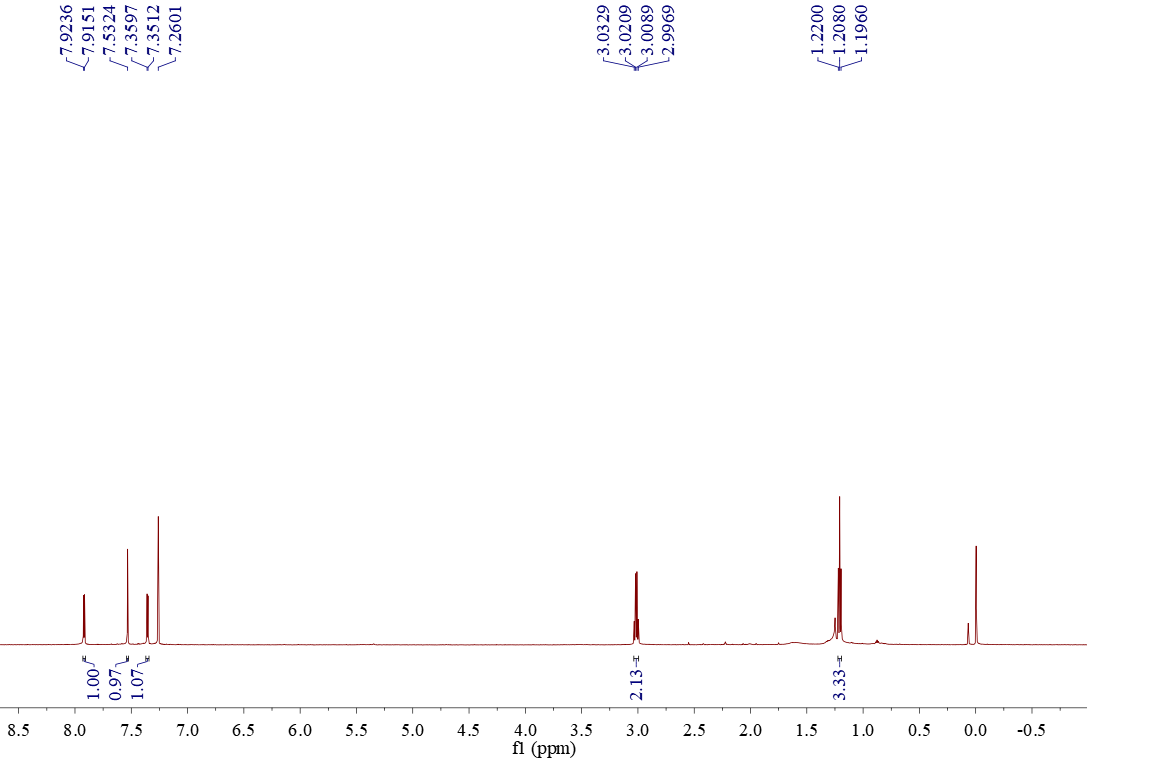
^**

**Attached Fig.85** ^1^H NMR spectra of compound **3p**

**^
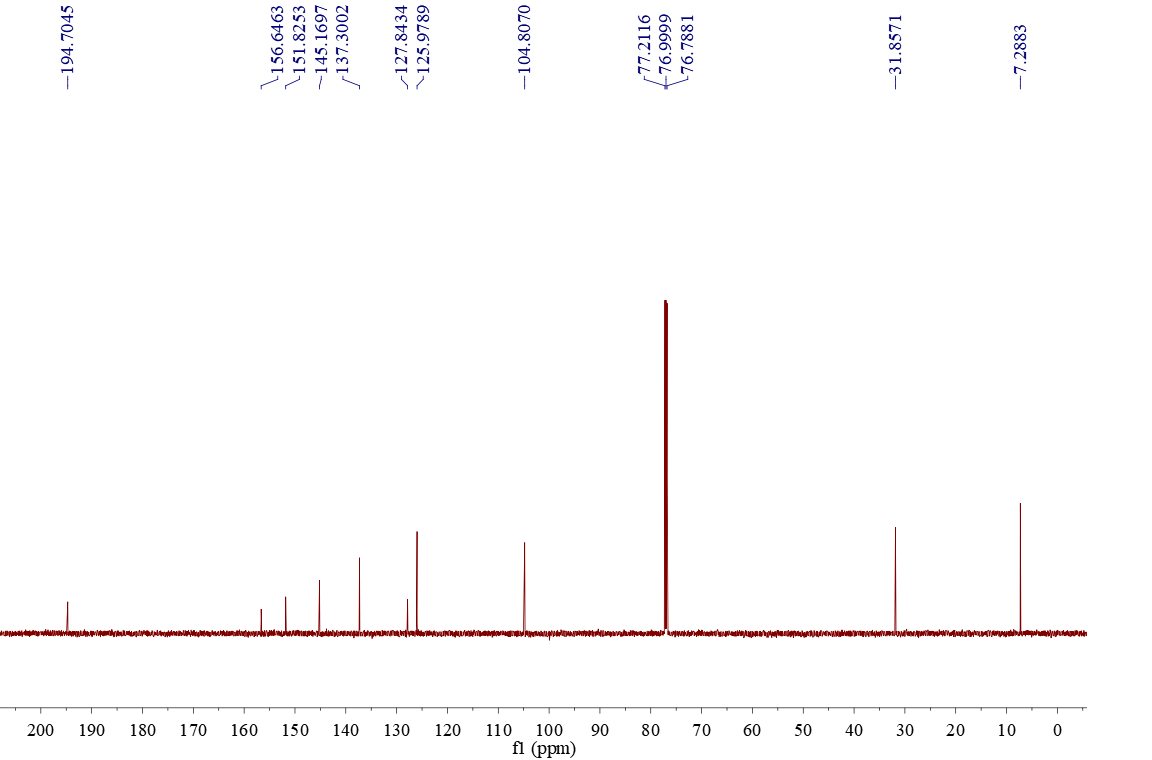
^**

**Attached Fig.86** ^13^C NMR spectra of compound **3p**

**^
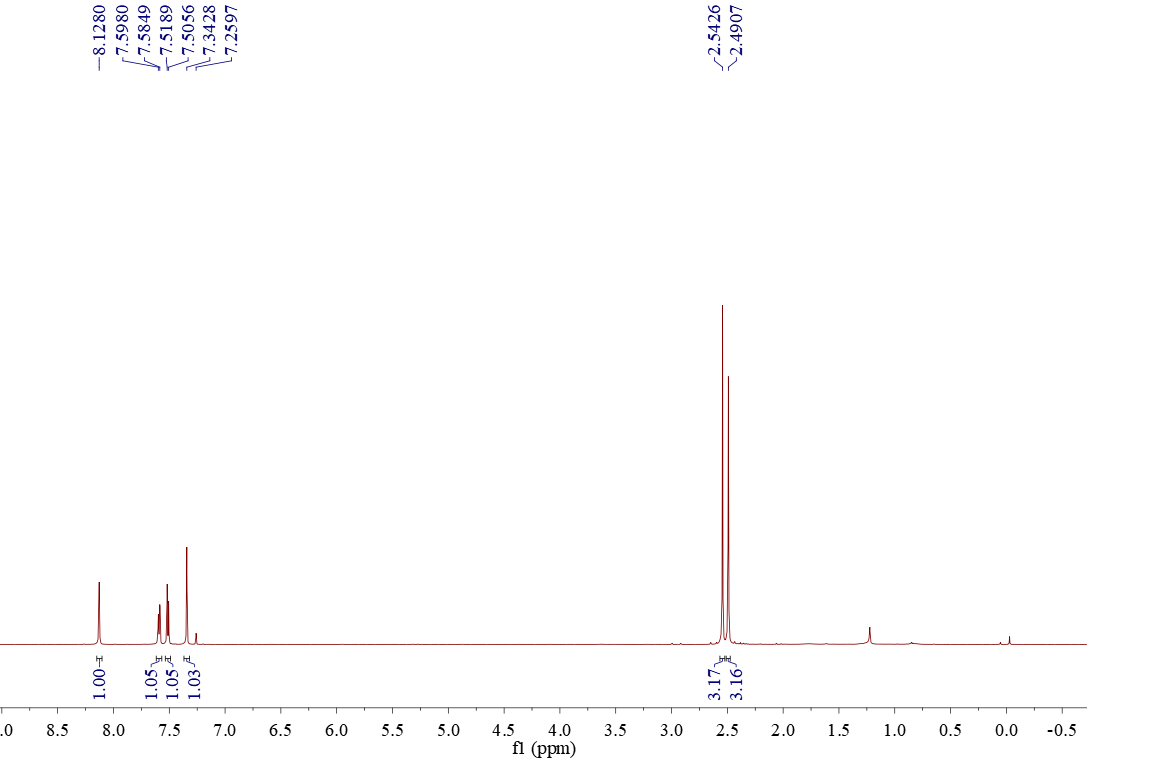
^**

**Attached Fig.87** ^1^H NMR spectra of compound **3q**

**^
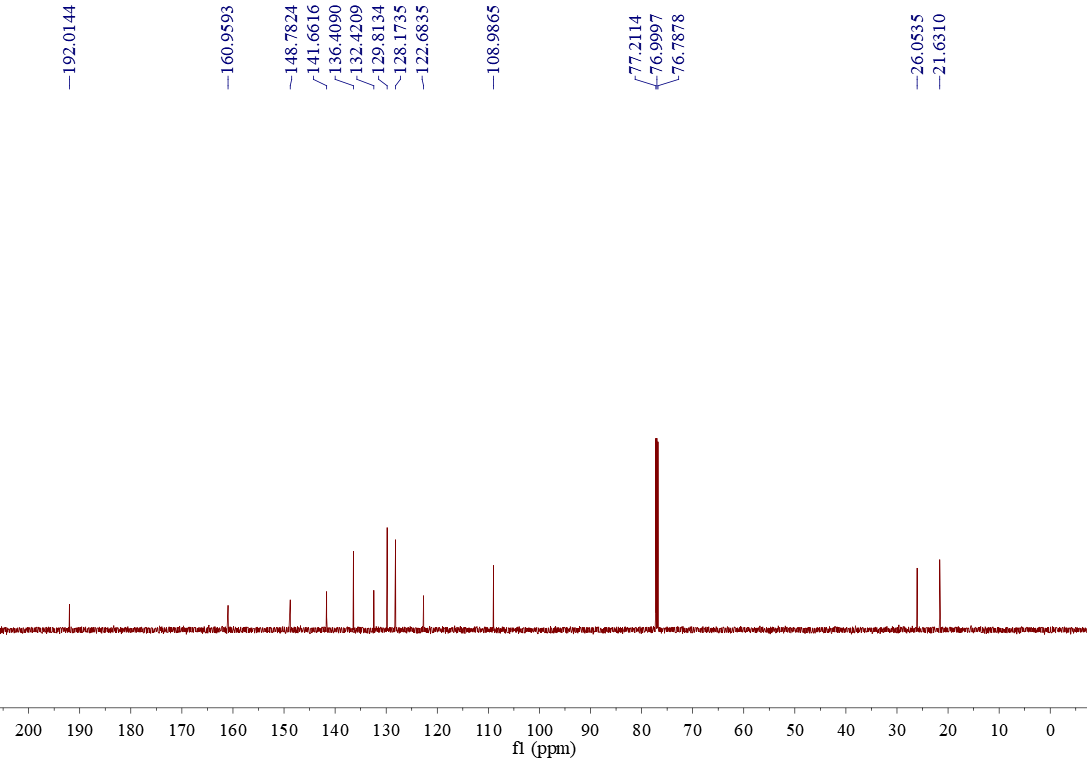
^**

**Attached Fig.88** ^13^C NMR spectra of compound **3q**

^
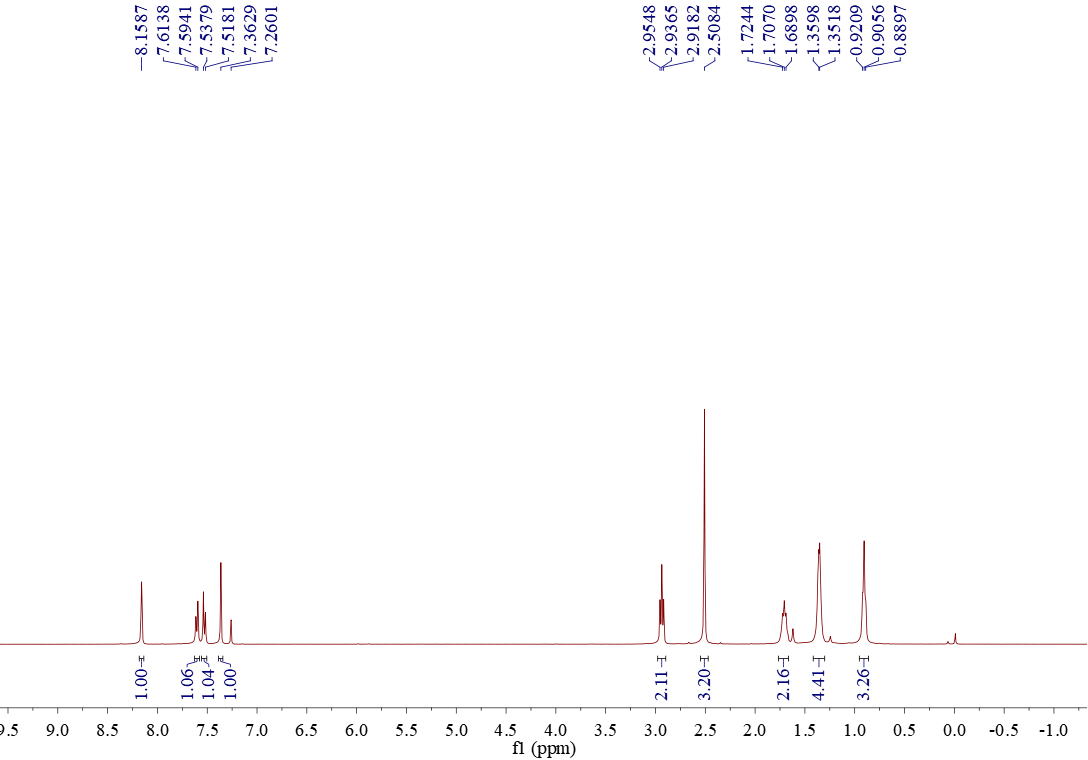
^

**Attached Fig.89** ^1^H NMR spectra of compound **3r**

**^
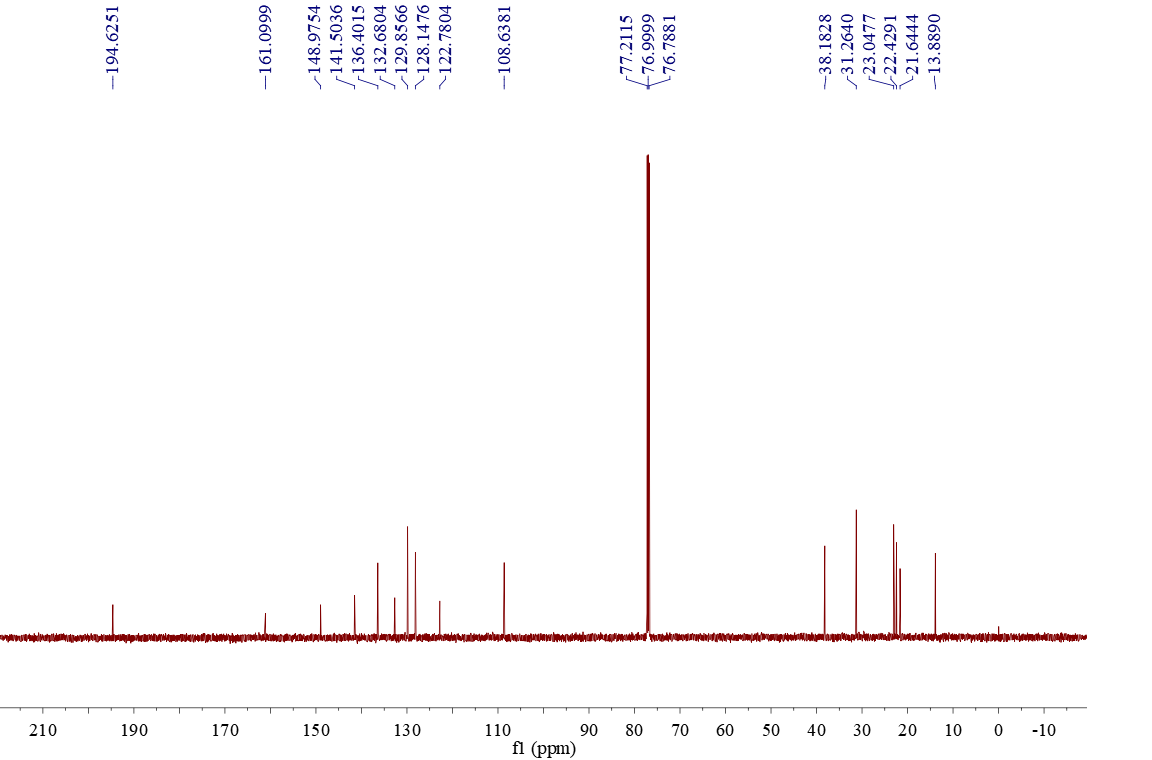
^**

**Attached Fig.90** ^13^C NMR spectra of compound **3r**
